# 2-(Fluoromethoxy)-4′-(*S*-methanesulfonimidoyl)-1,1′-biphenyl (UCM-1306), an Orally Bioavailable Positive Allosteric Modulator of the Human Dopamine D_1_ Receptor for Parkinson’s Disease

**DOI:** 10.1021/acs.jmedchem.2c00949

**Published:** 2022-08-31

**Authors:** Javier García-Cárceles, Henar Vázquez-Villa, José Brea, David Ladron de Guevara-Miranda, Giovanni Cincilla, Melchor Sánchez-Martínez, Anabel Sánchez-Merino, Sergio Algar, María Teresa de los Frailes, Richard S. Roberts, Juan A. Ballesteros, Fernando Rodríguez de Fonseca, Bellinda Benhamú, María I. Loza, María L. López-Rodríguez

**Affiliations:** † Departamento de Química Orgánica, Universidad Complutense de Madrid, E-28040 Madrid, Spain; ‡ Biofarma Research Group, USEF Screening Platform, CIMUS, USC, E-15782 Santiago de Compostela, Spain; § Instituto de Investigación Biomédica de Málaga (IBIMA), E-29010 Málaga, Spain; ∥ Molomics S.L., Parc Científic de Barcelona, Baldiri Reixac 4-8, E-08028 Barcelona, Spain; ⊥ Fundación Kærtor, Edificio EMPRENDIA, Planta 2, Oficina 4. Campus Vida, E-15706 Santiago de Compostela, Spain; # Vivia Biotech S.L., Parque Científico de Madrid, E-28760 Madrid, Spain

## Abstract

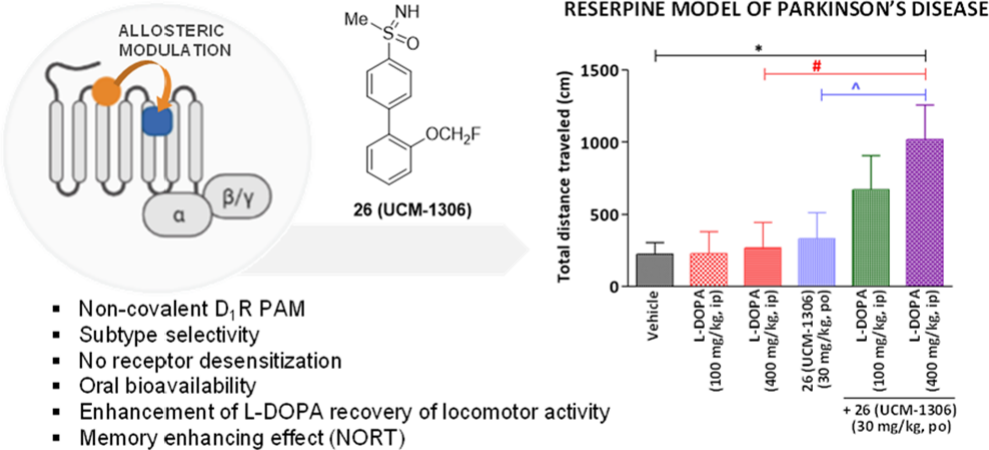

Tolerance development caused by dopamine replacement with l-DOPA and therapeutic drawbacks upon activation of dopaminergic receptors with orthosteric agonists reveal a significant unmet need for safe and effective treatment of Parkinson’s disease. In search for selective modulators of the D_1_ receptor, the screening of a chemical library and subsequent medicinal chemistry program around an identified hit resulted in new synthetic compound **26** [UCM-1306, 2-(fluoromethoxy)-4′-(*S*-methanesulfonimidoyl)-1,1′-biphenyl] that increases the dopamine maximal effect in a dose-dependent manner in human and mouse D_1_ receptors, is inactive in the absence of dopamine, modulates dopamine affinity for the receptor, exhibits subtype selectivity, and displays low binding competition with orthosteric ligands. The new allosteric modulator potentiates cocaine-induced locomotion and enhances l-DOPA recovery of decreased locomotor activity in reserpinized mice after oral administration. The behavior of compound **26** supports the interest of a positive allosteric modulator of the D_1_ receptor as a promising therapeutic approach for Parkinson’s disease.

## Introduction

Parkinson’s disease (PD) is a chronic, progressive, neurodegenerative disorder characterized by the loss of dopaminergic neurons in the brain. In addition to the main symptoms derived from motor dysfunction, PD is associated with memory loss and eventually dementia, due to the disruptive effect on dopaminergic transmission in the hippocampus and the prefrontal cortex.^1,2^ Dopamine (DA) replacement with l-DOPA was proposed about 60 years ago^3^ and is still the primarily used therapy and most effective treatment against the debilitating motor symptoms of PD. However, in the long term, the therapeutic index of l-DOPA decreases and its anti-Parkinsonian action is very often associated with progressive decline in symptomatic benefit (motor fluctuations), development of dyskinesia, and other adverse effects, including hypotension, hallucinations, and gastrointestinal disturbances.^4,5^ Due to l-DOPA tolerance development, an alternative approach for PD treatment is based on the dopaminergic stimulation using agonists that activate the DA receptors.^6^ These are members of a family belonging to the class A of the G-protein coupled receptors (GPCRs) and are subdivided into two groups: D_1_-like subtypes (D_1_ and D_5_) couple to G_s_ and G_olf_ proteins stimulating the production of the second messenger cAMP, and D_2_-like subtypes (D_2_, D_3_, and D_4_) couple to G_i_ inhibiting cAMP production.^7,8^ The D_1_ and D_2_ receptors are the most abundant subtypes in the brain and have thus been the most studied. In particular, the D_1_ receptor (D_1_R) has not only well-documented roles in motor activity but also in memory function.^9,10^ Hence, activation of the D_1_R with orthosteric agonists has been a target for drug discovery efforts to develop improved therapies for movement disorders in PD and cognitive decline associated with PD and other neuropsychiatric pathologies such as schizophrenia, Alzheimer’s disease, and other forms of dementia.^11−14^ A number of these agonists are endowed with anti-Parkinsonian properties and have been strongly validated at the preclinical stage.^6,11^ However, D_1_R orthosteric agonists suffer from numerous therapeutic drawbacks including: low selectivity over other DA receptors; poor pharmacokinetic profile; inverted U-shaped dose response, probably due to overstimulation at higher dose; rapid onset of tolerance and desensitization caused by a constant activation of the receptor; and tolerability issues, such as hypotension and dyskinesia.^12^ Hence, their clinical development has been very challenging and largely unsuccessful so far, and there remains a significant unmet need for safe and effective treatment of PD.

**Figure 1:**
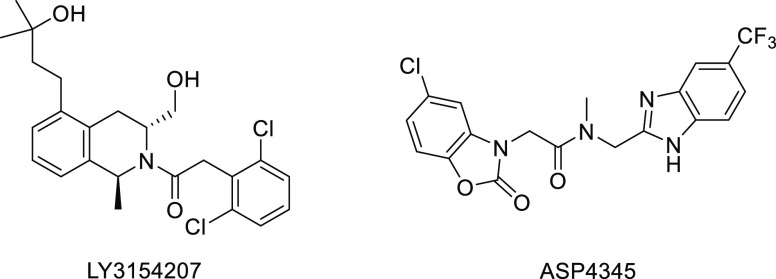
Structures of positive allosteric modulators of the dopamine D_1_R tested in clinical trials.

In recent years, the allosteric modulation approach has emerged as a new and alternative strategy to regulate GPCR functions, and the development of allosteric ligands has received widespread attention in drug discovery programs targeting numerous receptors.^15−25^ The novel compounds bind at allosteric sites that are spatially distinct from the orthosteric site where the endogenous, natural ligand does. Binding of a ligand to the allosteric site induces conformational changes of the receptor, which results in the modulation of the affinity, potency, and/or signal transduction efficacy of the endogenous/orthosteric ligand response. The allosteric ligand can be classified as a positive allosteric modulator (PAM, enhancing signaling), a negative allosteric modulator (NAM, reducing signaling), or a silent allosteric modulator (SAM, no effect on signaling).^26^ GPCR allosteric modulators present unique advantages over orthosteric classical ligands including: a higher pharmacological selectivity, due to the greater structural diversity hypothesized for allosteric sites in contrast to the conserved nature of the orthosteric site among related GPCRs; and a more physiological effect because they exert their indirect action only in the presence of the endogenous ligands, which limits the action of the allosteric ligand producing a saturability of the effect (“ceiling effect”) and protecting against a potential overdose of an orthosteric ligand.^27−30^ Hence, allosteric modulation has become a promising approach toward the discovery of safer drugs that offer the maximum benefit while minimizing side effects. However, the development of allosteric modulators for GPCRs has been challenging and has afforded few FDA-approved drugs: the calcium-sensing receptor PAMs cinacalcet and etelcalcetide, the CCR5 receptor NAM maraviroc, the CXCR4 NAM/CXCR7 allosteric agonist plerixafor, the smoothened receptor NAM vismodegib, and the GABA_A_ receptor PAM brexanolone.^31−36^ In this context, the positive allosteric modulation approach to upregulate D_1_R activity has been recently proposed as a novel strategy toward improved dopaminergic therapies for PD. Rather than directly activating the D_1_R, a PAM will potentiate the action of endogenous DA, thus inducing a more physiological mode of action and avoiding the pitfalls displayed by orthosteric ligands such as overstimulation, tolerance development, and low tolerability.^11,37^ However, this therapeutic potential has not been clinically assessed for disclosed D_1_R PAMs.^11^ Among them, LY3154207 and ASP4345 (Figure 1) have reached phase 2 development for the treatment of PD and schizophrenia, respectively, which will hopefully validate the clinical use of a D_1_R PAM.^38−40^

**Figure 2:**
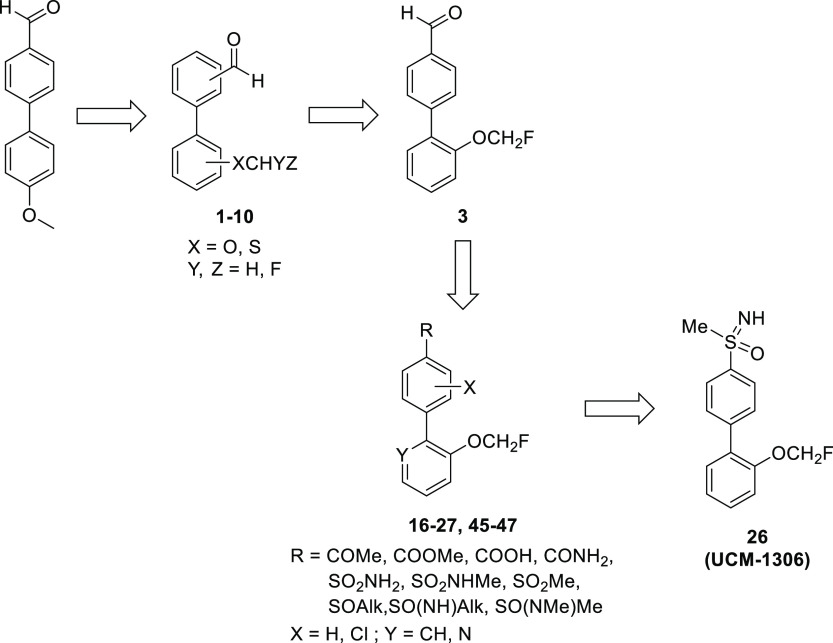
Search of new synthetic modulators of the D_1_R starting from the initial identified hit.

In the present work, we contribute to the highly desired search for selectively acting D_1_R allosteric modulators. The screening of an in-house chemical library using a potentiatior-mode cAMP assay in a cell line stably expressing the human D_1_R, followed by structural modification of identified hit 4′-methoxy[1,1′-biphenyl]-4-carbaldehyde, has led to new biphenyl derivative **3** characterized in vitro as a D_1_R PAM (Figure 2). A subsequent medicinal chemistry program resulted in compound **26** (UCM-1306, Figure 2), a non-covalent PAM that increases the endogenous DA maximal effect both in human and mouse D_1_ receptors, does not induce receptor desensitization, exhibits no agonist activity and subtype selectivity, and when orally administered it potentiates l-DOPA recovery of decreased locomotor activity in a preclinical model of PD. The compound enhances memory in a novel object recognition test (NORT), supporting its additional use in complicated PD patients with cognitive impairment.

## Results and Discussion

### Identification of Compound **3**

An in-house chemical library of 250 compounds was screened using an in vitro assay measuring accumulation of cAMP in a human neuroblastoma SK-N-MC cell line stably expressing physiological levels of the D_1_R but not the D_5_R.^41^ To assess allosteric modulation of the D_1_R, cells were treated with a fixed concentration of the test compounds (10 μM) for 15 min and then co-incubated with increasing concentrations of the endogenous agonist DA. The cAMP concentration was quantified by homogeneous time-resolved fluorescence energy transfer (HTRF) and the effect of each compound on the DA concentration–response curve was measured. A compound inducing a potentiation >20% in the DA maximal effect (measured as *E*_max_) was considered a potential PAM of the receptor. Using this potentiatior-mode cAMP assay, 4′-methoxy[1,1′-biphenyl]-4-carbaldehyde, exhibiting the highest increase in the DA *E*_max_ (55%, Figure 2), was identified as an initial hit for the search of new synthetic allosteric modulators of the D_1_R.

**Scheme 1:**
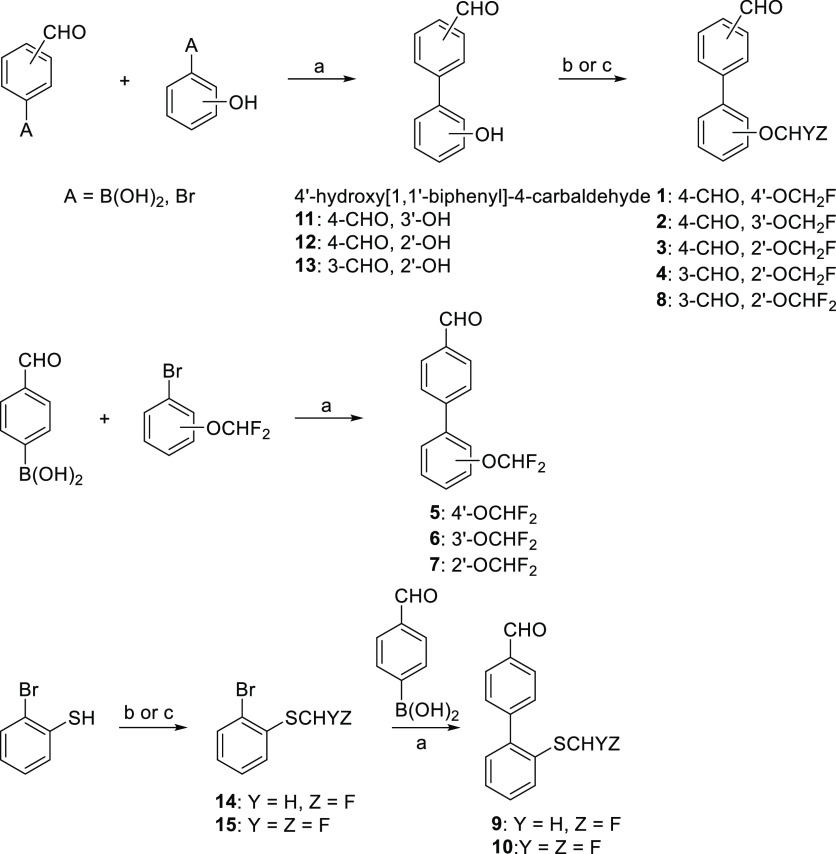
Synthesis of Compounds **1–10** Reagents and conditions: (a) Pd(PPh_3_)_4_, Na_2_CO_3_, toluene/EtOH/H_2_O or THF/H_2_O, MW, 100/120/130 °C, 20 min or Δ, on, 49–90%; (b) ClCH_2_F (2.0 M in DMF), Cs_2_CO_3_, DMF, −78 °C to rt, on, 59–84%; (c) BrF_2_CP(O)(OEt)_2_, KOH, ACN/H_2_O, −78 °C to rt, on, 40–81%.

Starting from this hit, related compounds **1–10** (Figure 2) were proposed to explore the substitution pattern on the biphenyl scaffold as well as to produce structurally novel compounds by the modification of the methoxy group.^42^ For the synthesis of biphenyl analogues **1–10**, a strategy based on Suzuki–Miyaura coupling and fluoroalkylation reaction was followed (Scheme 1). Thus, coupling between the appropriate arylboronic acids and bromobenzene derivatives was carried out using Pd(PPh_3_)_4_ as the catalyst under microwave (MW) irradiation or thermal conditions to afford intermediates **11–13**. Next, alkylation of these intermediates or commercial 4′-hydroxy[1,1′biphenyl]-4-carbaldehyde with chlorofluoromethane allowed to obtain monofluoromethoxy derivatives **1–4**. Difluoromethoxy analogues **5–7** were readily synthesized by the coupling reaction between (4-formylphenyl)boronic acid and the corresponding commercially available bromodifluoromethoxybenzene, whereas compound **8** was prepared following the Suzuki–Miyaura-fluoroalkylation sequence, using diethyl [bromo(difluoro)methyl]phosphonate as an alkylating reagent. It should be noted that compounds bearing formyl and alkoxy groups in 2- and 2′-position were not considered because both 2′-hydroxy[1,1′-biphenyl]-2-carbaldehyde and 2′-alkoxy[1,1′-biphenyl]-2-carbaldehyde are reported to undergo ring-closure to form the corresponding hemiacetal or acetal, respectively.^43^ In the case of alkylsulfanyl derivatives **9** and **10**, direct Suzuki–Miyaura reaction using 2-bromobenzenethiol as a starting material did not work. Hence, mono- or difluoroalkylation of 2-bromobenzenethiol was first carried out to obtain intermediates **14** and **15**, respectively, which afforded final compounds **9** and **10** by coupling reaction with (4-formylphenyl)boronic acid (Scheme 1).

Potential PAM activities of synthesized compounds **1–10** were tested in the potentiator-mode cAMP production assay, and the results of D_1_R activation are summarized in Table 1. The effect observed for the compounds at a fixed concentration of 10 μM over DA concentration–response curves revealed a potentiation of the DA *E*_max_. Based on the observation that the compounds did not modify DA EC_50_ but they increased DA *E*_max_, a concentration–response curve in the presence of DA EC_70_ concentration was built for those compounds that enhanced more than 30% the DA *E*_max_. The PAM efficacy was measured as the percentage of increase over DA EC_70_ and the potency as the EC_50_ observed in the concentration–response curve. Compounds **1–3** and **5–7** revealed that the 2′-position is the most favorable for the alkoxy group (**3** and **7**, with DA potentiation of 82 and 45% at 10 μM, respectively). Hence, the position of the formyl group was explored in 2′-fluoroalkoxy analogues **4** and **8**, revealing a marked drop of activity when the carbonyl is situated in the 3-position (DA potentiation of 23 and 12% at 10 μM, respectively). Compounds **3** and **7**, bearing a formyl group in the 4-position and a fluoroalkoxy moiety in the 2′-position, were also the best potentiators when tested in the presence of an EC_70_ concentration of DA (91 and 62%, respectively, Table 1). Replacement of oxygen for a sulfur atom in the alkoxy moiety did not improve the allosteric modulation in derivative **10** (44% potentiation at 10 μM, Table 1), and a marked drop was observed in analogue **9** (14% potentiation at 10 μM, Table 1).

**Table 1:**
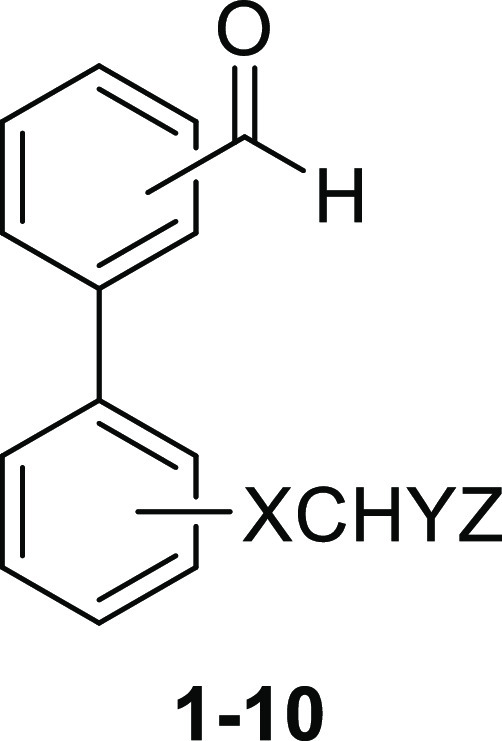
Effect of Compounds **1–10** in DA-induced cAMP Production in Human D_1_R Endogenously Expressed in a Neuroblastoma Cell Line

compd	CHO position	XCHYZ position	X	Y, Z	potentiation of DA *E*_max_ (%)^a^	% maximum increase^b^	EC_50_ (μM)^c^
**1**	4	4′	O	H, F	29 ± 6	nd	nd
**2**	4	3′	O	H, F	25 ± 9	nd	nd
**3**	4	2′	O	H, F	82 ± 8	91 ± 6	12.7 ± 2.4
**4**	3	2′	O	H, F	23 ± 5	nd	nd
**5**	4	4′	O	F, F	35 ± 8	37 ± 5	2.3 ± 0.4
**6**	4	3′	O	F, F	37 ± 6	35 ± 7	10.8 ± 1.6
**7**	4	2′	O	F, F	45 ± 5	62 ± 10	18.5 ± 2.7
**8**	3	2′	O	F, F	12 ± 2	nd	nd
**9**	4	2′	S	H, F	14 ± 3	nd	nd
**10**	4	2′	S	F, F	44 ± 5	101 ± 10	21.8 ± 3.6

a Effect over the DA concentration–response curve at a fixed concentration of compound = 10 μM.

b Efficacy (measured as % of maximum increase over DA EC_70_) in the concentration–response curves of the compounds over DA EC_70_.

c Potency (measured as EC_50_) of the compounds at the DA EC_70_ concentration; values are the mean ± SEM of three independent experiments with duplicate determinations; nd = not determined.

Clearly, among the newly identified modulators of the D_1_R, compound **3** is the most efficient PAM, potentiating DA *E*_max_ in 82% at 10 μM. Further pharmacological evaluation in the potentiator-mode cAMP assay at different fixed concentrations (0.1–10 μM) resulted in a concentration-dependent potentiation of DA effect in human SK-N-MC and mouse CAD neuroblastoma cell lines (Figure S1A,B), and it enhanced EC_70_ DA effect in 91%, with an EC_50_ of 12.7 μM (Table 1 and Figure S1C). No cAMP response to compound **3** was observed in the absence of DA, indicating that the modulator behaved mostly as a potentiator with no agonist activity (Figure S1D). In order to ascertain if this allosteric potentiation was due to a modulation on DA affinity, radioligand binding assays in the presence of DA IC_50_ (4 μM) were carried out. The calculation of the affinity ratio of [^3^H]SCH-23390^44^ showed a concentration-dependent effect of compound **3** by increasing the affinity ratio values, which evidences a positive allosteric behavior of this compound over the DA effect (Figure S2A). When tested in transfected cells expressing human D_2_, D_3_, D_4_, or D_5_ receptors, compound **3** (10 μM) did not enhance DA *E*_max_ in the potentiator-mode cAMP assay (Figure S3). Results in competitive binding assays revealed that **3** displayed marginal displacement (12% at 10 μM) of the radioligand [^3^H]SCH-23390, whereas full displacement was observed for orthosteric agonist haloperidol (*K*_i_ = 5.5 nM, Figure S4). This confirms that modulator **3** does not bind to the high-sequence homology orthosteric site but should be located in an allosteric one, in agreement with the observed subtype selectivity.

Based on the pharmacological characterization of compound **3** as a specific PAM of the human D_1_R, the next step was an in vitro ADMET profiling (Table S1). Overall, the compound showed good permeability and low hERG inhibition, but its low solubility, high HSA binding, low serum stability, and moderate inhibition in a panel of CYP450 hampered in vivo validation of the therapeutic interest of compound **3** and prompted us to search for a new compound with improved pharmacokinetic properties.

### D_1_R PAM Optimization: from Compound **3** to Compound **26**

**Scheme 2:**
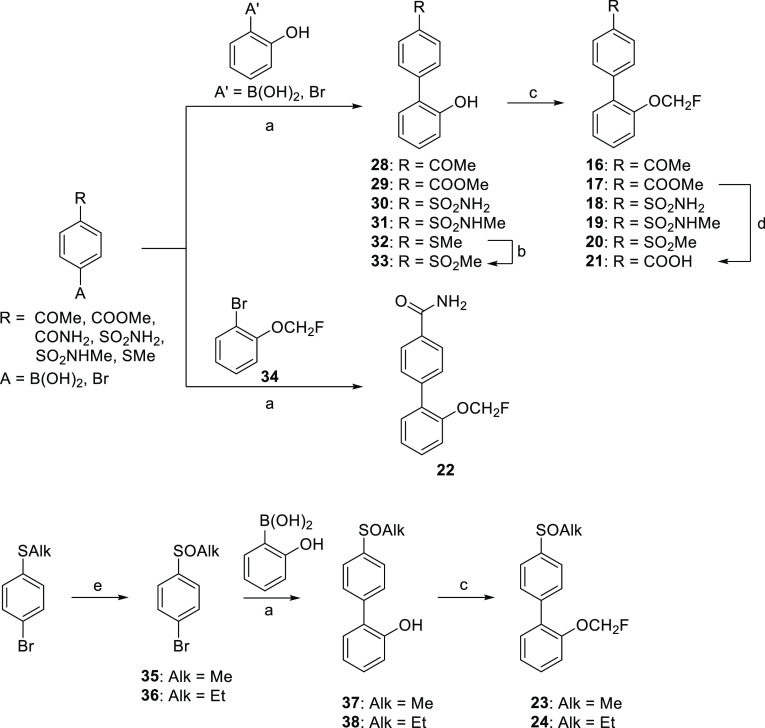
Synthesis of Compounds **16–24** Reagents and conditions: (a) Pd(PPh_3_)_4_, Na_2_CO_3_, toluene/EtOH/H_2_O, MW, 120/170 °C, 10–20 min or Δ, on, 16–99%; (b) H_2_O_2_ (30%), (NH_4_)_6_Mo_7_O_24_·4H_2_O, methanol, 0 °C to rt, 1 h, 75%; (c) ClCH_2_F (2.0 M in DMF), Cs_2_CO_3_, DMF, −78 °C to rt, on, 31–97%; (d) NaOH, THF/H_2_O, rt, 12 h, quantitative; (e) *m*CPBA, DCM, 0 °C to rt, 4 h, 83–92%.

**Scheme 3:**
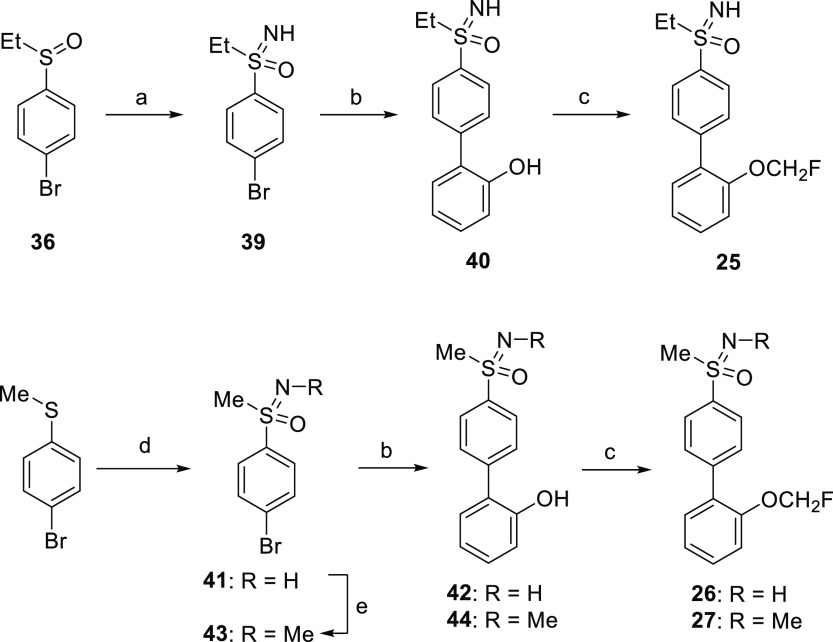
Synthesis of Compounds **25–27** Reagents and conditions: (a) NaN_3_, H_2_SO_4_ (concd), CHCl_3_, 45 °C, on, 92%; (b) (2-hydroxyphenyl)boronic acid, Pd(PPh_3_)_4_, Na_2_CO_3_, toluene/EtOH/H_2_O, MW, 100/120 °C, 20 min, 45–90%; (c) ClCH_2_F (2.0 M in DMF), Cs_2_CO_3_, DMF, −78 °C to rt, on, 78–95%; (d) (i) cyanamide, NBS, *t*BuOK, methanol, rt, 1.5 h, quantitative; (ii) RuCl_3_, NaIO_4_ (aq, 0.15 M), ACN/DCM, rt, 3 h, 83%; (iii) H_2_SO_4_ (aq, 50%), Δ, 2 h, 77%; (e) HCO_2_H, HCHO (aq, 37%), 100 °C, 2 d, quantitative.

A medicinal chemistry program, including 2-fluoromethoxy analogues **16–27**, was conducted for the optimization of compound **3** (Figure 2). The synthesis of target compounds **16–21** was accomplished using a Suzuki–Miyaura coupling followed by monofluoroalkylation (Scheme 2). Thus, the adequate bromobenzene derivative and the corresponding arylboronic acid were coupled according to the previously described conditions, to obtain intermediates **28–32**, which afforded final compounds **16–20** by a reaction with chlorofluoromethane. In the case of derivative **20**, intermediate **32** was oxidized to sulfone **33** before the fluoroalkylation step. Carboxylic acid **21** was readily accessible through basic hydrolysis of methyl ester **17**. Regarding amide **22**, the coupling reaction between (4-carbamoylphenyl)boronic acid and 2-bromophenol failed to give the expected biphenyl derivative. In this case, 2-bromophenol was transformed into fluorinated derivative **34**, which did provide final compound **22** by Suzuki–Miyaura coupling. Sulfoxide analogues **23** and **24** were prepared from 1-bromo-4-(methylsulfanyl)benzene and 1-bromo-4-(ethylsulfanyl)benzene (Scheme 2), respectively. Thus, oxidation to arylsulfoxides **35** and **36** and subsequent coupling with (2-hydroxyphenyl)boronic acid afforded intermediates **37** and **38**, leading to the final target compounds by standard fluoroalkylation. NH-sulfoximines **25** and **26** were prepared through two different strategies (Scheme 3). Ethylsulfoximine derivative **25** was synthesized via classical methodology, starting with an imination reaction with hydrazoic acid of previously synthesized sulfoxide **36** to obtain the corresponding sulfoximine **39**. Next, coupling with (2-hydroxyphenyl)boronic acid under MW standard conditions and reaction with chlorofluoromethane yielded desired compound **25**. For the synthesis of methylsulfoximine **26**, a safer procedure was used for the initial imination step. Thus, the reaction of commercial 1-bromo-4-(methylsulfanyl)benzene with cyanamide and *N*-bromosuccinimide afforded an intermediate *N*-cyanosulfylimine, which was directly transformed into NH-sulfoximine **41** through oxidation and hydrolysis. Then, Suzuki–Miyaura followed by fluoroalkylation yielded final compound **26**. *N*-Methylsulfoximine **27** was obtained using the same Suzuki–Miyaura-fluoroalkylation sequence but starting from *N*-methylated intermediate **43**, which was synthesized by reductive alkylation of **41** employing formaldehyde and formic acid.

**Scheme 4:**
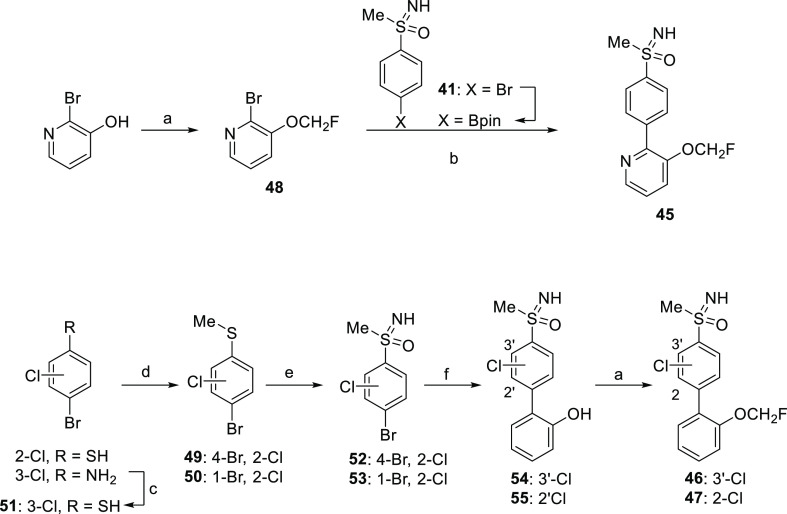
Synthesis of Compounds **45–47** Reagents and conditions: (a) ClCH_2_F (2.0 M in DMF), Cs_2_CO_3_, DMF, −78 °C to rt, on, 73–96%; (b) (i) B_2_pin_2_, Pd_2_(dba)_3_·CHCl_3_, SPhos, KOAc, 1,4-dioxane, 110 °C, on; (ii) Pd_2_(dba)_3_·CHCl_3_, K_3_PO_4_ (aq, 5 M), 110 °C, on, 24%; (c) (i) NaNO_2_, HCl (conc), potassium *O*-ethyl carbonodithioate, −5 to 75 °C, 1.5 h; (ii) KOH, EtOH, Δ, on, 80% (2 steps); (d) MeI, K_2_CO_3_, acetone, rt, 5 h, 56%-quantitative; (e) (i) cyanamide, NBS, KO*t*Bu, methanol, rt, 1.5 h, quantitative; (ii) RuCl_3_, NaIO_4_ (aq, 0.15 M), ACN/DCM, rt, 3 h, 84–89%; (iii) H_2_SO_4_ (aq, 50%), Δ, 2 h, 46–51%; (f) (2-hydroxyphenyl)boronic acid, Pd(PPh_3_)_4_, Na_2_CO_3_, toluene/EtOH/H_2_O, MW, 100/120 °C, 20 min, 77–89%.

The assessment of the new synthesized compounds **16–27** as D_1_R PAMs was carried out in the potentiator-mode cAMP assay (Table 2). Replacement of the aldehyde for other carbonyl-containing moieties (ketone, ester, carboxylic acid, and amide in compounds **16**, **17**, **21**, and **22**, respectively), sulfonamide (**18** and **19**), or sulfonyl (**20**) was detrimental for the activity. On the other hand, a sulfinyl or sulfoximine group seemed tolerable. In the case of sulfinyl derivatives, a marked drop in activity was observed from methyl to ethyl analogues (**23**, 60% vs **24**, 16% at 10 μM), while potentiation was maintained for sulfoximine derivatives (**25**, 54% vs **26**, 55% at 10 μM). Methylation of the nitrogen in the sulfoximine group produced a decrease in activity (**27**, 30% at 10 μM). In this series, sulfoximine analogue **26** was 2 orders of magnitude more potent than parent compound **3** (EC_50_ = 60 nM vs 12.7 μM, Table 2) and exhibited high potentiation of DA *E*_max_ (55% at 10 μM). Hence, we next considered the synthesis of new sulfoximine derivatives **45–47**, which were prepared using an NH-bromoarylsulfoximine as the coupling partner in a Suzuki–Miyaura reaction (Scheme 4). For the synthesis of pyridine derivative **45**, a one-pot borylation-Suzuki–Miyaura coupling strategy was carried out. Thus, NH-sulfoximine **41** was converted to the corresponding pinacol boronate derivative and coupled in situ to intermediate **48**, obtained by fluoroalkylation of 2-bromopyridin-3-ol. Compounds **46** and **47** were synthesized by coupling of bromochloroarylsulfoximines **52** and **53** with (2-hydroxyphenyl)boronic acid followed by fluoroalkylation of the resulting biphenyl derivatives **54** and **55**. Intermediates **52** and **53** were prepared by sequential imination, oxidation, and hydrolysis reactions of the corresponding bromochloroarylsulfides **49** and **50**, according to the previously setup procedure. Sulfides **49** and **50**, in turn, were synthesized by methylation of 4-bromo-2-chlorobenzenethiol and 4-bromo-3-chlorobenzenethiol (**51**), which was obtained from 4-bromo-3-chloroaniline via diazonium salt.

**Table 2:**
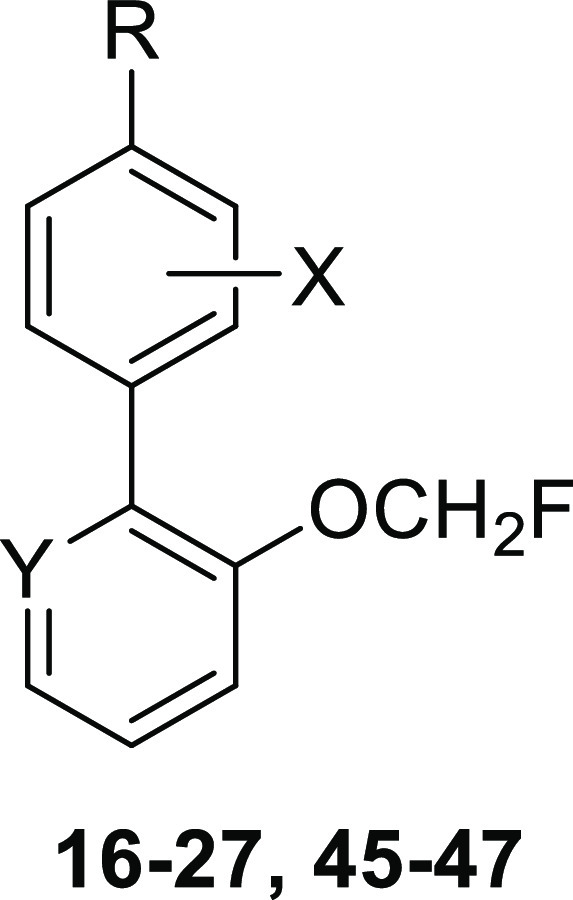
Effect of Compounds **16–27** and **45–47** in DA-induced cAMP in Human D_1_R Endogenously Expressed in a Neuroblastoma Cell Line

compd	R	X	Y	potentiation of DA *E*_max_ (%)^a^	% maximum increase^b^	EC_50_ (μM)^c^
**3**	CHO	H	CH	82 ± 8	91 ± 6	12.7 ± 2.4
**16**	COMe	H	CH	11 ± 3	nd	Nd
**17**	COOMe	H	CH	28 ± 3	nd	Nd
**18**	SO_2_NH_2_	H	CH	31 ± 2	42 ± 7	4.93 ± 1.2
**19**	SO_2_NHMe	H	CH	38 ± 5	28 ± 8	1.26 ± 0.7
**20**	SO_2_Me	H	CH	30 ± 7	30 ± 8	13.11 ± 2.8
**21**	COOH	H	CH	7 ± 3	nd	Nd
**22**	CONH_2_	H	CH	16 ± 4	nd	Nd
**23**	SOMe	H	CH	60 ± 9	73 ± 6	21.7 ± 5.8
**24**	SOEt	H	CH	16 ± 5	nd	Nd
**25**	SO(NH)Et	H	CH	54 ± 7	50 ± 7	30.2 ± 2.4
**26**	SO(NH)Me	H	CH	55 ± 7	45 ± 7	0.06 ± 0.01
**27**	SO(NMe)Me	H	CH	30 ± 5	33 ± 3	15.9 ± 2.7
**45**	SO(NH)Me	H	N	1 ± 2	nd	nd
**46**	SO(NH)Me	3′-Cl	CH	12 ± 4	nd	nd
**47**	SO(NH)Me	2-Cl	CH	20 ± 3	nd	nd

a Effect over the DA concentration–response curve at a fixed concentration of compound = 10 μM.

b Efficacy (measured as % of maximum increase over DA EC_70_) in the concentration–response curves of the compounds over DA EC_70_.

c Potency (measured as EC_50_) of the compounds at DA EC_70_ concentration; values are the mean ± SEM of three independent experiments with duplicate determinations; nd = not determined.

Structural modifications introduced in the new synthesized sulfoximine derivatives **45–47** produced an important depletion of D_1_R allosteric activity, as shown in the data from the potentiator-mode cAMP assay (Table 2). Therefore, compound **26** exhibiting the best allosteric activity (high efficacy and the greatest potency) was selected for further pharmacological characterization and study of ADMET properties.

### In Vitro Pharmacological and Pharmacokinetic Characterization of D_1_R PAM **26**

**Figure 3:**
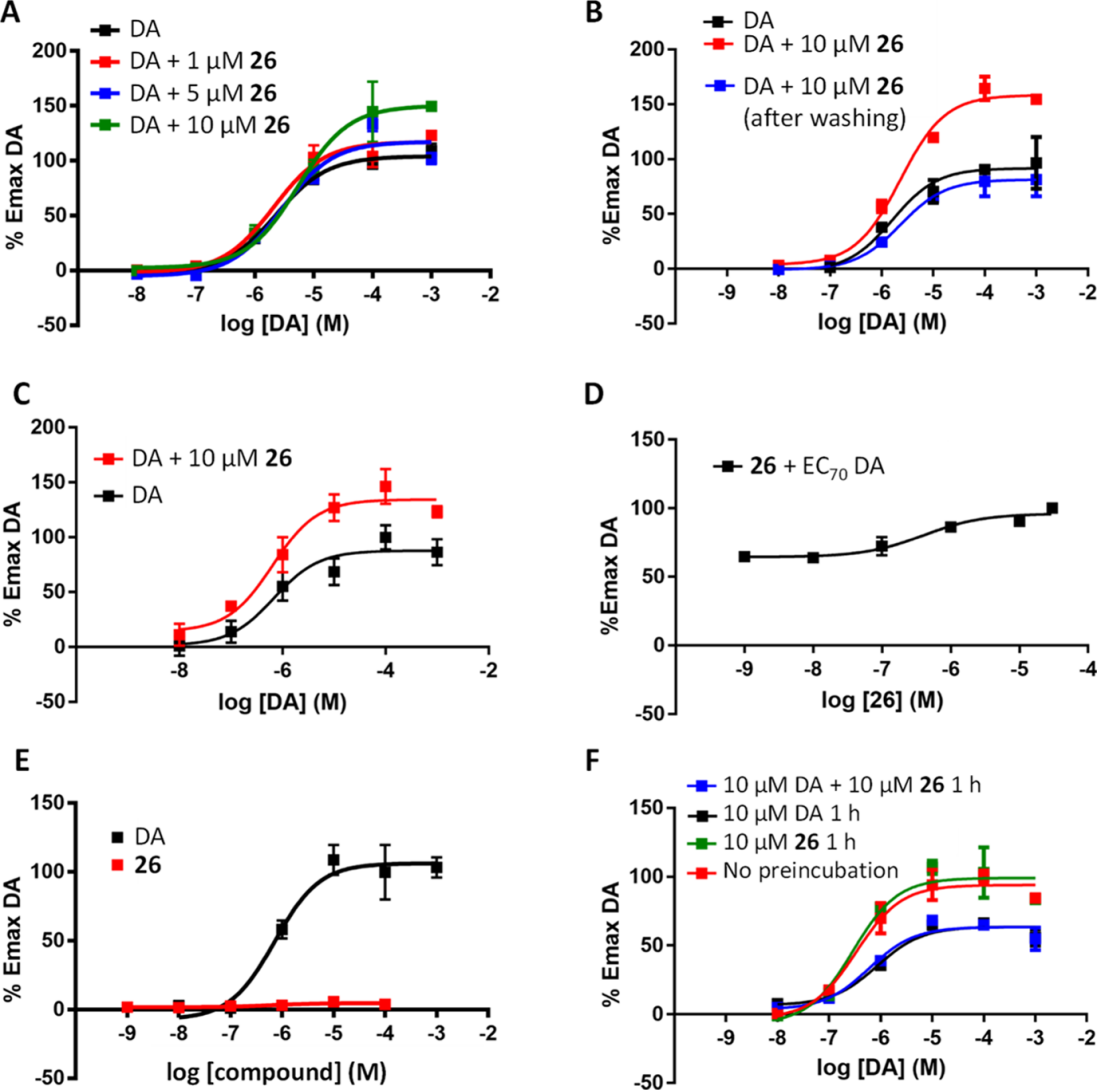
(A) Concentration–response curves in human D_1_R for cAMP production of DA alone (black) and in the presence of different concentrations of compound **26**. (B) Concentration–response curves in human D_1_R of DA (black), 10 μM **26** for 10 min (red), and 10 μM **26** for 10 min then washing with assay buffer (blue). (C) Concentration–response curves in mouse D_1_R of DA alone (black) and in the presence of 10 μM **26** (red). (D) Concentration–response curve in human D_1_R of compound **26** in the presence of DA EC_70_ concentration. (E) Concentration–response curves in human D_1_R of DA (black) and compound **26** (red). (F) Concentration–response curves in human D_1_R of DA (red), after preincubation for 1 h with 10 μM DA (black), with 10 μM **26** (green), and with both 10 μM DA and 10 μM **26** (blue).

Compound **26** was tested in the potentiator-mode cAMP assay at different fixed concentrations (1, 5, and 10 μM). Figure 3A shows a potentiation of the DA *E*_max_ in a concentration-dependent manner, with a maximum value of 55% at 10 μM, and it was demonstrated to be a reversible effect (Figure 3B). A PAM behavior was also observed in mouse D_1_R (Figure 3C). When tested with EC_70_ DA, **26** increased cAMP in a concentration-response manner with high potency (EC_50_ = 60 nM, Figure 3D). Importantly, no cAMP response to compound **26** was determined in the absence of DA, so the new D_1_R PAM does not display agonist activity (Figure 3E). Therefore, it should have negligible direct activating effects on the human D_1_R in the absence of the endogenous ligand. It was also confirmed that the compound does not induce receptor desensitization and it does not induce further increase in DA-induced desensitization (Figure 3F). Hence, efficacy should be retained over time upon repeated dosing of the D_1_R PAM, avoiding the development of tolerance. Compound **26** showed a concentration-dependent increase of the affinity ratio values in the presence of 4 μM DA, evidencing a positive allosteric behavior of this compound over DA effect (Figure S2B). As expected for a PAM, subtype selectivity over human D_2_, D_3_, D_4_, and D_5_ receptors was achieved with compound **26** (Figure S5), whereas selective activation of the D_1_R with agonists has historically been very challenging due to the high sequence homology at the orthosteric site.

In view of the allosteric efficacy of compound **26**, in vitro ADMET properties were determined, and they are summarized in Table 3. In nephelometry assay, a solubility more than 3 times higher than parent compound **3** (50 vs 15 μM, Table S1) was observed. Interaction with HSA proteins revealed 79% binding with moderate dissociation constant (*K*_d_ = 1.56 × 10^–4^ M), indicating a significant reduction compared to **3** (>99% binding, Table S1). PAMPA showed a decent permeability value (*P*) of 24 × 10^–6^ cm/s. Compound **26** displayed a low hERG inhibition (21% at 10 μM). In mouse serum, 93% of the compound remained after 4 h, whereas 50% had been observed for remaining compound **3** (Table S1). To assess first-pass metabolism, compound **26** was incubated in liver homogenates and 65% of remaining compound was detected after 24 h. Hence, the compound displays a good stability profile overall. In a panel of CYP450, low inhibition was observed for CYP2D6 and CYP34A (3 and 9% of inhibition, respectively), while moderate blockade was obtained for CYP1A2, CYP2C9, and CYP2C19 with the percentage of inhibition values in the range of 37–49% (Table 3).

**Table 3:** ADMET Profile for Compound **26**

solubility (μM)^a^	50
HSA binding (%)^b^	79 (*K*_d_ = 1.56 × 10^–4^ M)
*P* (cm/s)^c^	24 × 10^–6^
hERG inhibition (%)^d^	21
mouse serum stability (%)^e^	93
liver homogenate stability (%)^e^	65
CYP2D6 (%)^f^	3 ± 1
CYP34A (%)^f^	9 ± 1
CYP1A2 (%)^f^	49 ± 2
CYP2C9 (%)^f^	38 ± 3
CYP2C19 (%)^f^	37 ± 2

a Maximum solubility measured by nephelometry.

b Binding to human serum albumin (HSA) determined at a concentration of 5 μM.

c Permeability in the parallel artificial membrane permeability assay (PAMPA).

d Blockade of the K^+^ channel current at a concentration of 10 μM.

e Remaining compound quantified after 4 and 24 h, respectively.

f Percentage of inhibition of CYP450 activity measured at a concentration of 10 μM by fluorescence assays.

**Scheme 5:**
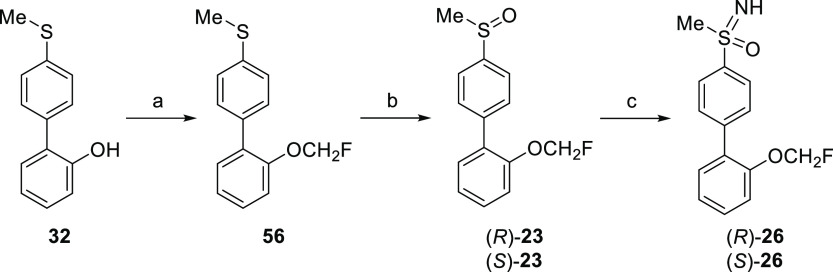
Synthesis of Enantiomers of Compound **26** Reagents and conditions: (a) ClCH_2_F (2.0 M in DMF), Cs_2_CO_3_, DMF, −78 °C to rt, on, 84%; (b) H_2_O_2_ (30%), (*S*)- or (*R*)-2-((*E*)-{[1-(hydroxymethyl)-2,2-dimethylpropyl]imino}methyl)-4,6-diiodophenol, [VO(acac)_2_], CHCl_3_, 0 °C, 20 h, 59–64%, er > 97:3; (c) ammonium carbamate, PhI(OAc)_2_, MeOH, rt, 30–60 min (open flask), 61–67%, er > 97:3.

Altogether, new compound **26** behaves as a potent and efficient D_1_R PAM and exhibits an improved ADMET profile than parent compound **3**. As the sulfoximine group provides chirality to the molecule, it was mandatory to evaluate both enantiomers of compound **26**. Therefore, each enantiomer was prepared separately and tested for allosteric activity at the D_1_R in the potentiator-mode cAMP assay. For the enantioselective synthesis of the sulfoximines, a route in which chirality is introduced through asymmetric oxidation of an intermediate sulfide was used (Scheme 5). Thus, previously synthesized sulfide **32** was transformed into monofluorinated analogue **56** by reaction with chlorofluoromethane. Next, asymmetric oxidation of **56** catalyzed by vanadyl acetylacetonate in the presence of the appropriate chiral *tert*-leucinol derivative,^45,46^ yielded the corresponding sulfoxides (*R*)- and (*S*)-**23** with enantiomeric ratio (er) values higher than 97:3. The reaction of these intermediates with ammonium carbamate and (diacetoxyiodo)benzene provided the highly enantioenriched sulfoximines (*R*)- and (*S*)-**26** (er > 97:3) under very mild conditions^47^ (Scheme 5).

Both enantiomers (*R*)- and (*S*)-**26** exhibited similar pharmacological activity—D_1_R PAM efficacy (42 and 43% potentiation at 10 μM) and no agonist activity (Figure S6)—to that of racemic compound **26**, and the latter was selected as a drug candidate for in vivo validation of therapeutic interest.

### Binding Site for the New D_1_R PAM **26**

**Figure 4:**
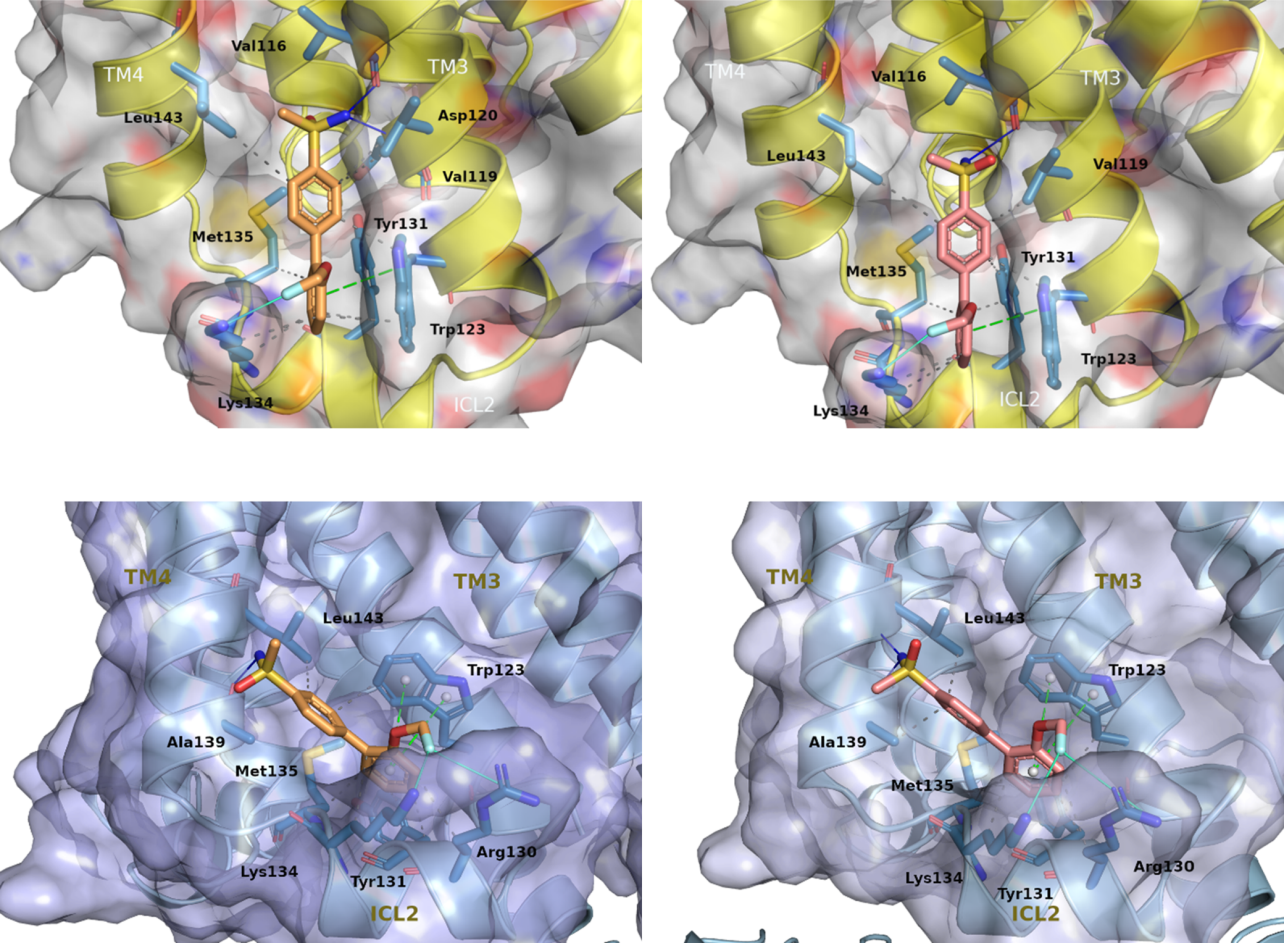
Homology model (top panel, yellow cartoon representation) and electron microscopy model (bottom panel, cyan cartoon representation) of the ICL2 region of human D_1_R bound to (*S*)-**26** (left panel, orange stick representation) and (*R*)-**26** (right panel, pink stick representation) as predicted by docking calculations. The key interactions between the ligand and residues are highlighted: blue lines indicate favorable H-bond interactions, grey dashed lines hydrophobic interactions, turquoise lines halogen bonds, and green dashed lines π–π stacking interactions.

In silico docking calculations were performed to predict the binding mode between compound **26** (*S*- and *R*-stereoisomers) and the active form of human D_1_R. The results in the receptor homology model (Figure 4, top panel) show that both enantiomers bind in a pocket formed by TM3, TM4, and intracellular loop 2 (ICL2), a region previously identified as an allosteric binding site of the D_1_R.^38,48^ Both (*R*)- and (*S*)-**26** bind with an almost equal pose establishing π–π stacking interactions with Trp123 as well as polar interactions with Val116 (H-bond) and Lys134 (halogen bond). Additionally, they form hydrophobic interactions with the following residues: Val119, Trp123, Tyr131, Lys134, Met135, and Leu143. The difference in the binding of the two enantiomers is that (*S*)-**26** forms an additional polar interaction (H-bond) with Asp120 (Figure 4, top left panel) that (*R*)-**26** cannot establish. In order to support the binding poses, short molecular dynamics (MD) simulations were performed, which reveal that the docking poses are quite stable and compounds (*R*)- and (*S*)-**26** remain bound to ICL2. Therefore, the residues interacting with the new compound are similar to those previously proposed for PAMs LY3154207 and DETQ docked in the D_1_R homology model.^38,48^

Very recently, Teng et al. have published an electron microscopy (EM) structure of the D_1_R bound to LY3154207 (PDB code: 7X2F).^49^ In this structure, mostly similar to the homology model used, the most relevant difference is that the conformation of Trp123 is flipped. This causes a shift in the rotation of the LY3154207 pose at the binding site, compared to that previously proposed in the homology model. To understand how this would affect the binding mode of compound **26**, we performed a new docking study using the EM structure. The results in Figure 4 (bottom panel) show that both enantiomers bind in the same region as in the homology model, but adopting a rotated pose forced by the new conformation of Trp123, as in the case of LY3154207. (*R*)- and (*S*)-**26** can establish π–π stacking interactions with Trp123 and polar interactions with Ala139 (H-bond), Arg130 (halogen bond), and Lys134 (halogen bond). They can also form hydrophobic interactions with the following residues: Trp123, Arg130, Tyr131, Lys134, Met135, and Leu143. Both enantiomers bind without major differences, but (*R*)-**26** can establish an additional H-bond with Leu143 and an extra hydrophobic interaction with Ala139 (Figure 4, bottom right panel).

Overall, docking studies in Figure 4—using the homology model or the EM structure of the receptor—reveal that the new PAM **26** interacts with the human D_1_R in a similar way to that reported for other known PAMs of the receptor.^38,48,49^

### In Vivo Efficacy of the New D_1_R PAM 26 in Locomotor Activity Models

**Figure 5:**
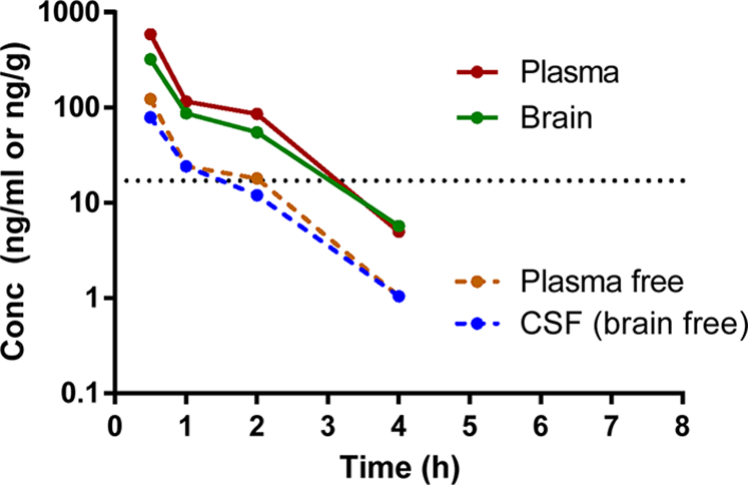
Compound levels in the plasma, brain, and CSF of mouse administered with 5 mg/kg (po) of compound **26**. Horizontal dotted line indicates the EC_50_ value of the compound.

Prior to the evaluation of the new D_1_R PAM **26** in animal models, the in vivo cerebrospinal fluid (CSF) and brain permeability were studied. Following a single oral dose administration of **26** at 5 mg/kg to male BALB/c mice, plasma concentrations were quantifiable up to 8 h with *T*_max_ at 0.5 h (Figure 5). Concentrations in the CSF and brain were quantifiable up to 4 h. Brain-to-plasma concentration ratios were in the range of 0.55–1.15 between 0.5 and 4 h and CSF-to-plasma concentration ratio in the range of 0.13–0.21 between 0.5 and 4 h. In addition, CSF-to-plasma free concentration ratio of approximately 1 indicated that the compound does not show efflux transport from the brain.

**Figure 6:**
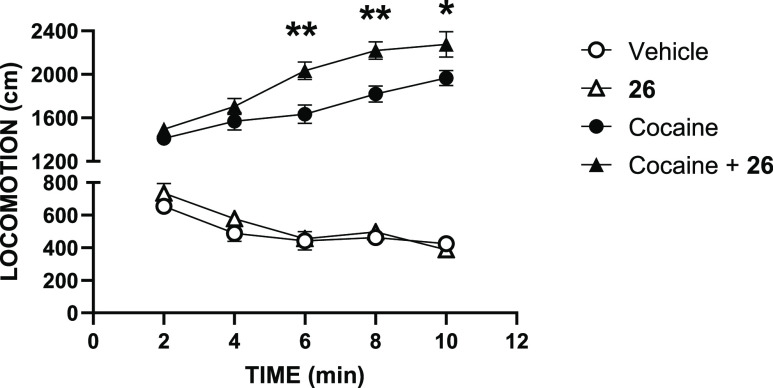
Effect of compound **26** in a cocaine-induced hyperactivity model. Pretreatment with **26** (1 mg/kg, ip) enhanced cocaine (20 mg/kg, sc)-induced hyperlocomotion in adult mice, with no intrinsic locomotor activity when **26** was administered alone [*F*(3.16) = 61.67, *P* < 0.0001, **P < 0.001, *P < 0.01 cocaine vs cocaine + **26** groups, *N* = 8 animals/group].

In view of the good brain penetration and oral availability, compound efficacy in vivo was tested in two different locomotor activity assays: a cocaine-induced hyperactivity model in normal animals and a PD animal model based on the modulation of l-DOPA effect in reserpinized mice. In the first experiment, the administration of compound **26** (1 mg/kg, ip) potentiated the hyperlocomotion induced by cocaine (20 mg/kg, Figure 6). The cocaine-induced increase in extracellular DA concentration results in enhanced locomotion through activation of the D_1_R, which is the dopaminergic receptor responsible for this pharmacological action.^50^ Administration of compound **26** increased this response, suggesting an in vivo potentiation of DA action at the D_1_R.

**Figure 7:**
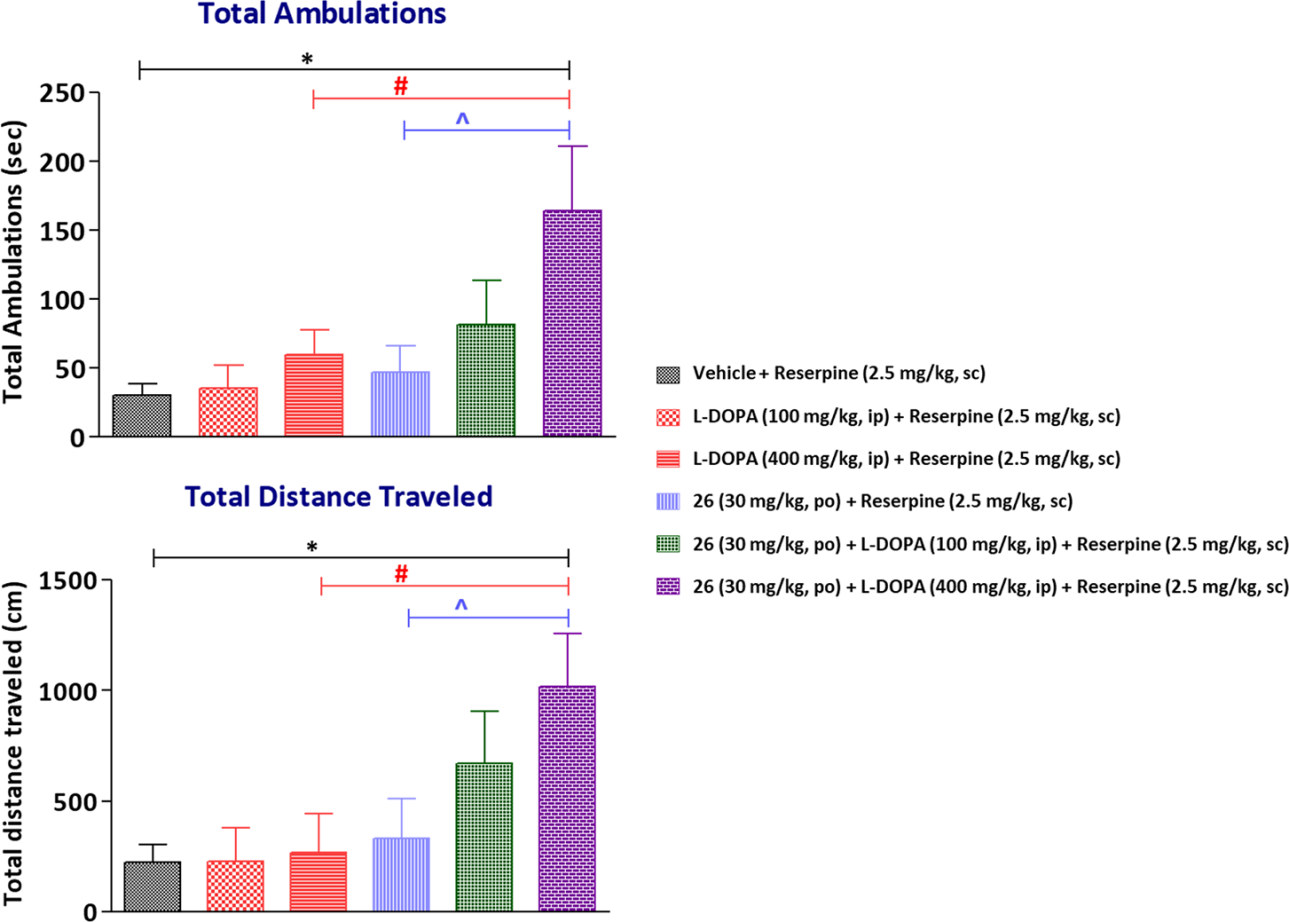
In vivo effect of compound **26** in locomotor activity by measuring ambulatory activity and total distance travelled. Data are shown as the mean ± S.E.M (*N* = 9–10).

Regarding the PD model, reserpine is an alkaloid known to induce hypomobility/akinesia and rigidity, and reserpinized rodents are widely used as a preclinical model for PD. l-DOPA and all clinically active anti-Parkinsonian treatments show efficacy in the reserpine model, exhibiting a reversal of reserpine-induced akinesia. After pretreatment with a low dose of reserpine (2.5 mg/kg, sc), a partial depletion of DA was achieved in mice, as previously described, mimicking the low dopaminergic functionality in PD patients. As shown in Figure 7, reserpinized mice were treated with l-DOPA (100 or 400 mg/kg, ip) and/or PAM **26** (30 mg/kg, po). There was a small recovery of decreased locomotor activity with l-DOPA alone (as described),^3^ a small recovery of decreased locomotor activity with **26** alone (as reported for other D_1_R PAMs),^37,38^ and a moderate synergistic effect with l-DOPA plus compound **26**, as expected for a D_1_R PAM. These results evidenced efficacy of the compound in ambulatory activity of mice with induced low DA levels, similarly to other D_1_R PAMs. For instance, DETQ and LY3154207 have displayed a synergistic effect on locomotion in human D_1_R transgenic mice; the larger effect observed for these PAMs was probably due to the higher level of receptor expression than that in wild-type reserpinized mice used in our model.^51^ The study suggests that the new D_1_R PAM could be therapeutically beneficial to alleviate motor symptoms in PD patients as monotherapy by enhancing the effect of low levels of endogenous DA or the activity of co-administered l-DOPA. In the study, high levels of **26** in both the brain and plasma were confirmed (Table S2) and no sign of adverse effects was observed in the animals after oral dose administration of the compound.

### Effects of Compound **26** on Memory

**Figure 8:**
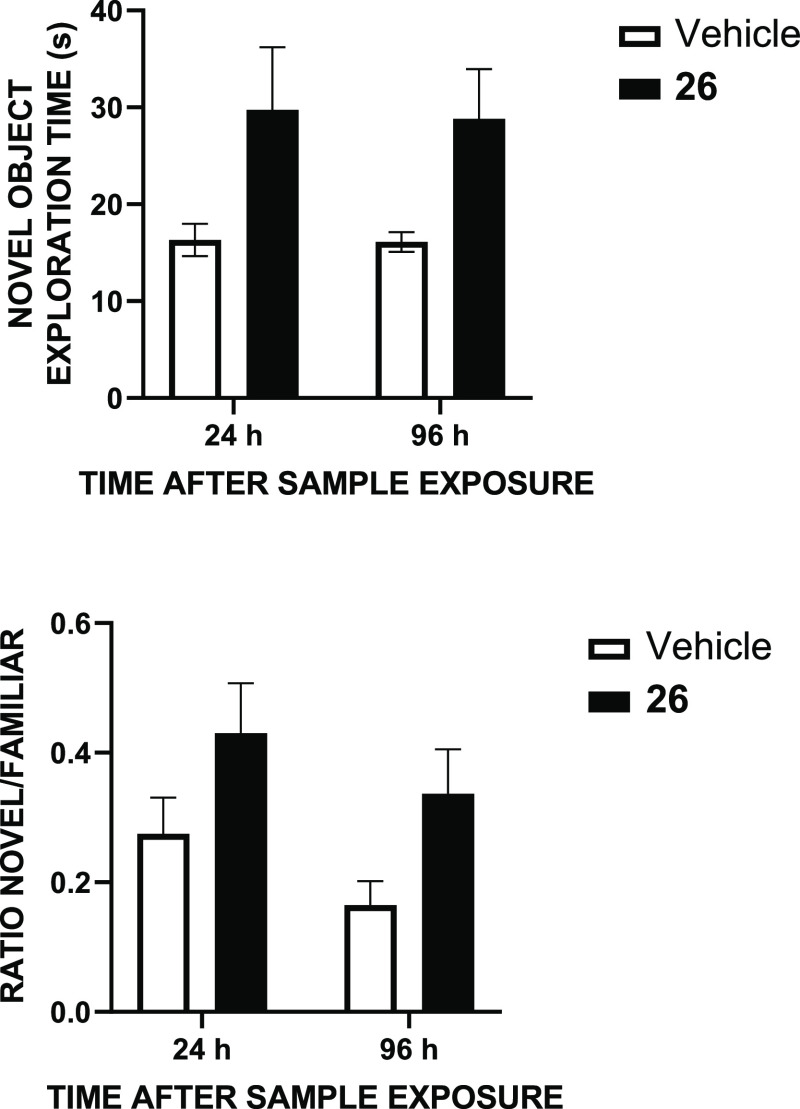
Effect of compound **26** (1 mg/kg, ip) in the novel object recognition test. Enhanced memory, measured as the time spent exploring the novel object [upper panel, *F*(1.28) = 9.4, *p* < 0.005] and the preference over a familiar object [lower panel, *F*(1.28) = 7.1, *p* < 0.02] is observed at 24 and 96 h (2-way ANOVA). *N* = 8 animals/group.

Because PD has been linked to memory deficits and even dementia (alpha-synuclein aggregates that are a major feature of PD are also very prominent in Lewy Body Dementia),^52,53^ we assessed whether compound **26** might have a beneficial effect on memory, by means of the NORT. Data in Figure 8 suggest that the treatment with the compound increases memory trace, even 4 days after its administration and the presentation of the familiar object. This effect suggests that the compound helps consolidate long-term memory formation, probably through the described D_1_R-dependent modification of hippocampal interneurons functionality.^54^ Therefore, the new D_1_R PAM is a good candidate not only for improving motor symptoms but also for addressing the key comorbid cognitive impairment associated with long-term PD.

## Conclusions

In this work, we have developed compound **26** [2-(fluoromethoxy)-4′-(*S*-methanesulfonimidoyl)-1,1′-biphenyl] as an allosteric modulator of the dopaminergic D_1_R that increases the endogenous DA maximal effect in a dose-dependent manner in human and mouse receptors, is inactive in the absence of DA, modulates DA affinity for the receptor, exhibits subtype selectivity, shows a reversible effect, and does not show any desensitization effect avoiding tolerance. Competition assays and docking studies support the binding to an allosteric site of the receptor. The new PAM was found to be orally active in a reserpine-induced low-DA-level model, enhancing l-DOPA recovery of decreased locomotor activity with no sign of adverse effects, which suggests an allosteric modulation of DA effect in vivo. The compound has also memory enhancing effect that might improve cognitive impairment associated to PD. These results support the interest of a D_1_R PAM as a promising therapeutic approach for PD.

## Experimental Section

### Synthesis

Unless stated otherwise, starting materials, reagents, and solvents were purchased as high-grade commercial products from ABCR, Acros, Fluorochem, Scharlab, or Sigma-Aldrich, and were used without further purification. All non-aqueous reactions were performed under an argon atmosphere in oven-dried glassware. Tetrahydrofuran (THF) and dichloromethane (DCM) were dried using a Pure Solv Micro 100 L solvent purification system. Acetone was dried under K_2_CO_3_. Triethylamine was dried over KOH and distilled before use. Reactions under MW irradiation were performed in a Biotage Initiator 2.5 reactor. Reactions were monitored by analytical thin-layer chromatography (TLC) on silica gel plates supplied by Merck (Kieselgel 60 F-254) with detection by UV light (254 nm), 5% ninhydrin solution in ethanol, or 10% phosphomolybdic acid solution in ethanol. Products were purified by flash chromatography using a Varian 971-FP purification system using silica gel cartridges (Varian, particle size 50 μm). All compounds were obtained as oils, except for those whose melting points (mp) are indicated, which were solids. Mp (uncorrected) was determined on a Stuart Scientific electrothermal apparatus. Infrared (IR) spectra were measured on a Bruker Tensor 27 instrument equipped with a Specac ATR accessory of 5200–650 cm^–1^ transmission range; frequencies (ν) are expressed in cm^–1^. Nuclear magnetic resonance (NMR) spectra were recorded on a Bruker AVANCE III 700 MHz (^1^H, 700 MHz; ^13^C, 175 MHz), Bruker AVANCE 500 MHz (^1^H, 500 MHz; ^13^C, 125 MHz), or Bruker DPX 300 MHz (^1^H, 300 MHz; ^13^C, 75 MHz; ^19^F, 300 MHz) instruments at the Universidad Complutense de Madrid (UCM) NMR core facilities. Bruker DPX 300 MHz equipment was used unless otherwise stated. Chemical shifts (δ) are expressed in parts per million relative to the residual solvent peak for ^1^H and ^13^C nucleus (CDCl_3_: δ_H_ = 7.26, δ_C_ = 77.16; DMSO-*d*_6_: δ_H_ = 2.50, δ_C_ = 39.52; acetone-*d*_6_: δ_H_ = 2.05, δ_C_ = 29.84; methanol-*d*_4_: δ_H_ = 3.31, δ_C_ = 49.00) and to internal (trifluoromethyl)benzene for the ^19^F nucleus; coupling constants (*J*) are in hertz (Hz). The following abbreviations are used to describe peak patterns when appropriate: s (singlet), d (doublet), t (triplet), q (quartet), qt (quintet), sext (sextet), m (multiplet), br (broad), and app (apparent). 2D NMR experiments (H,H-COSY, HMQC, and HMBC) of representative compounds were carried out to assign protons and carbons of the new structures. Numbered chemical structures for NMR assignation of final compounds **3**, **8**, **10**, (*R*)- and (*S*)-**23**, (*R*)- and (*S*)-**26** are shown in Figure S7.

For all final compounds, purity was determined by high-performance liquid chromatography (HPLC) coupled to mass spectrometry (MS) using an Agilent 1200LC-MSD VL instrument, and satisfactory chromatograms confirmed a purity of at least 95% for all tested compounds. LC separation was achieved with a Zorbax Eclipse XDB-C18 column (5 μm, 4.6 mm × 150 mm) or a Zorbax SB-C3 column (5 μm, 2.1 mm × 50 mm), both together with a guard column (5 μm, 4.6 mm × 12.5 mm). The gradient mobile phases consisted of A (95:5 water/acetonitrile) and B (5:95 water/acetonitrile) with 0.1% ammonium hydroxide and 0.1% formic acid as the solvent modifiers, and the gradients are indicated in Table S3. MS analysis was performed with an ESI source. The capillary voltage was set to 3.0 kV and the fragmentor voltage was set at 72 eV. The drying gas temperature was 350 °C, the drying gas flow was 10 L/min, and the nebulizer pressure was 20 psi. Spectra were acquired in positive or negative ionization modes from 100 to 1200 *m*/*z* and in the UV-mode at four different wavelengths (210, 230, 254, and 280 nm).

Optical rotation [α] was measured on an Anton Paar MCP 100 modular circular polarimeter using a sodium lamp (λ = 589 nm) with a 1 dm path length; concentrations (c) are given as g/100 mL. Enantiomeric ratios (er) were determined by HPLC using a Daicel Chiralpak IC column (5 μm, 4.6 mm × 150 mm), together with a guard column (5 μm, 4.6 mm × 12.5 mm). The gradient mobile phase consisted of a 1:9 mixture of water/methanol, using a flow of 0.9 mL/min for the injection of the sample and 0.5 mL/min during the run. HPLC traces were compared to racemic samples of sulfoxide **23** and sulfoximine **26**, which were obtained in the absence of any chiral catalysts.

The following compounds were synthesized as previously described and their spectroscopic data correspond with those reported: 1-bromo-4-(methanesulfinyl)benzene (**35**),^55^ 4-bromo-3-chlorobenzenethiol (**51**),^56^ and (*R*)- and (*S*)-2-((*E*)-{[1-(hydroxymethyl)-2,2-dimethylpropyl]imino}methyl)-4,6-diiodophenol.^45^

### General Procedure for Suzuki–Miyaura Reaction

A solution of the corresponding bromoaryl derivative (1.00 equiv), arylboronic acid (1.05–1.20 equiv), and Na_2_CO_3_ (2 or 4 equiv) in a 2:1.5:1 mixture of toluene/water/ethanol or a 1:1 mixture of THF/water (6.5 mL/mmol) was degassed by bubbling argon for 10 min. Pd(PPh_3_)_4_ (0.06 equiv) was then added and the reaction was refluxed overnight or heated under MW irradiation. After this time, the mixture was extracted with EtOAc (×3) and the organic phase was dried over Na_2_SO_4_, filtered, and evaporated under reduced pressure. The residue was purified by recrystallization, column chromatography, or preparative TLC to afford intermediates **11–13**, **28–32**, **37**, **38**, **40**, **42**, **44**, **54**, and **55** or final compounds **5–7**, **9**, **10**, and **22**.

#### 2′-[(Difluoromethyl)sulfanyl][1,1′-biphenyl]-4-carbaldehyde (**10**)

Following the general procedure for the Suzuki–Miyaura reaction, compound **10** was obtained from **15** (104 mg, 0.433 mmol), (4-formylphenyl)boronic acid (70 mg, 0.455 mmol), and Na_2_CO_3_ (92 mg, 0.866 mmol) in a toluene/water/ethanol mixture by heating at 130 °C under MW irradiation for 20 min, as a solid (92 mg, 81%). Chromatography: hexane.

mp 46–47 °C. *R*_f_: 0.46 (hexane/EtOAc 9:1). IR (ATR): ν 1702 (CHO), 1065, 1034 (C–F). ^1^H NMR (CDCl_3_): δ 6.66 (t, *J* = 56.6, 1H, CHF_2_), 7.39–7.48 (m, 3H, H_3′_, H_4′_, H_5′_), 7.52 (d, *J* = 8.1, 2H, H_2_, H_6_), 7.75 (dd, *J* = 7.5, 1.5, 1H, H_6′_), 7.95 (d, *J* = 8.2, 2H, H_3_, H_5_), 10.09 (s, 1H, CHO). ^13^C NMR (CDCl_3_): δ 120.7 (t, *J* = 274.1, CHF_2_), 124.8 (t, *J* = 2.6, C_1′_), 129.1 (C_3′_), 129.5 (C_3_, C_5_), 130.0 (C_5′_), 130.6 (C_2_, C_6_), 130.9 (C_4′_), 135.5 (C_4_), 136.4 (C_6′_), 145.7 (C_2′_), 146.6 (C_1_), 192.0 (CHO). ^19^F NMR (CDCl_3_): δ −91.6. HPLC (Gradient-I, column C3, *t*_R_, min): 12.27. MS (ESI, *m*/*z*, %): 265.0 ([M + H]^+^, 100).

### General Procedure for the Monofluoroalkylation Reaction

To a mixture of the corresponding hydroxy- or sulfanylaryl derivative (1.00 equiv) and Cs_2_CO_3_ (1.60 equiv) in anhydrous DMF (7.0 mL/mmol) at −78 °C, a solution of chlorofluoromethane (2.0 M in DMF, 4.00 equiv) was added dropwise. The reaction was stirred and allowed to warm to rt overnight. Afterward, the mixture was diluted with water and extracted with Et_2_O (×3). The combined organic layers were dried over Na_2_SO_4_, filtered, and concentrated under reduced pressure. The residue was purified by column chromatography or preparative TLC to afford intermediates **14**, **34**, **48**, and **56** or final compounds **1–4**, **16–20**, **23–27**, **46**, and **47**.

#### 2′-(Fluoromethoxy)[1,1′-biphenyl]-4-carbaldehyde (**3**)

Following the general procedure for the monofluoroalkylation reaction, compound **3** was obtained from **12** (1.01 g, 5.10 mmol) as oil (0.94 g, 84%). Chromatography: hexane.

*R*_f_: 0.39 (hexane/DCM 7:3). IR (ATR): ν 1699 (CHO), 1215 (C–O–C), 1129, 1082 (C–F). ^1^H NMR (CDCl_3_): δ 5.68 (d, *J* = 54.3, 2H, CH_2_F), 7.19–7.28 (m, 2H, H_3′_, H_5′_), 7.37–7.44 (m, 2H, H_4′_, H_6′_), 7.68 (d, *J* = 8.2, 2H, H_2_, H_6_), 7.94 (d, *J* = 8.4, 2H, H_3_, H_5_), 10.06 (s, 1H, CHO). ^13^C NMR (CDCl_3_): δ 100.9 (d, *J* = 218.6, CH_2_F), 116.0 (d, *J* = 1.4, C_3′_), 124.1 (C_5′_), 129.6 (C_3_, C_5_), 130.0 (C_4′_), 130.4 (C_2_, C_6_), 130.8 (C_1′_), 131.1 (C_6′_), 135.2 (C_4_), 144.3 (C_1_), 153.7 (d, *J* = 3.1, C_2′_), 192.1 (CHO). ^19^F NMR (CDCl_3_): δ −150.9. HPLC (Gradient-I, column C3, *t*_R_, min): 11.42. MS (ESI, *m*/*z*, %): 231.1 ([M + H]^+^, 100).

#### 2-(Fluoromethoxy)-4′-(*S*-methanesulfonimidoyl)-1,1′-biphenyl (**26**)

Following the general procedure for the monofluoroalkylation reaction, compound **26** was obtained from **42** (26 mg, 0.105 mmol) as a solid (23 mg, 78%). Chromatography: preparative TLC in toluene/methanol 95:5.

mp 144–145 °C. *R*_f_: 0.40 (toluene/methanol 9:1). IR (ATR): ν 3341 (NH), 1220 (C–O–C), 1129, 1083 (SO), 994, 972 (C–F). ^1^H NMR (acetone-*d*_6_, 500 MHz): δ 3.10 (s, 3H, CH_3_), 5.84 (d, *J* = 54.4, 2H, CH_2_F), 7.26 (td, *J* = 7.5, 1.0, 1H, H_5_), 7.35 (d, *J* = 8.3, 1H, H_3_), 7.44–7.49 (m, 2H, H_4_, H_6_), 7.73 (d, *J* = 8.5, 2H, H_2′_, H_6′_), 8.04 (d, *J* = 8.5, 2H, H_3′_, H_5′_). ^13^C NMR (acetone-*d*_6_): δ 46.6 (CH_3_), 101.8 (d, *J* = 216.8, CH_2_F), 116.4 (d, *J* = 1.1, C_3_), 124.7 (C_5_), 128.3 (C_3′_, C_5′_), 130.8 (C_4_), 130.9 (C_2′_, C_6′_), 131.2 (d, *J* = 1.1, C_1_), 131.9 (C_6_), 143.1 (C_1′_), 144.2 (C_4′_), 154.5 (d, *J* = 3.0, C_2_). ^19^F NMR (acetone-*d*_6_): δ −151.0. HPLC (gradient-I, column C18, *t*_R_, min): 13.17. MS (ESI, *m*/*z*, %): 279.8 ([M + H]^+^, 100).

### General Procedure for the Difluoroalkylation Reaction

Diethyl [bromo(difluoro)methyl]phosphonate (2.00 equiv) was added in one portion to a −78 °C cooled solution of the adequate hydroxy- or sulfanylaryl derivative (1.00 equiv) and KOH (20 equiv) in a 1:1 mixture of ACN/water (10 mL/mmol), and the reaction was stirred and allowed to warm to rt overnight. Next, the mixture was diluted with water and the aqueous layer was extracted with Et_2_O (×3). The combined organic layers were dried over Na_2_SO_4_, filtered, and concentrated under reduced pressure. The residue was purified by column chromatography or preparative TLC to afford intermediate **15** or final compound **8**.

#### 2′-(Difluoromethoxy)[1,1′-biphenyl]-3-carbaldehyde (**8**)

Following the general procedure for the difluoroalkylation reaction, compound **8** was obtained from **13** (19 mg, 0.096 mmol) as a solid (10 mg, 40%). Chromatography: preparative TLC in 98:2 toluene/methanol.

*R*_f_: 0.34 (toluene/methanol 98:2). IR (ATR): ν 1703 (CHO), 1217 (C–O–C), 1138, 1052 (C–F). ^1^H NMR (CDCl_3_): δ 6.39 (t, *J* = 73.7, 1H, CHF_2_), 7.29–7.35 (m, 1H, H_5′_), 7.35 (dd, *J* = 7.4, 1.4, 1H, H_3′_), 7.39–7.47 (m, 2H, H_4′_, H_6′_), 7.63 (t, *J* = 7.6, 1H, H_5_), 7.79 (dt, *J* = 7.8, 1.5, 1H, H_6_), 7.91 (dt, *J* = 7.6, 1.4, 1H, H_4_), 8.00 (m, 1H, H_2_), 10.08 (s, 1H, CHO). ^13^C NMR (CDCl_3_, 125 MHz): δ 116.1 (t, *J* = 258.9, CHF_2_), 120.2 (C_3′_), 126.1 (C_5′_), 128.9 (C_4_), 129.1 (C_6′_), 129.6 (C_5_), 130.9 (C_2_), 131.5 (C_4′_), 132.8 (C_1′_), 135.6 (C_6_), 136.7 (C_3_), 138.2 (C_1_), 148.2 (t, *J* = 2.5, C_2′_), 192.3 (CHO). ^19^F NMR (CDCl_3_): δ −80.9. HPLC (Gradient-I, column C3, *t*_R_, min): 11.53. MS (ESI, *m*/*z*, %): 271.1 ([M + Na]^+^, 100).

### Synthesis of (*R*)- and (*S*)-**26**

#### (*R*)- and (*S*)-2-(Fluoromethoxy)-4′-(methanesulfinyl)-1,1′-biphenyl [(*R*)- and (*S*)-**23**]

A solution of VO(acac)_2_ (0.010 equiv) in anhydrous CHCl_3_ (0.25 mL/mmol sulfide) was added to a solution of the appropriate *R* or *S* form of 2-((*E*)-{[1-(hydroxymethyl)-2,2-dimethylpropyl]imino}methyl)-4,6-diiodophenol (0.015 equiv) in anhydrous CHCl_3_ (0.25 mL/mmol sulfide) and the mixture was stirred at rt for 2 h. Next, a solution of **56** (1.00 equiv) in anhydrous CHCl_3_ (0.50 mL/mmol) was added and the reaction was stirred at rt for 30 min before cooling it to 0 °C (ice bath). After 30 min, hydrogen peroxide (30%, 1.20 equiv) was added and the mixture was stirred vigorously at 0 °C for 20 h. Then, the reaction was quenched with 10% aqueous solution of Na_2_S_2_O_3_ (3.30 mL/mmol sulfide) and extracted with DCM (×3). The combined organic layers were dried over Na_2_SO_4_, filtered, and the solvent was evaporated under reduced pressure. The crude residue was purified by column chromatography to afford the corresponding enantioenriched sulfoxide **23**.

(*R*)-**23**. Following the previous procedure, compound (*R*)-**23** was obtained from **56** (318 mg, 1.28 mmol) using chiral ligand (*R*)-2-((*E*)-{[1-(hydroxymethyl)-2,2-dimethylpropyl]imino}methyl)-4,6-diiodophenol (9.1 mg, 0.020 mmol), as oil (198 mg, 59%, er = 97.9:2.1). Chromatography: hexane to hexane/EtOAc 3:7. [α]_D_^20^ 81.2 (*c* 0.24, acetone). Chiral HPLC (*t*_R_, min): 17.31. Spectroscopic data were in agreement with those described for racemate **23**.

(*S*)-**23**. Following the previous procedure, compound (*S*)-**23** was obtained from **56** (152 mg, 0.610 mmol) using chiral ligand (*S*)-2-((*E*)-{[1-(hydroxymethyl)-2,2-dimethylpropyl]imino}methyl)-4,6-diiodophenol (4.3 mg, 0.010 mmol), as oil (103 mg, 64%, er = 97.9:2.1). Chromatography: hexane to hexane/EtOAc 3:7. [α]_D_^20^ −80.3 (*c* 0.24, acetone). Chiral HPLC (*t*_R_, min): 15.66. Spectroscopic data were in agreement with those described for racemate **23**.

#### (*R*)- and (*S*)-2-(Fluoromethoxy)-4′-(*S*-methanesulfonimidoyl)-1,1′-biphenyl [(*R*)- and (*S*)-**26**]

The adequate sulfoxide (*R*)- or (*S*)-**23** (1.00 equiv), ammonium carbamate (4.00 equiv), and (diacetoxyiodo)benzene (3.00 equiv) were dissolved in methanol (2.0 mL/mmol) and the mixture was stirred at rt for 30–60 min (TLC) in an open flask. After completion, the solvent was evaporated under reduced pressure and the residue was purified by column chromatography to afford the corresponding enantioenriched sulfoximine **26**.

(*R*)-**26**. Following the previous procedure, compound (*R*)-**26** was obtained from (*R*)-**23** (335 mg, 1.27 mmol) as a white solid (216 mg, 61%, er = 97.9:2.1). Chromatography: hexane to hexane/EtOAc 7:3. mp 95–96 °C. [α]_D_^20^ −21.5 (*c* 0.20, acetone). Chiral HPLC (*t*_R_, min): 17.17. Spectroscopic data were in agreement with those described for racemic compound **26**.

(*S*)-**26**. Following the previous procedure, compound (*S*)-**26** was obtained from (*S*)-**23** (282 mg, 1.07 mmol) as a white solid (200 mg, 67%, er = 97.3:2.7). Chromatography: hexane to hexane/EtOAc 7:3. [α]_D_^20^ 20.8 (*c* 0.20, acetone). Chiral HPLC (*t*_R_, min): 14.60. Spectroscopic data were in agreement with those described for racemic compound **26**.

### Pharmacology

#### Functional Activity at Human D_1_R and D_2_R

Functional studies at human D_1_ and D_2_ receptors were carried out in endogenously expressed human D_1_R in the SK-N-MC cell line (ATCC), and recombinant human D_2_R stably transfected in an in-house CHO cell line. Cells were thawed and seeded into a black 96-well plate (1 × 10^4^ cells/well for SK-N-MC cell line, 5 × 10^3^ cells/well for CHO cell line) in Opti-MEM containing 500 μM IBMX (3-isobutyl-1-methylxanthine). Test compounds were added to the cells and incubated for 15 min at 25 °C for D_1_R, and for 5 min at 37 °C for D_2_R. After this time, dopamine was added to the corresponding wells and incubated for additional 15 min at 25 °C for D_1_R, and 10 min at 37 °C for D_2_R. Next, treated cells were lysed (for the D_2_R assay, cells were previously treated with 10 μM forskolin for 5 min at 37 °C) and the cAMP concentration was measured by HTRF, using a kit from Cisbio. HTRF was read in a Tecan M1000 Genius Pro reader (λ_excitation_ = 320 nm, λ_emission_ = 620 and 665 nm, 30 flashes), and data were normalized to the dopamine maximum effect and fitted to a 4-parameter logistic equation by using Prism v2.1 software (GraphPad Inc).

#### Functional Activity at Human D_3_R and D_4_R

Functional studies at both human D_3_ and D_4_ receptors were carried out in human D_3_ and D_4_ receptors, using a PatHunter Beta-arrestin eXpress GPCR assay kit from DiscoverX. Briefly, cells were plated and maintained for 48 h at 37 °C in a 5% CO_2_ atmosphere in cell plating reagent provided in the kit. After this time, test compounds were added to the cells and incubated for 30 min at 37 °C. Dopamine was added to the cells and incubated for an additional 90 min at 37 °C. Cells were then treated with the working detection solution provided with the kit for 1 h and beta-arrestin translocation was measured by luminescence detection (integration time = 500 ms) in a Tecan M1000 Genius Pro reader. Data were normalized to dopamine maximum effect and fitted to a 4-parameter logistic equation using Prism v2.1 software (GraphPad Inc).

#### Functional Activity at Human D_5_R

Functional studies at human D_5_R receptors were carried out in human D_5_R, using a cAMP Hunter eXpress DRD5 assay kit from DiscoverX. Briefly, cells were plated and maintained for 24 h at 37 °C in a 5% CO_2_ atmosphere in the cell plating reagent provided in the kit. After this time, test compounds were added to the cells and incubated for 15 min at 37 °C, followed by treatment with dopamine and incubation for an additional 30 min at 37 °C. Cells were then treated with the cAMP working detection solution provided with the kit for 1 h, and then cAMP solution A was added and incubated for 3 h at rt. cAMP formation was measured by luminescence detection (integration time = 500 ms) in a Tecan M1000 Genius Pro reader. Data were normalized to dopamine maximum effect and fitted to a 4-parameter logistic equation using Prism v2.1 software (GraphPad Inc).

#### Radioligand Binding Assays at Human D_1_R

Binding assays were carried out by using plasma membranes (12 μg/well) expressing human dopamine D_1_R (Perkin Elmer). Membranes were incubated in a Multiscreen FC 96-well plate (Millipore) with 0.7 nM [^3^H]SCH23390 (Perkin Elmer) and increasing concentrations of the test compound in assay buffer (50 mM Tris–HCl, 5 mM MgCl_2_; pH 7.4) for 1 h at 27 °C. After this time, the well content was filtered through a Millipore manifold and membranes were washed four times with assay buffer. The plate was dried and radioactivity was measured in a Microbeta Trilux liquid scintillation reader (Perkin Elmer). Non-specific binding was determined in the presence of 1 μM (+)-butaclamol. Data were normalized to the percentage of specific binding and fitted to a 4-parameter logistic equation using with Prism v2.1 software (GraphPad Inc). For the calculation of the affinity ratio of DA in the presence of the allosteric modulators, data were fitted according to the equations reported by Lazareno and Birdsall.^44^

#### Cocaine-induced Hyperlocomotion Assay

The experiment was conducted in adherence to the European Communities Council Directive (86/609/ECC) and Spanish regulations (BOE 252/34367-91, 2005) for the use of laboratory animals. The assay was performed according to a previously reported method^57^ using 3 month old male mice with C57BL/6J background, and 8 mice were used for each experimental condition. All mice were handled and habituated to the injection procedures once per day for 5 days prior to behavioral testing. Experiments were carried out between 8:00 and 20:00, and the animals were acclimated to the experimental room for 30 min each day. Performance in the open field was recorded by a computer-based video tracking system (Smart v2.5, Panlab, Barcelona, Spain). Four open fields (50 × 50 × 50 cm, Panlab) with gray backgrounds were used, and the maximum light intensity in the center of the open field was 100 lux. Animals received a dose of **26** (1 mg/kg, ip) in a volume of 20 mL/kg of sterile saline as the vehicle, 15 min prior to a dose of cocaine (20 mg/kg, sc). Five min after cocaine injection, animals were placed in the open field and locomotion was measured at 5 blocks of 2 min along a 30-min period of analysis. Horizontal locomotion was measured as total distance traveled (cm).

#### Locomotor Activity in Reserpinized Mice

These studies were conducted in SAI Life Sciences Ltd. and all procedures were in accordance with the guidelines provided by the Committee for the Purpose of Control and Supervision of Experiments on Animals (CPCSEA) as published in The Gazette of India, December 15, 1998. The study procedures and husbandry care of the study animals were performed in compliance with AAALAC (Unit no. 001384), OLAW (PHS Assurance No.A5937-01) and CPCSEA (Reg. no. 1240/PO/RcBi/S/08/CPCSEA) norms.

The study was carried out in male C57BL/6 wild-type (22–25 g) mice that were acclimatized for 5 days. Animals were randomized based on body weight into six groups (G1 to G6), ten animals per group (*n* = 10), being group G1 the naïve control group. Animals were fed with standard diet ad libitum. Animals were dosed with subcutaneous administration of reserpine (2.5 mg/kg in DMSO/saline) and after 20 h they were treated with a vehicle (10 mL/kg, po), compound **26** (30 mg/kg, po), l-DOPA (100 or 400 mg/kg, ip) + carbidopa (100 mg/kg, ip) and/or their combination, as indicated in Figure 7. The vehicle employed for compound **26** administration was 5% NMP + 5% solutol HS-15 + 90% normal saline. l-DOPA and carbidopa were administered 30 min prior to start of locomotor activity recording. Compound **26** was administered and 15 min later, mice were placed in the locomotor activity cage to measure the locomotor activity for 60 min (total distance travelled) using ALL Maze version 0.5 automated software enabled with video tracking system. After the activity sessions, raw data were reduced using ALL Maze 5.0 software and Microsoft Excel. Statistical analysis was done with GraphPad Prism-5 using one way ANOVA followed by Dunnett’s test/Tukey’s post hoc test and/or two-way ANOVA followed by Bonferroni post hoc test. Data were considered statistically significant, if P value was less than 0.05.

#### Novel Object Recognition Test

Procedures for animal experiments were conducted in adherence to the European Communities Council Directive (86/609/ECC) and Spanish regulations (BOE 252/34367-91, 2005) for the use of laboratory animals. Experiments were performed on 3 month old male mice with a C57BL/6J background. Eight mice were used for each experimental condition. Mice were maintained on a 12 h light/dark cycle (lights on at 08:00 am), with water and food provided ad libitum. Object recognition test was performed according to a previously reported method.^58^ Briefly, mice were first habituated to an empty open field apparatus (40 × 40 × 40 cm) for 5 min. After the habituation session, mice received an ip injection of compound **26** (1 mg/kg) or vehicle (NaCl 0.9%). Twenty minutes later, mice were placed again in the open field, which now included two identical copies of an object (“familiar” object) located in two adjacent corners, and mice were allowed to explore for 10 min (acquisition session). In the first test session (24 h after the acquisition session), the objects were replaced by a copy of the familiar object and an unknown object (“new object 1”) located in the previous positions. Finally, in the second test session (96 h after the acquisition session), the objects were replaced by another copy of the familiar object and a new unknown object (“new object 2”). Mice were allowed to explore for 10 min in each test session. The total time of object exploration (defined as the mouse touching an object with its nose or forepaws) was scored, and object recognition memory was calculated by the following discrimination ratio: time exploring the new object—time exploring the familiar object)/total time exploring both objects.

#### Docking Studies

Docking calculations were performed using Autodock4^59^ (using ga_num_evals = 2512000, ga_run = 100 and all the other parameters set to their default values). The D_1_R active state homology model was retrieved from a previously reported^38^ while the EM structure was retrieved from Protein Data Bank (PDB code 7X2F).^49,60^ Both structures were prepared for docking using pdb2pqr^61,62^ with propka^63,64^ protonation option at pH 7.4 and the peoepb force field.^65^ (*R*)- and (*S*)-**26** were modeled using RDKIT (Open-source cheminformatics) and their protonation state were adjusted at pH 7.4 by ChemAxon cxcalc module (command line version of ChemAxon’s Calculator Plugins, v16.10.24.0, 2016). Binding mode pictures were created using PyMOL v1.8 and PLIP v1.4.4.

Short MD simulations were performed using Gromacs v201814.^66^ Previous to any simulation, the protein–ligand complex was embedded into a 128 POPC pre-equilibrated membrane. The Amber14SB,^67^ the GAFF^68^ and the Lipid14^69^ force-field set of parameters were employed for the D_1_R active state model, the docking poses and the POPC (phosphatidylcholine) membrane, respectively. Simulations were carried out in explicit solvent using the SPC (simple point-charge) water model^70^ with the imposition of periodic boundary conditions via a cubic box, at 0.15 M (NaCl). The temperature was maintained at 300 K using a V-rescale thermostat^71^ and the pressure was maintained at 1 atm using a Berendsen barostat.^70^ All bonds involving hydrogen atoms were constrained using the LINCS algorithm.^72,73^ Before the production runs, the structure was energy minimized followed by a *NVT* (number of particles, volume, and temperature) equilibration, a slow heating-up phase (at constant temperature and pressure) and a *NPT* (number of particles, pressure, and temperature) equilibration, using harmonic position restraints on the heavy atoms of the protein. Then, the production run was performed without position restraints.

## Supporting Information Available

Characterization data of compounds, experimental procedures for ADMET assays, NMR spectra, HPLC analysis, ADMET profile, plasma and brain concentration, concentration–response curves, selectivity profile, radioligand competition assays, and pharmacological characterization (PDF)
Molecular formula strings (XLSX)
Homology model for compound (*S*)-**26** (PDB)
Homology model for compound (*R*)-**26** (PDB)
EM model for compound (*S*)-**26** (PDB)
EM model for compound (*R*)-**26** (PDB)

## Supplementary Material

jm2c00949_si_002.xlsx

jm2c00949_si_003.pdb

jm2c00949_si_004.pdb

jm2c00949_si_005.pdb

jm2c00949_si_006.pdb

## Abbreviations

ACN: acetonitrile
app: apparent
br: broad
D_1–5_R: D_1–5_ receptors
EC_70_: concentration of dopamine that induces a 70% of the maximum effect
*E*_max_: maximal receptor activation
EM: electron microscopy
HTRF: homogeneous time-resolved fluorescence energy transfer
ICL2: intracellular loop 2
MW: microwave
nd: not determined
*P*: permeability value
SAM: silent allosteric modulator
SEM: standard error of the mean
SMD: steered molecular dynamics
*T*_max_: time to achieve peak plasma concentration.
