# Supplementary material for: 2-(Fluoromethoxy)-4′-(S-methanesulfonimidoyl)-1,1′-biphenyl (UCM-1306), an Orally Bioavailable Positive Allosteric Modulator of the Human Dopamine D1 Receptor for Parkinson’s Disease
Source: J Med Chem. 2022 Aug 31;65(18):12256–72. doi: 10.1021/acs.jmedchem.2c00949 (PMC9511493; doi:10.1021/acs.jmedchem.2c00949)
Supplement: Supplementary file 1 — jm2c00949_si_001.pdf [file jm2c00949_si_001.pdf]

Supporting Information

2-(Fluoromethoxy)-4'-(*S*-methanesulfonimidoyl)-1,1'-biphenyl (UCM-1306), an Orally Bioavailable Positive Allosteric Modulator of the Human Dopamine D_1 Receptor for Parkinson's Disease

Javier García-Cárceles,[a] Henar Vázquez-Villa,[a] José Brea,[b] David Ladron de Guevara-Miranda,[c] Giovanni Cincilla,[d] Melchor Sánchez-Martínez,[d] Anabel Sánchez-Merino,[a] Sergio Algar,[a] María Teresa de los Frailes,[e] Richard S. Roberts,[e] Juan A. Ballesteros,[f] Fernando Rodríguez de Fonseca,[c] Bellinda Benhamú*,[a] María I. Loza,*,[b,e] and María L. López-Rodríguez*,[a]

[a] Departamento de Química Orgánica, Universidad Complutense de Madrid, E-28040 Madrid, Spain.
[b] Biofarma Research group. USEF Screening Platform, CIMUS, USC, E-15782 Santiago de Compostela, Spain.
[c] Instituto de Investigación Biomédica de Málaga (IBIMA), E-29010 Málaga, Spain.
[d] Molomics S.L., Parc Científic de Barcelona, Baldiri Reixac 4-8, E-08028 Barcelona, Spain.
[e] Fundación Kærtor. Edificio EMPRENDIA. Planta 2, Oficina 4. Campus Vida, E-15706 Santiago de Compostela, España.
[f] ViviaBiotech S.L., Parque Científico de Madrid, E-28760 Madrid, Spain.

Corresponding authors: mluzlr@ucm.es, mabel.loza@usc.es, bellinda.benhamu@quim.ucm.es

Table of Contents

1. Tables and Figures S2
2. Chemistry S8
3. ADMET Assays S34
4. NMR Spectra of Compounds **3** and **26** S38
5. HPLC Traces of Compounds **3**, and *rac*, (*S*)-, (*R*)-**26** S40
6. References S43

1. Tables and Figures

Table S1. ADMET profile for compound **3**.

Solubility (µM)[a]	15
HSA binding (%)[b]	>99 (K_d = $4.34 \cdot 10^{-6}$ M)
P (cm/s)[c]	$20 \cdot 10^{-6}$
hERG inhibition (%)[d]	11 ± 2
Mouse serum stability (%)[e]	50
CYP2C9 (%)[f]	inactive
CYP2D6 (%)[f]	inactive
CYP1A2 (IC_{50}, µM)[f]	0.83 ± 0.17
CYP2C19 (IC_{50}, µM)[f]	6.53 ± 1.12
CYP34A (IC_{50}, µM)[f]	2.82 ± 0.32

[a] Maximum solubility measured by nephelometry; [b] Binding to human serum albumin (HSA) determined at a concentration of 5 µM; [c] Permeability in the parallel artificial membrane permeability assay (PAMPA); [d] Blockade of the K^+ channel current at a concentration of 10 µM; [e] Remaining compound quantified after 4 h; [f] Fluorescence-based inhibition assay with a panel of cytochromes P450 (CYP450).

Table S2. Plasma and brain concentrations achieved for compound **26** in the locomotor activity study.

**Plasma concentration (ng/mL)**			**Brain concentration (ng/mL)**		
**Animal**	**30 min**	**60 min**	**Animal**	**30 min**	**60 min**
1	9687.04	6187.65	1	7342.92	5552.94
2	9994.87	1662.96	2	7185.33	2620.14
3	7753.96	6716.72	3	5537.43	4178.34
**Mean**	**9145.3**	**4855.8**	**Mean**	**6688.6**	**4117.1**
SD	1214.7	2777.7	SD	1000.0	1467.4
SEM	701.3	1603.7	SEM	577.4	847.2

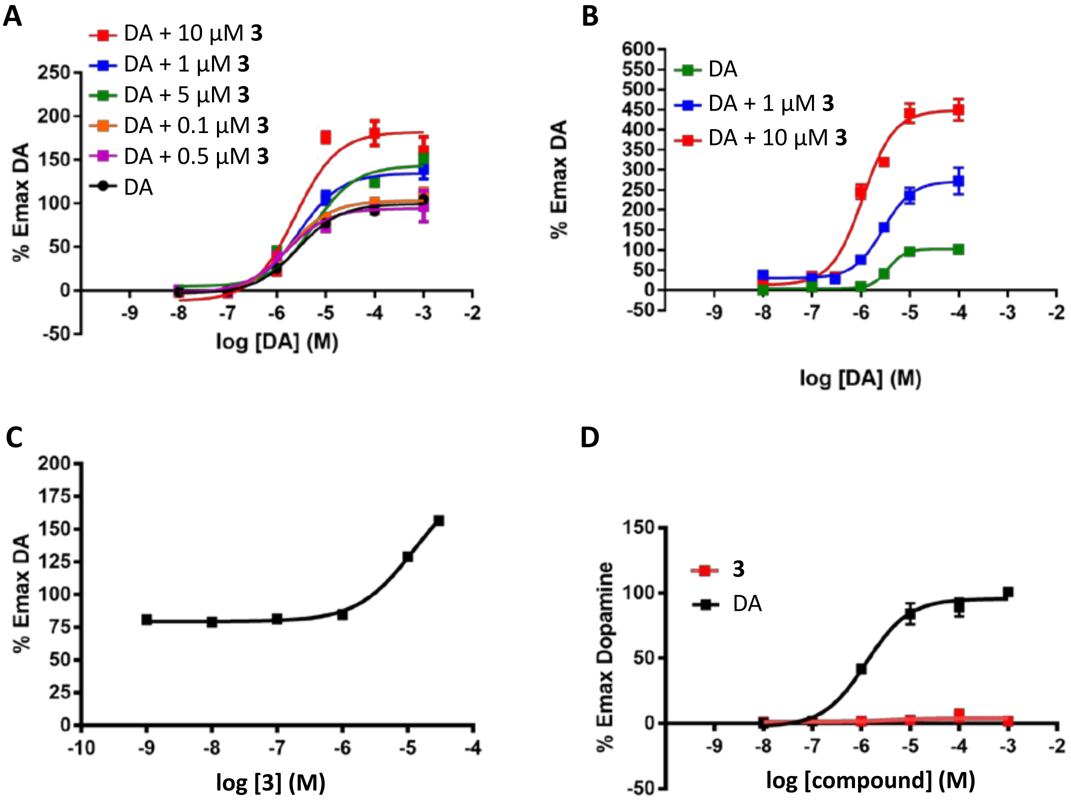

Figure S1. (A) Concentration-response curves in human D_1R for cAMP production of DA alone (black) and in the presence of different concentrations of compound **3**. (B) Concentration-response curves in mouse D_1R of DA alone (black) and in the presence of different concentrations of compound **3**. (C) Concentration-response curve in human D_1R of compound **3** in the presence of DA EC_{70} concentration. (D) Concentration-response curves in human D_1R of DA (black) and compound **3** (red).

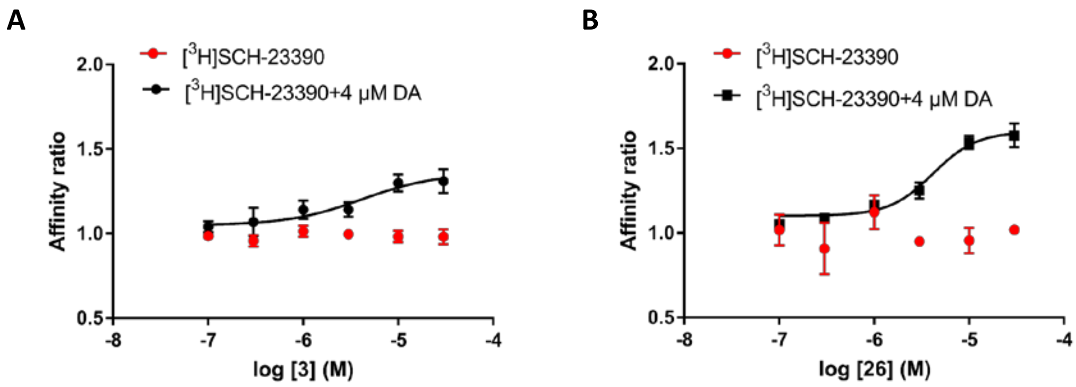

Figure S2. Concentration-response curves of compounds **3** (A) and **26** (B) over affinity ratio of [^{3}H]SCH-23390 in the absence (red) and in the presence (black) of 4 μM DA. Points represent the mean±SD (vertical bars) of triplicate determinations.

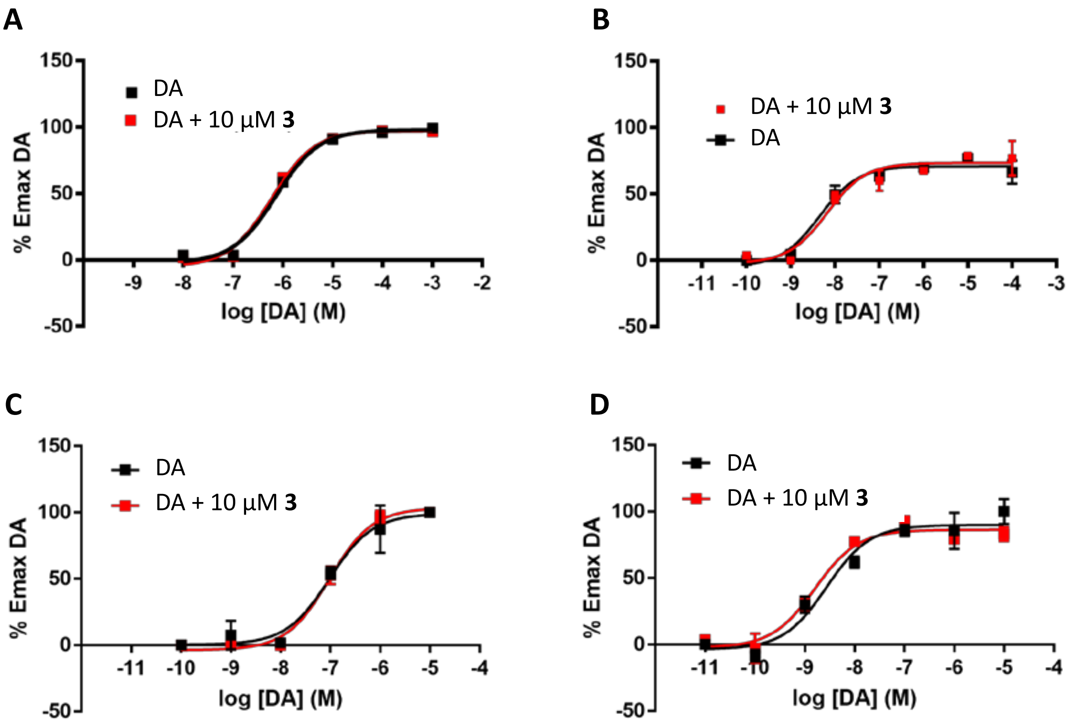

Figure S3. Selectivity profile of compound **3** towards human D_2 (A), D_3 (B), D_4 (C), and D_5 (D) receptors.

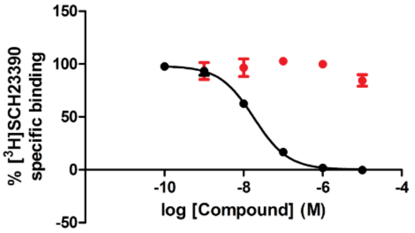

Figure S4. Radioligand competition assays measuring the displacement of [^{3}H]SCH-23390 by compound **3** (red) and haloperidol (black).

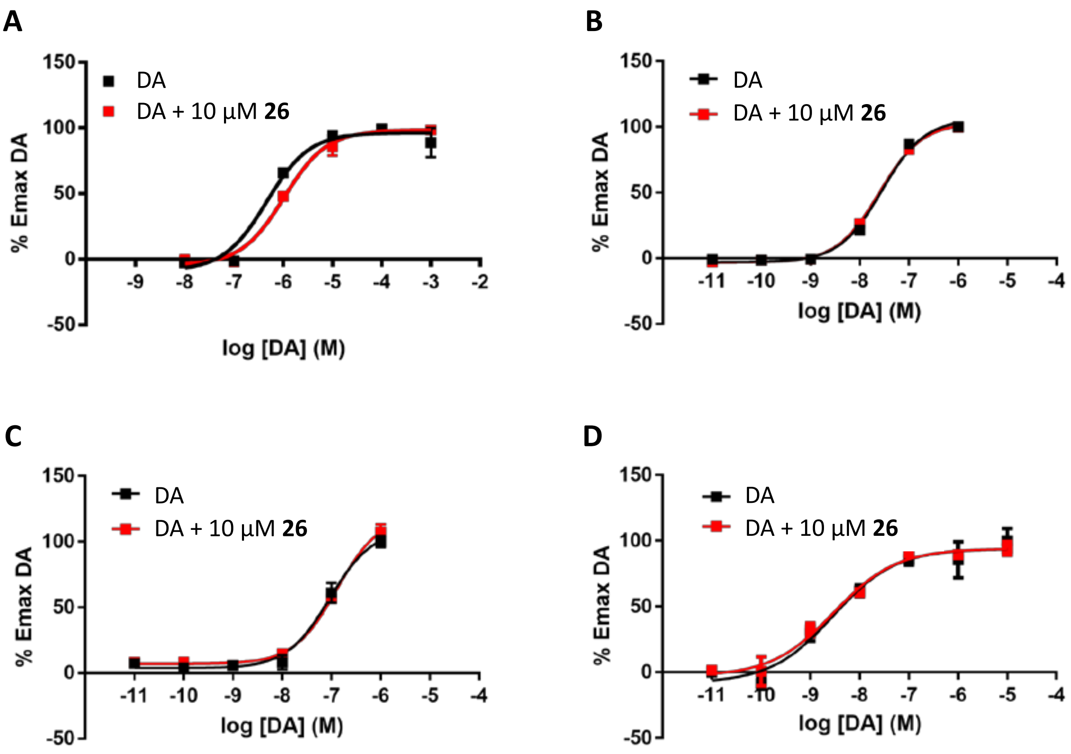

Figure S5. Selectivity profile of compound **26** towards human D_2 (A), D_3 (B), D_4 (C) and D_5 (D) receptors.

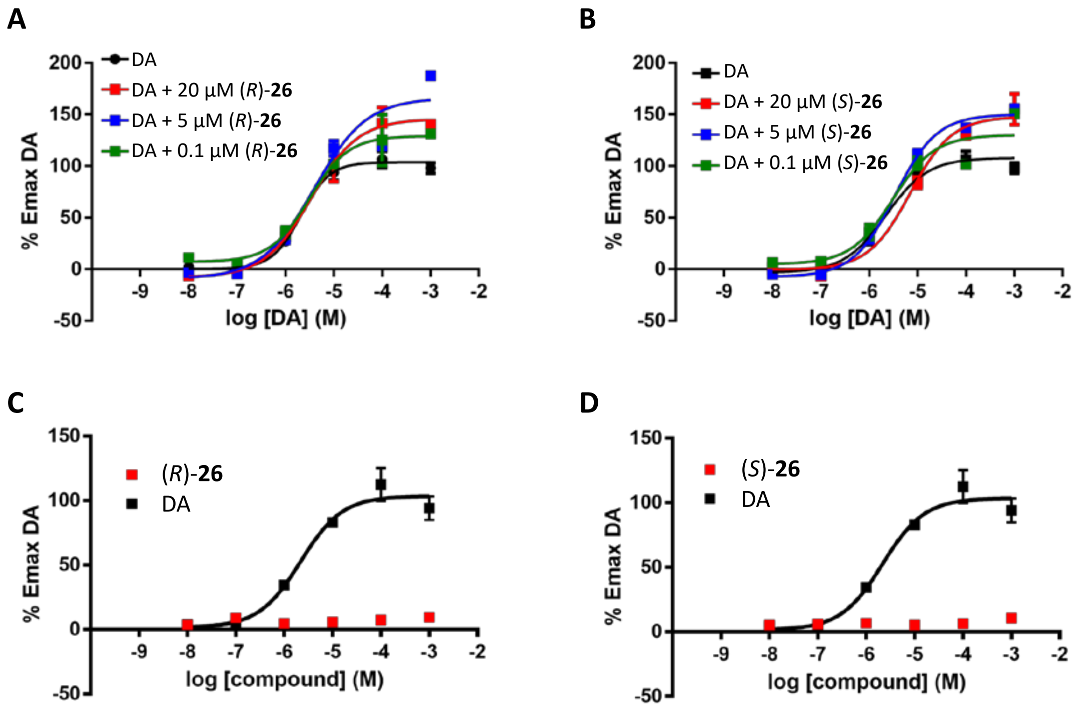

Figure S6. Pharmacological characterization of (*R*)- and (*S*)-**26** for cAMP production in human D_1R. Concentration-response curves induced by DA alone (black) and in the presence of different concentrations of (*R*)-**26** (A) and (*S*)-**26** (B). Concentration-response curves of DA and (*R*)-**26** (C) and (*S*)-**26** (D).

2. Chemistry

2.1. General

For the general considerations regarding the synthesis of the compounds, see the Experimental Section of the paper.

The general procedures for Suzuki-Miyaura, mono- and difluoroalkylation reactions mentioned along this section are described in the Experimental Section of the manuscript.

Table S3. Gradients of the mobile phases used in the HPLC-MS analysis of the synthetized compounds.

**I**		**II**		**III**		**IV**		**V**		**VI**	
t (min)	%B	t (min)	%B	t (min)	%B	t (min)	%B	t (min)	%B	t (min)	%B
0	0	0	0	0	60	0	40	0	0	0	0
2	0	2	0	10	100	11	100	2	0	5	0
10	100	10	50	22	100	33	100	8	80	15	90
25	100	20	100	28	60	36	40	12	100	22	100
30	0	25	100	30	60	40	40	25	100	26	100
		30	0					30	0	30	0

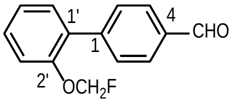

3

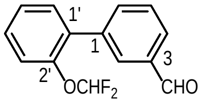

8

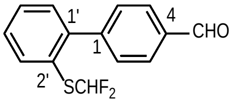

10

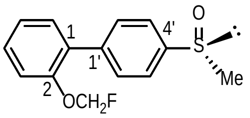

(*R*)-**23**

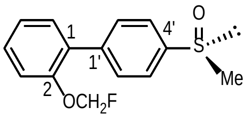

(*S*)-**23**

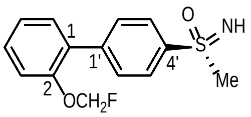

(*R*)-**26**

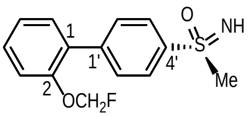

(*S*)-**26**

Figure S7. Numbered chemical structures for NMR assignation of final compounds **3**, **8**, **10**, (*R*)- and (*S*)-**23**, (*R*)- and (*S*)-**26**.

2.2. Synthesis and Characterization Data of Intermediates 11-13, 28-32, 37, 38, 40, 42, 44, 54, and 55, and Final Compounds 5-7, 9, and 22

3'-Hydroxy[1,1'-biphenyl]-4-carbaldehyde, 11. Following the general procedure for Suzuki-Miyaura reaction, compound **11** was obtained from 4-bromobenzaldehyde (99 mg, 0.535 mmol), (3-hydroxyphenyl)boronic acid (77 mg, 0.562 mmol) and Na_2CO_3 (227 mg, 2.14 mmol) in a THF/water mixture by heating under reflux overnight, as a white solid (60 mg, 57%). Chromatography: hexane to DCM. Spectroscopic data are in agreement with those reported.[1]

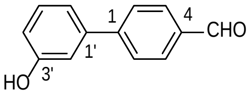

Mp: 177-178 ºC (lit.[1] mp 173-175 °C). R_f: 0.25 (hexane/DCM 7:3). IR (ATR): ν 3234 (OH), 1671 (CHO). ^{1}H-NMR (acetone-d_6): δ 6.90-6.94 (m, 1H, $H_{4'}$), 7.20-7.23 (m, 2H, $H_{2'}$, $H_{6'}$), 7.34 (t, J = 8.0, 1H, $H_{5'}$), 7.86 (d, J = 8.3, 2H, H_2, H_6), 8.01 (d, J = 8.4, 2H, H_3, H_5), 8.61 (s, 1H, OH), 10.09 (s, 1H, CHO). ^{13}C-NMR (acetone-d_6): δ 114.9 ($C_{2'}$), 116.4 ($C_{4'}$), 119.3 ($C_{6'}$), 128.4 (C_2, C_6), 130.9 (C_3, C_5), 131.0 ($C_{5'}$), 136.6 (C_4), 141.9 ($C_{1'}$), 147.6 (C_1), 158.9 ($C_{3'}$), 192.6 (CHO). MS (ESI, *m/z*, %): 197.0 ([M - H]$^-$, 100).

2'-Hydroxy[1,1'-biphenyl]-4-carbaldehyde, 12. Following the general procedure for Suzuki-Miyaura reaction, compound **12** was obtained from 2-bromophenol (98 mg, 0.566 mmol), (4-formylphenyl)boronic acid (89 mg, 0.594 mmol) and Na_2CO_3 (240 mg, 2.26 mmol) in a THF/water mixture by heating at 120 ºC under MW irradiation for 20 min, as a white solid (81 mg, 72%). Chromatography: hexane to DCM.

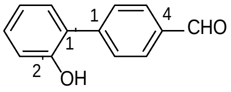

Mp: 118-120 ºC. R_f: 0.29 (DCM). IR (ATR): ν 3524 (OH), 1683 (CHO). ^{1}H-NMR (acetone-d_6): δ 6.98 (td, J = 7.4, 1.1, 1H, $H_{5'}$), 7.04 (dd, J = 8.1, 1.0, 1H, $H_{3'}$), 7.25 (ddd, J = 8.0, 7.4, 1.7, 1H, $H_{4'}$), 7.39 (dd, J = 7.6, 1.7, 1H, $H_{6'}$), 7.84 (d, J = 8.3, 2H, H_2, H_6), 7.96 (d, J = 8.4, 2H, H_3, H_5), 8.67 (br s, 1H, OH), 10.07 (s, 1H, CHO). ^{13}C-NMR (acetone-d_6): δ 116.7 ($C_{3'}$), 120.6 ($C_{5'}$), 127.5 ($C_{1'}$), 129.5 (C_3, C_5), 129.9 ($C_{4'}$), 130.3 (C_2, C_6), 131.0 ($C_{6'}$), 135.5 (C_4), 145.5 (C_1), 154.8 ($C_{2'}$), 192.1 (CHO). HPLC (Gradient-VI, column C3, t_R, min): 10.39. MS (ESI, *m/z*, %): 197.0 ([M - H]$^-$, 100).

2'-Hydroxy[1,1'-biphenyl]-3-carbaldehyde, 13. Following the general procedure for Suzuki-Miyaura reaction, compound **13** was obtained from 2-bromophenol (118 mg, 0.680 mmol), (3-formylphenyl)boronic acid (110 mg, 0.714 mmol) and Na_2CO_3 (288 mg,

2.72 mmol) in a THF/water mixture by heating at 120 ºC under MW irradiation for 20 min, as a white solid (70 mg, 52%). Chromatography: hexane to hexane/EtOAc 95:5.

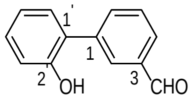

Mp: 93-94 ºC. R_f: 0.26 (hexane/EtOAc 9:1). IR (ATR): ν 3367 (OH), 1682 (CHO). ^{1}H-NMR ($CDCl_3$): δ 5.02 (br s, 1H, OH), 6.98 (dd, J = 8.5, 1.2, 1H, $H_{3'}$), 7.05 (td, J = 7.5, 1.1, 1H, $H_{5'}$), 7.26-7.33 (m, 2H, $H_{4'}$, $H_{6'}$), 7.66 (t, J = 7.6, 1H, H_5), 7.81 (dt, J = 7.7, 1.5, 1H, H_6), 7.91 (dt, J = 7.6, 1.5, 1H, H_4), 8.05 (m, 1H, H_2), 10.09 (s, 1H, CHO). ^{13}C-NMR ($CDCl_3$): δ 116.5 ($C_{3'}$), 121.4 ($C_{5'}$), 127.0 ($C_{1'}$), 128.8 (C_4), 129.7 ($C_{6'}$), 129.8 (C_5), 130.6 (C_2), 130.8 ($C_{4'}$), 135.4 (C_6), 136.9 (C_3), 138.8 (C_1), 152.6 ($C_{2'}$), 192.5 (CHO). HPLC (Gradient-I, column C18, t_R, min): 15.53. MS (ESI, *m/z*, %): 196.9 ($[M - H]^-$, 100).

1-(2'-Hydroxy[1,1'-biphenyl]-4-yl)ethan-1-one, 28. Following the general procedure for Suzuki-Miyaura reaction, compound **28** was obtained from 2-bromophenol (425 mg, 2.41 mmol), (4-acetylphenyl)boronic acid (484 mg, 2.89 mmol) and Na_2CO_3 (511 mg, 4.82 mmol) in a toluene/water/ethanol mixture by heating under reflux, as a white solid (220 mg, 43%). Chromatography: hexane to DCM. Spectroscopic data were in agreement with those reported.[2]

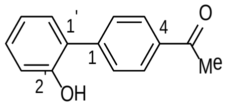

Mp: 145-147 ºC (lit.[2] mp 145-148 °C). R_f: 0.35 (DCM). IR (ATR): ν 3311 (OH), 1664 (CO). ^{1}H-NMR ($CDCl_3$): δ 2.66 (s, 3H, CH_3), 5.15 (br s, 1H, OH), 6.97-7.01 (m, 1H, $H_{3'}$), 7.04 (td, J = 7.5, 1.0, 1H, $H_{5'}$), 7.28-7.34 (m, 2H, $H_{4'}$, $H_{6'}$), 7.64 (d, J = 8.5, 2H, H_2, H_6), 8.08 (d, J = 8.5, 2H, H_3, H_5). MS (ESI, *m/z*, %): 210.9 ($[M - H]^-$, 100).

Methyl 2'-hydroxy[1,1'-biphenyl]-4-carboxylate, 29. Following the general procedure for Suzuki-Miyaura reaction, compound **29** was obtained from 2-bromophenol (201 mg, 1.16 mmol), [(4-methoxycarbonyl)phenyl]boronic acid (217 mg, 1.21 mmol) and Na_2CO_3 (246 mg, 2.32 mmol) in a toluene/water/ethanol mixture by heating at 100 ºC under MW irradiation for 20 min, as a white solid (41 mg, 16%). Chromatography: hexane to hexane/EtOAc 8:2 Spectroscopic data were in agreement with those reported.[3]

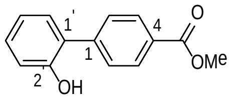

Mp: 128-130 ºC. R_f: 0.39 (hexane/EtOAc 8:2). IR (ATR): ν 3398 (OH), 1696 (CO). ^{1}H-NMR (acetone-d_6): δ 3.90 (s, 3H, CH_3), 6.96 (td, J = 7.5, 0.8, 1H, $H_{5'}$), 7.02 (d, J = 8.1,

1H, $H_{3'}$), 7.23 (td, *J* = 8.0, 1.6, 1H, $H_{4'}$), 7.36 (dd, *J* = 7.6, 1.6, 1H, $H_{6'}$), 7.75 (d, *J* = 8.4, 2H, H_2, H_6), 8.04 (d, *J* = 8.4, 2H, H_3, H_5), 8.57 (s, 1H, OH). ^{13}C-NMR (acetone-d_6): δ 52.3 (CH_3), 117.2 ($C_{3'}$), 121.0 ($C_{5'}$), 128.1 ($C_{1'}$), 129.2 (C_4), 129.9 (C_3, C_5), 130.2 ($C_{4'}$), 130.3 (C_2, C_6), 131.4 ($C_{6'}$), 144.6 (C_1), 155.2 ($C_{2'}$), 167.2 (CO). MS (ESI, *m/z*, %): 227.1 ($[M - H]^-$, 100).

2'-Hydroxy[1,1'-biphenyl]-4-sulfonamide, 30. Following the general procedure for Suzuki-Miyaura reaction, compound **30** was obtained from 4-bromobenzenesulfonamide (100 mg, 0.424 mmol), (2-hydroxyphenyl)boronic acid (64 mg, 0.466 mmol) and Na_2CO_3 (90 mg, 0.848 mmol) in a toluene/water/ethanol mixture by heating at 120 ºC under MW irradiation for 20 min, as a solid (96 mg, 90%). Chromatography: hexane to hexane/EtOAc 6:4.

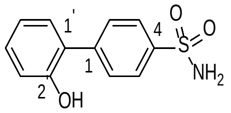

Mp: 204-205 ºC. *Rf*: 0.15 (hexane/EtOAc 2:8). IR (ATR): ν 3523 (OH), 3338, 3246 (NH_2), 1331, 1162 (SO_2). ^{1}H-NMR (acetone-d_6): δ 6.58 (br s, 2H, NH_2), 6.97 (t, *J* = 7.5, 1H, $H_{5'}$), 7.02 (d, *J* = 8.0, 1H, $H_{3'}$), 7.24 (td, *J* = 8.0, 1.4, 1H, $H_{4'}$), 7.36 (dd, *J* = 7.7, 1.4, 1H, $H_{6'}$), 7.77 (d, *J* = 8.4, 2H, H_2, H_6), 7.92 (d, *J* = 8.2, 2H, H_3, H_5), 8.65 (s, 1H, OH). ^{13}C-NMR (acetone-d_6): δ 117.1 ($C_{3'}$), 121.0 ($C_{5'}$), 126.6 (C_3, C_5), 127.8 ($C_{1'}$), 130.3 ($C_{4'}$), 130.5 (C_2, C_6), 131.4 ($C_{6'}$), 143.1 (C_1), 143.5 (C_4), 155.1 ($C_{2'}$). HPLC (Gradient-I, column C18, t_R, min): 14.12. MS (ESI, *m/z*, %): 267.0 ($[M + NH_4]^+$, 100).

2'-Hydroxy-*N*-methyl[1,1'-biphenyl]-4-sulfonamide, 31. Following the general procedure for Suzuki-Miyaura reaction, compound **31** was obtained from 4-bromo-*N*-methylbenzenesulfonamide (100 mg, 0.400 mmol), (2-hydroxyphenyl)boronic acid (61 mg, 0.440 mmol) and Na_2CO_3 (85 mg, 0.800 mmol) in a toluene/water/ethanol mixture by heating at 120 ºC under MW irradiation for 20 min, as a solid (100 mg, 95%). Chromatography: hexane to hexane/EtOAc 6:4.

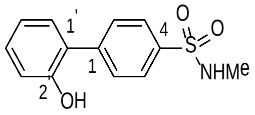

Mp: 140-141 ºC. *Rf*: 0.18 (hexane/EtOAc 2:8). IR (ATR): ν 3313 (NH, OH), 1316, 1159 (SO_2). ^{1}H-NMR (acetone-d_6): δ 2.62 (d, *J* = 5.2, 3H, CH_3), 6.33 (br s, 1H, NH), 6.97 (td, *J* = 7.5, 1.1, 1H, $H_{5'}$), 7.03 (dd, *J* = 8.1, 1.0, 1H, $H_{3'}$), 7.25 (ddd, *J* = 8.1, 7.4, 1.7, 1H, $H_{4'}$), 7.37 (dd, *J* = 7.6, 1.6, 1H, $H_{6'}$), 7.82 (d, *J* = 8.7, 2H, H_2, H_6), 7.87 (d, *J* = 8.7 Hz, 2H, H_3, H_5), 8.64 (s, 1H, OH). ^{13}C-NMR (acetone-d_6): δ 29.5 (CH_3), 117.2 ($C_{3'}$), 121.1 ($C_{5'}$),

126.6 (C_3, C_5), 127.6 ($C_{1'}$), 130.4 ($C_{4'}$), 130.7 (C_2, C_6), 131.4 ($C_{6'}$), 138.7 (C_1), 143.9 (C_4), 155.2 ($C_{2'}$). HPLC (Gradient-I, column C18, t_R, min): 15.07. MS (ESI, *m/z*, %): 262.0 ([M - H]$^-$, 100).

4'-(Methylsulfanyl)[1,1'-biphenyl]-2-ol, 32. Following the general procedure for Suzuki-Miyaura reaction, compound **32** was obtained from 1-bromo-(methylsulfanyl)benzene (150 mg, 0.716 mmol), (2-hydroxyphenyl)boronic acid (112 mg, 0.788 mmol) and Na_2CO_3 (152 mg, 1.43 mmol) in a toluene/water/ethanol mixture by heating at 120 ºC under MW irradiation for 20 min, as an off-white solid (141 mg, 91%). Chromatography: hexane to hexane/EtOAc 9:1. Spectroscopic data were in agreement with those reported.[4]

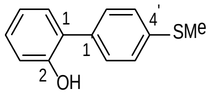

Mp: 80-81 ºC (lit.[4] mp 81-83 °C). *Rf*: 0.44 (hexane/EtOAc 6:4). IR (ATR): ν 3530, 3425 (OH), 1480, 1447 (Ar). ^{1}H-NMR ($CDCl_3$): δ 2.54 (s, 3H, CH_3), 5.13 (br s, 1H, OH), 6.97-7.03 (m, 2H, H_3, H_5), 7.22-7.30 (m, 2H, H_4, H_6), 7.36-7.43 (m, 4H, $H_{2'}$, $H_{3'}$, $H_{5'}$, $H_{6'}$). ^{13}C-NMR ($CDCl_3$): δ 15.8 (CH_3), 116.0 (C_3), 121.1 (C_5), 127.2 ($C_{3'}$, $C_{5'}$), 127.7 (C_1), 129.2 (C_4), 129.6 ($C_{2'}$, $C_{6'}$), 130.3 (C_6), 133.8 ($C_{1'}$), 138.6 ($C_{4'}$), 152.6 (C_2). MS (ESI, *m/z*, %): 214.8 ([M - H]$^-$, 100).

4'-(Methanesulfinyl)[1,1'-biphenyl]-2-ol, 37. Following the general procedure for Suzuki-Miyaura reaction, compound **37** was obtained from **35** (100 mg, 0.493 mmol), (2-hydroxyphenyl)boronic acid (81 mg, 0.592 mmol) and Na_2CO_3 (105 mg, 0.986 mmol) in a toluene/water/ethanol mixture by heating at 120 ºC under MW irradiation for 20 min, as a solid (106 mg, 93%). Chromatography: DCM to DCM/EtOAc 8:2.

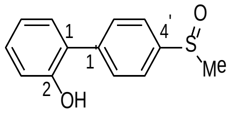

Mp: 179-180 ºC. *Rf*: 0.31 (DCM/EtOAc 6:4). IR (ATR): ν 3261 (OH), 1026 (SO). ^{1}H-NMR (methanol-d_4): δ 2.85 (s, 3H, CH_3), 6.90-6.95 (m, 2H, H_3, H_5), 7.21 (td, J = 7.7, 1.5, 1H, H_4), 7.30 (d, J = 7.5, 1H, H_6), 7.73 (d, J = 8.4, 2H, $H_{2'}$, $H_{6'}$), 7.81 (d, J = 8.4, 2H, $H_{3'}$, $H_{5'}$). ^{13}C-NMR (methanol-d_4): δ 43.5 (CH_3), 117.1 (C_3), 121.0 (C_5), 124.5 ($C_{3'}$, $C_{5'}$), 128.3 (C_1), 130.4 (C_4), 131.6 (C_6, $C_{2'}$, $C_{6'}$), 143.6 ($C_{1'}$), 144.1 ($C_{4'}$), 155.7 (C_2). HPLC (Gradient-II, column C18, t_R, min): 21.33. MS (ESI, *m/z*, %): 230.7 ([M - H]$^-$, 100).

4'-(Ethanesulfinyl)[1,1'-biphenyl]-2-ol, 38. Following the general procedure for Suzuki-Miyaura reaction, compound **38** was obtained from **36** (130 mg, 0.559 mmol), (2-

hydroxyphenyl)boronic acid (95 mg, 0.671 mmol) and Na_2CO_3 (119 mg, 1.12 mmol) in a toluene/water/ethanol mixture by heating at 120 ºC under MW irradiation for 20 min, as a solid (136 mg, 99%). Chromatography: hexane to hexane/EtOAc 6:4.

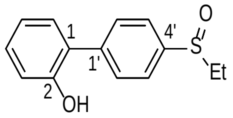

Mp: 210-211 ºC. *Rf*: 0.25 (hexane/EtOAc 2:8). IR (ATR): ν 3172 (OH), 1005 (SO). ^{1}H-NMR (methanol-*d*$_4$): δ 1.22 (t, *J* = 7.4, 3H, CH_3), 2.86-2.97 (m, 1H, 1/2CH_2), 3.00-3.12 (m, 1H, 1/2CH_2), 6.90-6.95 (m, 2H, H_3, H_5), 7.20 (td, *J* = 8.5, 2.7, 1H, H_4), 7.30 (dd, *J* = 8.2, 1.8, 1H, H_6), 7.68 (d, *J* = 8.6, 2H, $H_{2'}$, $H_{6'}$), 7.80 (d, *J* = 8.7, 2H, $H_{3'}$, $H_{5'}$). ^{13}C-NMR (methanol-*d*$_4$): δ 6.37 (CH_3), 50.9 (CH_2), 117.1 (C_3), 121.1 (C_5), 125.1 ($C_{3'}$, $C_{5'}$), 128.3 (C_1), 130.4 (C_4), 131.4 ($C_{2'}$, $C_{6'}$), 131.6 (C_6), 141.1 ($C_{1'}$), 144.0 ($C_{4'}$), 155.7 (C_2). HPLC (Gradient-I, column C18, t_R, min): 14.55. MS (ESI, *m/z*, %): 245.1 ([M - H]$^-$, 100).

4'-(*S*-Ethanesulfonimidoyl)[1,1'-biphenyl]-2-ol, 40. Following the general procedure for Suzuki-Miyaura reaction, compound **40** was obtained from **39** (130 mg, 0.525 mmol), (2-hydroxyphenyl)boronic acid (87 mg, 0.630 mmol) and Na_2CO_3 (111 mg, 1.05 mmol) in a toluene/water/ethanol mixture by heating at 120 ºC under MW irradiation for 20 min, as a solid (123 mg, 90%). Chromatography: hexane to hexane/EtOAc 1:1.

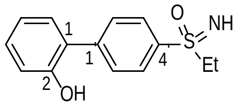

Mp: 141-143 ºC. *Rf*: 0.22 (hexane/EtOAc 2:8). IR (ATR): ν 3283 (OH, NH), 1277 (SO), 1099 (SO). ^{1}H-NMR (acetone-*d*$_6$): δ 1.19 (t, *J* = 7.1, 3H, CH_3), 3.16 (q, *J* = 7.4, 2H, CH_2), 3.15 (br s, 1H, NH), 6.97 (td, *J* = 7.6, 1.1, 1H, H_5), 7.04 (dd, *J* = 8.1, 0.9, 1H, H_3), 7.25 (td, *J* = 7.4, 1.8, 1H, H_4), 7.37 (dd, *J* = 7.6, 1.6, 1H, H_6), 7.82 (d, *J* = 8.5, 2H, $H_{2'}$, $H_{6'}$), 7.96 (d, *J* = 8.5, 2H, $H_{3'}$, $H_{5'}$). ^{13}C-NMR (acetone-*d*$_6$): δ 8.4 (CH_3), 52.5 (CH_2), 117.1 (C_3), 121.0 (C_5), 127.6 (C_1), 129.0 ($C_{3'}$, $C_{5'}$), 130.4 (C_4), 130.5 ($C_{2'}$, $C_{6'}$), 131.5 (C_6), 141.1 ($C_{1'}$), 144.2 ($C_{4'}$), 155.2 (C_2). HPLC (Gradient-I, column C18, t_R, min): 14.02. MS (ESI, *m/z*, %): 260.0 ([M - H]$^-$, 100).

4'-(*S*-Methanesulfonimidoyl)[1,1'-biphenyl]-2-ol, 42. Following the general procedure for Suzuki-Miyaura reaction, compound **42** was obtained from **41** (40 mg, 0.173 mmol), (2-hydroxyphenyl)boronic acid (27 mg, 0.190 mmol) and Na_2CO_3 (37 mg, 0.346 mmol) in a toluene/water/ethanol mixture by heating at 120 ºC under MW irradiation for 20 min, as a solid (24 mg, 56%). Chromatography: hexane to hexane/EtOAc 1:1.

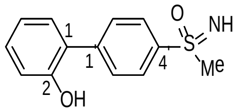

Mp: 196-197 ºC. R_f: 0.34 (hexane/EtOAc 2:8). IR (ATR): ν 3279 (OH, NH), 1100 (SO). ^{1}H-NMR (acetone-d_6, 500 MHz): δ 3.09 (s, 3H, CH_3), 6.97 (td, J = 7.4, 1.2, 1H, H_5), 7.04 (dd, J = 8.1, 0.6 Hz, 1H, H_3), 7.25 (td, J = 8.2, 1.7, 1H, H_4), 7.37 (dd, J = 7.6, 1.7, 1H, H_6), 7.81 (d, J = 8.6, 2H, $H_{2'}$, $H_{6'}$), 8.01 (d, J = 8.4, 2H, $H_{3'}$, $H_{5'}$), 8.65 (s, 1H, OH). ^{13}C-NMR (acetone-d_6, 125 MHz): δ 46.7 (CH_3), 117.2 (C_3), 121.1 (C_5), 127.8 (C_1), 128.2 ($C_{3'}$, $C_{5'}$), 130.4 (C_4), 130.6 ($C_{2'}$, $C_{6'}$), 131.5 (C_6), 143.5 ($C_{1'}$), 144.1 ($C_{4'}$), 155.2 (C_2). HPLC (Gradient-I, column C18, t_R, min): 13.35. MS (ESI, m/z, %): 248.1 ($[M + H]^+$, 100).

4'-(*N*,*S*-Dimethanesulfonimidoyl)[1,1'-biphenyl]-2-ol, 44. Following the general procedure for Suzuki-Miyaura reaction, compound **44** was obtained from **43** (108 mg, 0.434 mmol), (2-hydroxyphenyl)boronic acid (68 mg, 0.477 mmol) and Na_2CO_3 (92 mg, 0.868 mmol) in a toluene/water/ethanol mixture by heating at 100 ºC under MW irradiation for 20 min, as an off-white solid (51 mg, 45%). Chromatography: hexane to hexane/EtOAc 3:7.

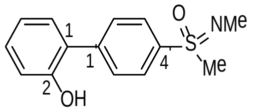

Mp: 206-207 ºC. R_f: 0.22 (hexane/EtOAc 1:1). IR (ATR): ν 3456 (OH), 1274, 1234 (SO). ^{1}H-NMR (methanol-d_4): δ 2.63 (s, 3H, NCH_3), 3.18 (s, 3H, SCH_3), 6.91-6.96 (m, 2H, H_3, H_5), 7.22 (td, J = 7.9, 1.6, 1H, H_4), 7.33 (dd, J = 7.3, 1.2, 1H, H_6), 7.84-7.91 (m, 4H, $H_{2'}$, $H_{3'}$, $H_{5'}$, $H_{6'}$). ^{13}C-NMR (methanol-d_4): δ 29.6 (NCH_3), 44.6 (SCH_3), 117.1 (C_3), 121.1 (C_5), 127.9 (C_1), 129.5 ($C_{3'}$, $C_{5'}$), 130.8 (C_4), 131.5 (C_6), 131.6 ($C_{2'}$, $C_{6'}$), 136.6 ($C_{1'}$), 146.0 ($C_{4'}$), 155.8 (C_2). HPLC (Gradient-I, column C18, t_R, min): 14.27. MS (ESI, m/z, %): 262.0 ($[M + H]^+$, 100).

3'-Chloro-4'-(*S*-methanesulfonimidoyl)[1,1'-biphenyl]-2-ol, 54. Following the general procedure for Suzuki-Miyaura reaction, compound **54** was obtained from **52** (80 mg, 0.298 mmol), (2-hydroxyphenyl)boronic acid (45 mg, 0.328 mmol) and Na_2CO_3 (63 mg, 0.596 mmol) in a toluene/water/ethanol mixture by heating at 100 ºC under MW irradiation for 20 min, as a solid (64 mg, 77%). Chromatography: hexane to hexane/EtOAc 1:1.

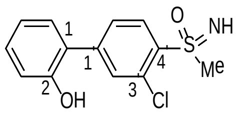

Mp: 97-98 ºC. R_f: 0.24 (hexane/EtOAc 1:1). IR (ATR): ν 3414 (NH, OH), 1364 (SO). ^{1}H-NMR (acetone-d_6): δ 3.27 (s, 3H, CH_3), 6.99 (td, J = 7.6, 1.1, 1H, H_5), 7.04 (d, J = 8.1, 1H, H_3), 7.28 (ddd, J = 8.1, 7.6, 1.8, 1H, H_4), 7.41 (dd, J = 7.6, 1.6, 1H, H_6), 7.75 (dd, J = 8.2, 1.7, 1H, $H_{6'}$), 7.86 (d, J = 1.7, 1H, $H_{2'}$), 8.18 (d, J = 8.3, 1H, $H_{5'}$). ^{13}C-NMR (acetone-d_6): δ 44.1 (CH_3), 117.2 (C_3), 121.2 (C_5), 126.1 (C_1), 128.8 ($C_{6'}$), 130.9 (C_4), 131.1 ($C_{5'}$), 131.3 (C_6), 132.2 ($C_{3'}$), 133.1 ($C_{2'}$), 140.7 ($C_{4'}$), 145.5 ($C_{1'}$), 155.2 (C_2). HPLC (Gradient-I, column C18, t_R, min): 12.17. MS (ESI, m/z, %): 280.0 ([M(^{35}Cl) - H]$^-$, 100), 281.9 ([M(^{37}Cl) - H]$^-$, 38).

2'-Chloro-4'-(*S*-methanesulfonimidoyl)[1,1'-biphenyl]-2-ol, 55. Following the general procedure for Suzuki-Miyaura reaction, compound **55** was obtained from **53** (35 mg, 0.130 mmol), (2-hydroxyphenyl)boronic acid (20 mg, 0.143 mmol) and Na_2CO_3 (28 mg, 0.260 mmol) in a toluene/water/ethanol mixture by heating at 120 ºC under MW irradiation for 20 min, as a solid (33 mg, 89%). Chromatography: hexane to hexane/EtOAc 8:2.

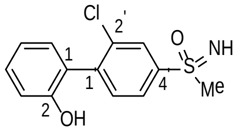

Mp: 175-176 ºC. R_f: 0.20 (hexane/EtOAc 3:7). IR (ATR): ν 3381 (NH), 1373, 1225 (SO). ^{1}H-NMR (acetone-d_6, 500 MHz): δ 3.15 (s, 3H, CH_3), 6.95 (td, J = 7.5, 1.0, 1H, H_5), 7.02 (dd, J = 8.2, 0.6, 1H, H_3), 7.17 (dd, J = 7.5, 1.6, 1H, H_6), 7.29 (ddd, J = 8.2, 7.6, 1.6, 1H, H_4), 7.58 (d, J = 8.0, 1H, $H_{6'}$), 7.94 (dd, J = 8.0, 1.8, 1H, $H_{5'}$), 8.05 (d, J = 1.8, 1H, $H_{3'}$). ^{13}C-NMR (acetone-d_6, 125 MHz): δ 46.4 (CH_3), 116.7 (C_3), 120.3 (C_5), 126.3 (C_1), 126.6 ($C_{5'}$), 129.2 ($C_{3'}$), 130.7 (C_4), 131.4 (C_6), 133.6 ($C_{6'}$), 135.1 ($C_{2'}$), 143.2 ($C_{1'}$), 145.9 ($C_{4'}$), 155.2 (C_2). HPLC (Gradient-I, column C18, t_R, min): 14.13. MS (ESI, m/z, %): 279.9 ([M(^{35}Cl) - H]$^-$, 100), 282.0 ([M(^{37}Cl) - H]$^-$, 40).

4'-(Difluoromethoxy)[1,1'-biphenyl]-4-carbaldehyde, 5. Following the general procedure for Suzuki-Miyaura reaction, compound **5** was obtained from 1-bromo-4-(difluoromethoxy)benzene (145 mg, 0.649 mmol), (4-formylphenyl)boronic acid (117 mg, 0.779 mmol) and Na_2CO_3 (137 mg, 1.30 mmol) in a toluene/water/ethanol mixture by heating under reflux, as a white solid (92 mg, 57%). Chromatography: hexane.

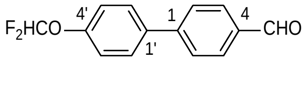

Mp: 140-141 ºC. R_f: 0.38 (hexane/DCM 7:3). IR (ATR): ν 1699 (CHO). ^{1}H-NMR ($CDCl_3$): δ 6.59 (t, J = 73.6, 1H, CHF_2), 7.24 (d, J = 8.7, 2H, $H_{3'}$, $H_{5'}$), 7.64 (d, J = 8.7,

2H, $H_{2'}$, $H_{6'}$), 7.72 (d, *J* = 8.2, 2H, H_2, H_6), 7.96 (d, *J* = 8.3, 2H, H_3, H_5), 10.06 (s, 1H, CHO). ^{13}C-NMR ($CDCl_3$): δ 115.9 (t, *J* = 260.6, CHF_2), 120.1 ($C_{3'}$, $C_{5'}$), 127.7 (C_2, C_6), 128.9 ($C_{2'}$, $C_{6'}$), 130.5 (C_3, C_5), 135.4 ($C_{1'}$), 137.1 (C_4), 146.1 (C_1), 151.5 (t, *J* = 2.7, $C_{4'}$), 192.0 (CHO). ^{19}F-NMR ($CDCl_3$): δ –83.3. HPLC (Gradient-I, column C3, t_R, min): 10.37. MS (ESI, *m/z*, %): 249.0 ($[M + H]^+$, 100).

3'-(Difluoromethoxy)[1,1'-biphenyl]-4-carbaldehyde, 6. Following the general procedure for Suzuki-Miyaura reaction, compound **6** was obtained from 1-bromo-3-(difluoromethoxy)benzene (154 mg, 0.692 mmol), (4-formylphenyl)boronic acid (125 mg, 0.831 mmol) and Na_2CO_3 (147 mg, 1.38 mmol) in a toluene/water/ethanol mixture by heating under reflux, as a colorless oil (155 mg, 90%). Chromatography: hexane.

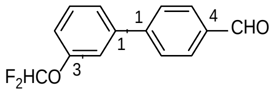

Rf: 0.41 (hexane/DCM 7:3). IR (ATR): ν 1695 (CHO). ^{1}H-NMR ($CDCl_3$): δ 6.58 (t, *J* = 73.6, 1H, CHF_2), 7.15-7.22 (m, 1H, $H_{4'}$), 7.38-7.40 (m, 1H, $H_{2'}$), 7.47-7.49 (m, 2H, $H_{5'}$, $H_{6'}$), 7.74 (d, *J* = 8.3, 2H, H_2, H_6), 7.97 (d, *J* = 8.4, 2H, H_3, H_5), 10.07 (s, 1H, CHO). ^{13}C-NMR ($CDCl_3$): δ 116.0 (t, *J* = 260.6, CHF_2), 118.9 ($C_{2'}$), 119.6 ($C_{4'}$), 124.6 ($C_{6'}$), 127.9 (C_2, C_6), 130.5 (C_3, C_5), 130.6 ($C_{5'}$), 135.8 (C_4), 141.9 ($C_{1'}$), 146.0 (C_1), 151.8 (t, *J* = 2.7, $C_{3'}$), 191.9 (CHO). ^{19}F-NMR ($CDCl_3$): δ –83.1. HPLC (Gradient-I, column C3, t_R, min): 11.78. MS (ESI, *m/z*, %): 249.0 ($[M + H]^+$, 100).

2'-(Difluoromethoxy)[1,1'-biphenyl]-4-carbaldehyde, 7. Following the general procedure for Suzuki-Miyaura reaction, compound **7** was obtained from 1-bromo-2-(difluoromethoxy)benzene (154 mg, 0.692 mmol), (4-formylphenyl)boronic acid (125 mg, 0.831 mmol) and Na_2CO_3 (146 mg, 1.38 mmol) in a toluene/water/ethanol mixture by heating under reflux, as an off-white solid (104 mg, 61%). Chromatography: hexane.

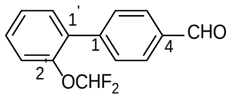

Mp: 48-50 ºC. *Rf*: 0.40 (hexane/DCM 7:3). IR (ATR): ν 1696 (CHO). ^{1}H-NMR ($CDCl_3$): δ 6.39 (t, *J* = 73.7, 1H, CHF_2), 7.30-7.37 (m, 4H, $H_{3'}$, $H_{5'}$), 7.41-7.46 (m, 4H, $H_{4'}$, $H_{6'}$), 7.68 (d, *J* = 8.3, 2H, H_2, H_6), 7.96 (d, *J* = 8.3, 2H, H_3, H_5), 10.08 (s, 1H, CHO). ^{13}C-NMR ($CDCl_3$): δ 116.1 (t, *J* = 260.8, CHF_2), 120.3 ($C_{3'}$), 126.1 ($C_{5'}$), 129.8 (C_3, C_5), 129.9 ($C_{4'}$), 130.3 (C_2, C_6), 131.4 ($C_{6'}$), 133.0 ($C_{1'}$), 135.5 (C_4), 143.5 (C_1), 148.2 (t, *J* = 2.5, $C_{2'}$), 192.1 (CHO). ^{19}F-NMR ($CDCl_3$): δ –82.5. HPLC (Gradient-I, column C3, t_R, min): 11.53. MS (ESI, *m/z*, %): 249.0 ($[M + H]^+$, 100).

2'-[(Fluoromethyl)sulfanyl][1,1'-biphenyl]-4-carbaldehyde, 9. Following the general procedure for Suzuki-Miyaura reaction, compound **9** was obtained from **14** (98 mg, 0.436 mmol), (4-formylphenyl)boronic acid (92 mg, 0.872 mmol) and Na_2CO_3 (147 mg, 1.38 mmol) in a toluene/water/ethanol mixture by heating at 120 ºC under MW irradiation for 20 min, as an oil (52 mg, 49%). Chromatography: hexane to hexane/EtOAc 9:1.

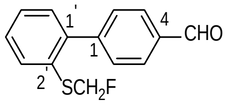

Rf: 0.38 (hexane/EtOAc 9:1). IR (ATR): ν 1701 (CHO). ^{1}H-NMR ($CDCl_3$): δ 5.63 (d, *J* = 52.6, 2H, CH_2F), 7.32 (dd, *J* = 7.2, 2.0, 1H, $H_{3'}$), 7.39 (td, *J* = 7.4, 1.6, 1H, $H_{5'}$), 7.44 (td, *J* = 7.4, 1.9, 1H, $H_{4'}$), 7.56 (d, *J* = 8.1, 2H, H_2, H_6), 7.72 (dd, *J* = 7.3, 1.8, 1H, $H_{6'}$), 7.97 (d, *J* = 8.2, 2H, H_3, H_5), 10.09 (s, 1H, CHO). ^{13}C-NMR ($CDCl_3$): δ 87.9 (d, *J* = 215.9, CH_2F), 127.8 ($C_{3'}$), 129.3 ($C_{5'}$), 129.6 (C_3, C_5), 130.2 ($C_{4'}$), 130.4 ($C_{6'}$), 130.4 (C_2, C_6), 133.2 (d, *J* = 3.0, $C_{1'}$), 135.5 (C_4), 141.8 (d, *J* = 2.5 Hz, $C_{2'}$), 146.6 (C_1), 192.0 (CHO). ^{19}F-NMR ($CDCl_3$): δ –183.2. HPLC (Gradient-I, column C3, t_R, min): 10.30. MS (ESI, *m/z*, %): 227.1 ($[M - F]^+$, 100), 247.1 ($[M + H]^+$, 36), 279.1 ($[M + MeOH + H]^+$, 41).

2'-(Fluoromethoxy)[1,1'-biphenyl]-4-carboxamide, 22. Following the general procedure for Suzuki-Miyaura reaction, compound **22** was obtained from **34** (121 mg, 0.590 mmol), (4-carbamoylphenyl)boronic acid (110 mg, 0.649 mmol) and Na_2CO_3 (125 mg, 1.18 mmol) in a toluene/water/ethanol mixture by heating at 170 ºC under MW irradiation for 10 min, as a solid (37 mg, 32%). Chromatography: preparative TLC in DCM/methanol 95:5.

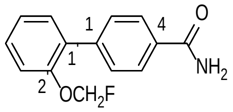

Mp: 166-168 ºC (hexane/methanol). *Rf*: 0.28 (DCM/methanol 95:5). IR (ATR): ν 3389, 3181 (NH_2), 1639 (CO). ^{1}H-NMR (acetone-d_6): δ 5.82 (d, *J* = 54.5, 2H, CH_2F), 6.66 (br s, 1H, NH), 7.24 (td, *J* = 7.7, 1.1, 1H, $H_{5'}$), 7.33 (d, *J* = 8.6, 1H, $H_{3'}$), 7.42-7.47 (m, 3H, $H_{4'}$, $H_{6'}$, NH), 7.61 (d, *J* = 8.5, 2H, H_2, H_6), 8.00 (d, *J* = 8.5 Hz, 2H, H_3, H_5). ^{13}C-NMR (acetone-d_6): δ 102.0 (d, *J* = 216.8, CH_2F), 116.6 ($C_{3'}$), 124.7 ($C_{5'}$), 128.2 (C_3, C_5), 130.2 (C_2, C_6), 130.3 ($C_{4'}$), 131.8 ($C_{6'}$), 131.9 (d, *J* = 1.0, $C_{1'}$), 134.1 (C_4), 141.9 (C_1), 154.6 (d, *J* = 3.0, $C_{2'}$), 168.8 ($CONH_2$). ^{19}F-NMR (acetone-d_6): δ –150.7. HPLC (Gradient-IV, column C18, t_R, min): 18.98. MS (ESI, *m/z*, %): 246.1 ($[M + H]^+$, 100).

2.3. Synthesis and Characterization Data of Intermediates 14, 34, 48, and 56, and Final Compounds 1, 2, 4, 16-20, 23-25, 27, 46, and 47

1-Bromo-2-[(fluoromethyl)sulfanyl]benzene, 14. Following the general procedure for the monofluoroalkylation reaction, compound **14** was obtained from 2-bromobenzenethiol (148 μL, 1.24 mmol) as an oil (211 mg, 77%). Chromatography: hexane.

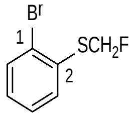

Rf: 0.56 (hexane/DCM 9:1). ^{1}H-NMR (CDCl$_3$): δ 5.79 (d, *J* = 52.5, 2H, CH$_2$F), 7.15 (td, *J* = 7.6, 1.6, 1H, H$_5$), 7.34 (td, *J* = 7.7, 1.4, 1H, H$_4$), 7.60 (d, *J* = 6.6, 2H, H$_3$, H$_6$). ^{13}C-NMR (CDCl$_3$): δ 86.8 (d, *J* = 216.3, CH$_2$F), 124.2 (d, *J* = 2.9, C$_1$), 128.4, 128.6 (C$_4$, C$_5$), 130.2 (d, *J* = 2.3, C$_3$), 133.3 (C$_6$), 136.0 (d, *J* = 3.4, C$_2$). ^{19}F-NMR (CDCl$_3$): δ –185.3.

1-Bromo-2-(fluoromethoxy)benzene, 34. Following the general procedure for the monofluoroalkylation reaction, compound **34** was obtained from 2-bromophenol (471 mg, 2.72 mmol) as a colorless oil (541 mg, 97%). Chromatography: hexane. Spectroscopic data were in agreement with those reported.[5]

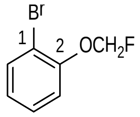

Rf: 0.46 (hexane/DCM 8:2). ^{1}H-NMR (CDCl$_3$): δ 5.76 (d, *J* = 54.2, 2H, CH$_2$F), 7.01 (td, *J* = 8.1, 1.4, 1H, H$_5$), 7.20 (d, *J* = 8.2, 1H, H$_3$), 7.32 (td, *J* = 8.3, 1.4, 1H, H$_4$), 7.59 (dd, *J* = 8.0, 1.4, 1H, H$_6$). ^{13}C-NMR (CDCl$_3$): δ 101.3 (d, *J* = 221.0, CH$_2$F), 113.4 (C$_1$), 117.5 (d, *J* = 1.3, C$_3$), 125.1 (C$_5$), 128.9 (C$_4$), 133.8 (C$_6$), 153.7 (d, *J* = 3.1, C$_2$). ^{19}F-NMR (CDCl$_3$): δ –149.6.

2-Bromo-3-(fluoromethoxy)pyridine, 48. Following the general procedure for the monofluoroalkylation reaction, compound **48** was obtained from 2-bromopyridin-3-ol (500 mg, 2.87 mmol) as a solid (428 mg, 73%), which was used in the next step without further purification.

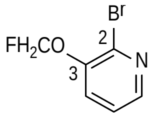

Mp: 37-38 ºC. *Rf*: 0.25 (hexane/DCM 1:1). ^{1}H-NMR (CDCl$_3$): δ 5.76 (d, *J* = 53.5, 2H, CH$_2$F), 7.27 (dd, *J* = 8.1, 4.6, 1H, H$_5$), 7.46 (dt, *J* = 8.1, 1.3, 1H, H$_4$), 8.16 (dd, *J* = 4.6, 1.5, 1H, H$_6$). ^{13}C-NMR (CDCl$_3$): δ 101.0 (d, *J* = 222.0, CH$_2$F), 123.7 (C$_5$), 124.5 (d, *J* =

1.6, C_4), 134.0 (d, J = 2.1, C_2), 144.8 (C_6), 150.6 (d, J = 3.0, C_3). ^{19}F-NMR ($CDCl_3$): δ −151.0. HPLC (Gradient-I, column C18, t_R, min): 19.10. MS (ESI, *m/z*, %): 205.6 ([M(^{79}Br) + H]$^+$, 92), 207.7 ([M(^{81}Br) + H]$^+$, 100).

2-(Fluoromethoxy)-4'-(methylsulfanyl)-1,1'-biphenyl, 56. Following the general procedure for the monofluoroalkylation reaction, compound **56** was obtained from **32** (40 mg, 0.185 mmol) as an oil (38 mg, 84%). Chromatography: hexane.

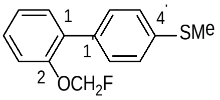

Rf: 0.35 (hexane/DCM 9:1). ^{1}H-NMR ($CDCl_3$): δ 2.54 (s, 3H, CH_3), 5.62 (d, J = 54.6, 2H, CH_2F), 7.17-7.26 (m, 2H, H_3, H_5), 7.32 (d, J = 8.5, 2H, $H_{3'}$, $H_{5'}$), 7.34-7.38 (m, 2H, H_4, H_6), 7.46 (d, J = 8.6, 2H, $H_{2'}$, $H_{6'}$). ^{13}C-NMR ($CDCl_3$): δ 15.9 (CH_3), 101.1 (d, J = 219.0, CH_2F), 116.2 (d, J = 1.2, C_3), 124.0 (C_5), 126.3 ($C_{3'}$, $C_{5'}$), 128.9 (C_4), 130.0 ($C_{2'}$, $C_{6'}$), 131.1 (C_6), 131.6 (d, J = 1.3, C_1), 134.6 ($C_{1'}$), 137.7 ($C_{4'}$), 153.7 (d, J = 3.1, C_2). ^{19}F-NMR ($CDCl_3$): δ −148.6. HPLC (Gradient-VI, column C3, t_R, min): 20.81. MS (ESI, *m/z*, %): 248.8 ([M + H]$^+$, 100), 228.8 ([M - F]$^+$, 63).

4'-(Fluoromethoxy)[1,1'-biphenyl]-4-carbaldehyde, 1. Following the general procedure for the monofluoroalkylation reaction, compound **1** was obtained from 4'-hydroxy[1,1'-biphenyl]-4-carbaldehyde (146 mg, 0.737 mmol) as a white solid (100 mg, 60%). Chromatography: hexane.

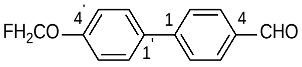

Mp: 86-87 ºC. *Rf*: 0.39 (hexane/DCM 7:3). IR (ATR): ν 1681 (CHO). ^{1}H-NMR ($CDCl_3$): δ 5.77 (d, J = 54.3, 2H, CH_2F), 7.19 (d, J = 8.6, 2H, $H_{3'}$, $H_{5'}$), 7.62 (d, J = 8.8, 2H, $H_{2'}$, $H_{6'}$), 7.72 (d, J = 8.2, 2H, H_2, H_6), 7.94 (d, J = 8.3, 2H, H_3, H_5), 10.05 (s, 1H, CHO). ^{13}C-NMR ($CDCl_3$): δ 100.7 (d, J = 219.8, CH_2F), 117.2 (d, J = 1.3, $C_{3'}$, $C_{5'}$), 127.5 (C_2, C_6), 128.9 ($C_{2'}$, $C_{6'}$), 130.4 (C_3, C_5), 135.1, 135.2 (C_4, $C_{1'}$), 146.4 (C_1), 157.3 (d, J = 3.1, $C_{4'}$), 191.9 (CHO). ^{19}F-NMR ($CDCl_3$): δ −151.6. HPLC (Gradient-I, column C3, t_R, min): 10.19. MS (ESI, *m/z*, %): 231.1 ([M + H]$^+$, 100).

3'-(Fluoromethoxy)[1,1'-biphenyl]-4-carbaldehyde, 2. Following the general procedure for the monofluoroalkylation reaction, compound **2** was obtained from **11** (51 mg, 0.257 mmol) as an oil (35 mg, 59%). Chromatography: hexane.

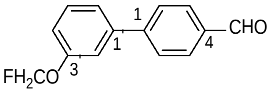

Rf: 0.41 (hexane/DCM 7:3). IR (ATR): ν 1699 (CHO). ^{1}H-NMR (acetone-d_6): δ 5.94 (d, *J* = 54.4, 2H, CH_2F), 7.18-7.22 (m, 1H, $H_{4'}$), 7.47-7.53 (m, 3H, $H_{2'}$, $H_{5'}$, $H_{6'}$), 7.92 (d, *J* = 8.4, 2H, H_2, H_6), 8.03 (d, *J* = 8.5, 2H, H_3, H_5), 10.11 (s, 1H, CHO). ^{13}C-NMR (acetone-d_6): δ 101.6 (d, *J* = 216.2, CH_2F), 116.2 (d, *J* = 1.1, $C_{2'}$), 117.2 (d, *J* = 1.0, $C_{4'}$), 123.1 ($C_{6'}$), 128.6 (C_2, C_6), 130.9 (C_3, C_5), 131.4 ($C_{5'}$), 136.8 (C_4), 142.3 ($C_{1'}$), 146.8 (C_1), 158.2 (d, *J* = 2.8, $C_{3'}$), 192.6 (CHO). ^{19}F-NMR (acetone-d_6): δ −151.2. HPLC (Gradient-I, column C3, t_R, min): 11.67. MS (ESI, *m/z*, %): 230.9 ([M + H]$^+$, 100).

2'-(Fluoromethoxy)[1,1'-biphenyl]-3-carbaldehyde, 4. Following the general procedure for the monofluoroalkylation reaction, compound **4** was obtained from **13** (30 mg, 0.151 mmol) as an oil (22 mg, 64%). Chromatography: preparative TLC in toluene/methanol 98:2.

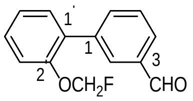

Rf: 0.32 (toluene/methanol 98:2). IR (ATR): ν 1697 (CHO). ^{1}H-NMR ($CDCl_3$): δ 5.68 (d, *J* = 54.4, 2H, CH_2F), 7.20-7.30 (m, 2H, $H_{3'}$, $H_{5'}$), 7.39-7.42 (m, 2H, $H_{4'}$, $H_{6'}$), 7.60 (t, *J* = 7.7, 1H, H_5), 7.80 (m, 1H, H_6), 7.88 (m, 1H, H_4), 8.02 (m, 1H, H_2), 10.08 (s, 1H, CHO). ^{13}C-NMR ($CDCl_3$): δ 100.9 (d, *J* = 218.2, CH_2F), 115.8 (d, *J* = 1.2, $C_{3'}$), 124.1 ($C_{5'}$), 128.5 (C_4), 128.9 ($C_{6'}$), 129.7 (C_5), 130.6 (d, *J* = 1.1, $C_{1'}$), 131.1 (C_2), 131.2 ($C_{4'}$), 135.8 (C_6), 136.5 (C_3), 138.9 (C_1), 153.7 (d, *J* = 3.3, $C_{2'}$), 192.4 (CHO). ^{19}F-NMR ($CDCl_3$): δ −148.9. HPLC (Gradient-I, column C3, t_R, min): 10.48. MS (ESI, *m/z*, %): 252.8 ([M + Na]$^+$, 100).

1-[2'-(Fluoromethoxy)[1,1'-biphenyl]-4-yl]ethan-1-one, 16. Following the general procedure for the monofluoroalkylation reaction, compound **16** was obtained from **28** (220 mg, 1.04 mmol) as a solid (195 mg, 77%). Chromatography: hexane.

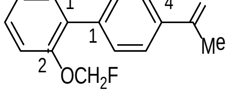

Mp: 70-71 ºC. *Rf*: 0.36 (hexane/DCM 7:3). IR (ATR): ν 1679 (CO). ^{1}H-NMR ($CDCl_3$): δ 2.65 (s, 3H, CH_3), 5.67 (d, *J* = 54.4, 2H, CH_2F), 7.19-7.28 (m, 2H, $H_{3'}$, $H_{5'}$), 7.38-7.44 (m, 2H, $H_{4'}$, $H_{6'}$), 7.62 (d, *J* = 8.4, 2H, H_2, H_6), 8.03 (d, *J* = 8.4, 2H, H_3, H_5). ^{13}C-NMR ($CDCl_3$): δ 26.8 (CH_3), 101.0 (d, *J* = 219.7, CH_2F), 116.1 (*J* = 1.1, $C_{3'}$), 124.1 ($C_{5'}$), 128.3 (C_3, C_5), 129.8 ($C_{4'}$), 129.9 (C_2, C_6), 131.0 (d, *J* = 1.3, $C_{1'}$), 131.1 ($C_{6'}$), 136.0 (C_4), 142.9 (C_1), 153.7 (d, *J* = 3.1, $C_{2'}$), 197.9 (CO). ^{19}F-NMR ($CDCl_3$): δ −148.9. HPLC (Gradient-I, column C18, t_R, min): 17.59. MS (ESI, *m/z*, %): 245.1 ([M + H]$^+$, 100).

Methyl 2'-(fluoromethoxy)[1,1'-biphenyl]-4-carboxylate, 17. Following the general procedure for the monofluoroalkylation reaction, compound **17** was obtained from **29** (40 mg, 0.175 mmol) as a solid (42 mg, 93%). Chromatography: hexane.

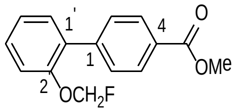

Mp: 61-62 ºC. R_f: 0.45 (hexane/EtOAc 9:1). IR (ATR): ν 1721(CO). ^{1}H-NMR ($CDCl_3$): δ 3.96 (s, 3H, CH_3), 5.66 (d, J = 54.4, 2H, CH_2F), 7.22 (td, J = 7.7, 1.1, 1H, $H_{5'}$), 7.27 (dd, J = 7.6, 1.0, 1H, $H_{3'}$), 7.39 (d, J = 7.6, 1H, $H_{6'}$), 7.37-7.43 (m, 1H, $H_{4'}$), 7.60 (d, J = 7.5, 2H, H_2, H_6), 8.10 (d, J = 8.6, 2H, H_3, H_5). ^{13}C-NMR ($CDCl_3$): δ 52.5 (CH_3), 101.3 (d, J = 219.6, CH_2F), 116.3 (d, J = 1.1, $C_{3'}$), 124.3 ($C_{5'}$), 129.3 ($C_{1'}$), 129.8 (C_3, C_5), 130.0 (C_2, C_6, $C_{4'}$), 131.3 (C_4), 131.4 ($C_{6'}$), 142.9 (C_1), 154.0 (d, J = 3.0, $C_{2'}$), 167.4 (CO). ^{19}F-NMR (acetone-d_6): δ −148.9. HPLC (Gradient-IV, column C3, t_R, min): 18.46. MS (ESI, *m/z*, %): 261.1 ([M + H]$^+$, 100).

2’-(Fluoromethoxy)[1,1'-biphenyl]-4-sulfonamide, 18. Following the general procedure for the monofluoroalkylation reaction, compound **18** was obtained from **30** (15 mg, 0.060 mmol) as a solid (5 mg, 31%). Chromatography: preparative TLC in DCM/EtOAc 85:15.

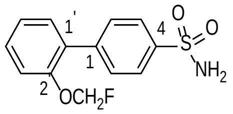

Mp: 153-154 ºC. R_f: 0.29 (DCM/EtOAc 85:15). IR (ATR): ν 3336, 3273 (NH_2), 1335, 1163 (SO_2). ^{1}H-NMR (acetone-d_6, 500 MHz): δ 5.83 (d, J = 54.4, 2H, CH_2F), 6.61 (br s, 2H, NH_2), 7.25 (td, J = 7.4, 1.0, 1H, $H_{5'}$), 7.34 (d, J = 8.2, 1H, $H_{3'}$), 7.43-7.48 (m, 2H, $H_{4'}$, $H_{6'}$), 7.69 (d, J = 8.6, 2H, H_2, H_6), 7.95 (d, J = 8.6, 2H, H_3, H_5). ^{13}C-NMR (acetone-d_6, 125 MHz): δ 101.9 (d, J = 215.6, CH_2F), 116.5 (d, J = 1.0, $C_{3'}$), 124.7 ($C_{5'}$), 126.7 (C_3, C_5), 130.7 ($C_{4'}$), 130.8 (C_2, C_6), 131.3 (d, J = 0.8, $C_{1'}$), 131.9 ($C_{6'}$), 142.5 (d, J = 1.2, C_1), 143.8 (d, J = 3.9, C_4), 154.5 (d, J = 2.9, $C_{2'}$). ^{19}F-NMR (acetone-d_6): δ −150.9. HPLC (Gradient-I, column C18, t_R, min): 14.86. MS (ESI, *m/z*, %): 299.0 ([M + NH_4]$^+$, 100).

2’-(Fluoromethoxy)-*N*-methyl[1,1'-biphenyl]-4-sulfonamide, 19. Following the general procedure for the monofluoroalkylation reaction, compound **19** was obtained from **31** (24 mg, 0.089 mmol) as an oil (12 mg, 45%). Chromatography: preparative TLC in DCM/methanol 95:5.

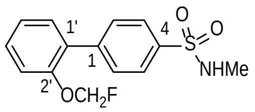

R_f: 0.38 (DCM/methanol 95:5). IR (ATR): ν 3295 (NH_2), 1324, 1163 (SO_2). ^{1}H-NMR (acetone-d_6): δ 2.63 (d, J = 5.1, 3H, CH_3), 5.84 (d, J = 54.3, 2H, CH_2F), 6.39 (br q, J = 4.9, 1H, NH), 7.26 (t, J = 7.4, 1H, $H_{5'}$), 7.35 (d, J = 8.3, 1H, $H_{3'}$), 7.45-7.50 (m, 2H, $H_{4'}$, $H_{6'}$), 7.74 (d, J = 8.5, 2H, H_2, H_6), 7.89 (d, J = 8.6, 2H, H_3, H_5). ^{13}C-NMR (acetone-d_6, 125 MHz): δ 29.6 (CH_3), 101.8 (d, J = 215.6, CH_2F), 116.5 ($C_{3'}$), 124.7 ($C_{5'}$), 127.7 (C_3, C_5), 130.8 ($C_{4'}$), 131.0 (C_2, C_6), 131.1 ($C_{1'}$), 131.9 ($C_{6'}$), 139.4 (C_1), 142.9 (C_4), 154.5 (d, J = 3.0, $C_{2'}$). ^{19}F-NMR (acetone-d_6): δ –151.0. HPLC (Gradient-I, column C18, t_R, min): 15.99. MS (ESI, *m/z*, %): 296.1 ($[M + H]^+$, 100), 313.1 ($[M + NH_4]^+$, 81).

2-(Fluoromethoxy)-4'-(methanesulfonyl)-1,1'-biphenyl, 20. Following the general procedure for the monofluoroalkylation reaction, compound **20** was obtained from **33** (50 mg, 0.202 mmol) as a solid (55 mg, 97%). Chromatography: DCM.

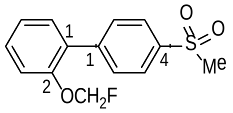

Mp: 117-119 ºC. R_f: 0.46 (DCM). IR (ATR): ν 1304, 1150 (SO_2). ^{1}H-NMR ($CDCl_3$): δ 3.12 (s, 3H, CH_3), 5.69 (d, J = 54.3, 2H, CH_2F), 7.24 (td, J = 7.5, 1.0, 1H, H_5), 7.28 (d, J = 8.1, 1H, H_3), 7.38 (dd, J = 7.6, 1.7, 1H, H_6), 7.44 (ddd, J = 8.1, 7.4, 1.8, 1H, H_4), 7.72 (d, J = 8.6 Hz, 2H, $H_{2'}$, $H_{6'}$), 8.00 (d, J = 8.5, 2H, $H_{3'}$, $H_{5'}$). ^{13}C-NMR ($CDCl_3$): δ 44.7 (CH_3), 101.1 (d, J = 220.0, CH_2F), 115.7 (d, J = 1.7, C_3), 124.1 (C_5), 127.2 ($C_{3'}$, $C_{5'}$), 130.0 (d, J = 1.1, C_1), 130.2 (C_4), 130.6 ($C_{2'}$, $C_{6'}$), 131.1 (C_6), 139.1 ($C_{4'}$), 143.6 ($C_{1'}$), 153.6 (d, J = 3.1, C_2). ^{19}F-NMR ($CDCl_3$): δ –149.1. HPLC (Gradient-I, column C18, t_R, min): 17.45. MS (ESI, *m/z*, %): 297.8 ($[M + NH_4]^+$, 100).

2-(Fluoromethoxy)-4'-(methanesulfonyl)-1,1'-biphenyl, 23. Following the general procedure for the monofluoroalkylation reaction, compound **23** was obtained from **37** (47 mg, 0.203 mmol) as an oil (47 mg, 87%). Chromatography: DCM.

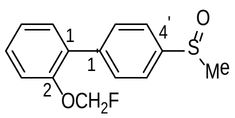

R_f: 0.44 (DCM/EtOAc 1:1). IR (ATR): ν 1083 (SO). ^{1}H-NMR (acetone-d_6): δ 2.75 (s, 3H, CH_3), 5.83 (d, J = 54.4, 2H, CH_2F), 7.24 (td, J = 7.5, 1.1, 1H, H_5), 7.34 (d, J = 7.5, 1H, H_3), 7.44 (d, J = 7.4, 1H, H_6), 7.46 (app td, J = 7.4, 1.7, 1H, H_4), 7.70-7.77 (m, 4H, $H_{2'}$, $H_{3'}$, $H_{5'}$, $H_{6'}$). ^{13}C-NMR (acetone-d_6): δ 44.2 (CH_3), 101.8 (d, J = 216.8, CH_2F), 116.4 (d,

J = 1.2, C_3), 124.1 ($C_{3'}$, $C_{5'}$), 124.6 (C_5), 130.4 (C_4), 131.1 ($C_{2'}$, $C_{6'}$), 131.5 (C_1), 131.9 (C_6), 141.2 ($C_{1'}$), 146.8 ($C_{4'}$), 154.5 (d, *J* = 3.3 Hz, C_2). ^{19}F-NMR (acetone-d_6): δ –150.8. HPLC (Gradient-I, column C18, t_R, min): 13.32. MS (ESI, *m/z*, %): 264.8 ($[M + H]^+$, 100).

4'-(Ethanesulfinyl)-2-(fluoromethoxy)-1,1'-biphenyl, 24. Following the general procedure for the monofluoroalkylation reaction, compound **24** was obtained from **38** (40 mg, 0.162 mmol) as an oil (24 mg, 52%). Chromatography: preparative TLC in toluene/DCM 95:5.

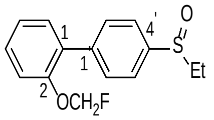

Rf: 0.31 (toluene/DCM 95:5). IR (ATR): ν 1084, 1048 (SO). ^{1}H-NMR (acetone-d_6, 500 MHz): δ 1.16 (t, *J* = 7.4, 3H, CH_3), 2.74-2.81 (m, 1H, 1/2CH_2), 2.97-3.04 (m, 1H, 1/2CH_2), 5.82 (d, *J* = 54.4, 2H, CH_2F), 7.24 (td, *J* = 7.4, 1.2 Hz, 1H, H_5), 7.34 (br dt, *J* = 8.7, 1.2, 1H, H_3), 7.43-7.46 (m, 2H, H_4, H_6), 7.69-7.73 (m, 4H, $H_{2'}$, $H_{3'}$, $H_{5'}$, $H_{6'}$). ^{13}C-NMR (acetone-d_6, 125 MHz): δ 5.95 (CH_3), 50.5 (CH_2), 101.9 (d, *J* = 215.4, CH_2F), 116.5 (d, *J* = 0.8, C_3), 124.6 (C_5), 124.7 ($C_{3'}$, $C_{5'}$), 130.4 (C_4), 131.0, ($C_{2'}$, $C_{6'}$), 131.5 (d, *J* = 0.8, C_1), 131.9 (C_6), 141.2 ($C_{1'}$), 144.3 ($C_{4'}$), 154.5 (d, *J* = 2.9, C_2). ^{19}F-NMR (acetone-d_6): δ –150.8. HPLC (Gradient-I, column C18, t_R, min): 13.10. MS (ESI, *m/z*, %): 279.1 ($[M + H]^+$, 100).

4'-(*S*-Ethanesulfonimidoyl)-2-(fluoromethoxy)-1,1'-biphenyl, 25. Following the general procedure for the monofluoroalkylation reaction, compound **25** was obtained from **40** (36 mg, 0.140 mmol) as a solid (34 mg, 84%). Chromatography: DCM.

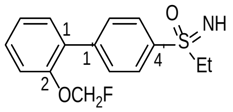

Mp: 92-93 ºC. *Rf*: 0.46 (DCM/methanol 95:5). IR (ATR): ν 3322 (NH), 1128, 1084 (SO). ^{1}H-NMR (acetone-d_6): δ 1.20 (t, *J* = 7.4, 3H, CH_3), 3.17 (q, *J* = 7.4, 2H, CH_2), 5.84 (d, *J* = 54.3, 2H, CH_2F), 7.26 (td, *J* = 7.9, 1.1, 1H, H_5), 7.35 (d, *J* = 8.0, 1H, H_3), 7.46 (d, *J* = 7.7, 1H, H_6), 7.47 (td, *J* = 7.3, 1.7, 1H, H_4), 7.74 (d, *J* = 8.5, 2H, $H_{2'}$, $H_{6'}$), 7.99 (d, *J* = 8.5, 2H, $H_{3'}$, $H_{5'}$). ^{13}C-NMR (acetone-d_6): δ 8.4 (CH_3), 52.4 (CH_2), 101.8 (d, *J* = 215.5, CH_2F), 116.4 (d, *J* = 1.1, C_3), 124.6 (C_5), 129.1 ($C_{3'}$, $C_{5'}$), 130.7 (C_4), 130.8 ($C_{2'}$, $C_{6'}$), 131.1 (d, *J* = 1.3, C_1), 131.9 (C_6), 141.9 ($C_{1'}$), 143.1 ($C_{4'}$), 154.5 (d, *J* = 3.0, C_2). ^{19}F-NMR (acetone-d_6): δ –151.0. HPLC (Gradient-I, column C18, t_R, min): 12.85. MS (ESI, *m/z*, %): 294.1 ($[M + H]^+$, 100).

4'-(*N*,*S*-Dimethanesulfonimidoyl)-2-(fluoromethoxy)-1,1'-biphenyl, 27. Following the general procedure for the monofluoroalkylation reaction, compound **27** was obtained from **44** (14 mg, 0.054 mmol) as an oil (15 mg, 95%). Chromatography: DCM.

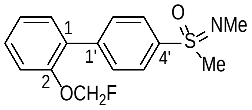

Rf: 0.45 (DCM/methanol 95:5). IR (ATR): ν 1151 (SO). ^{1}H-NMR (acetone-*d*6, 500 MHz): δ 2.57 (s, 3H, NCH_3), 3.08 (s, 3H, SCH_3), 5.85 (d, *J* = 54.3, 2H, CH_2F), 7.26 (td, *J* = 7.8, 1.2, 1H, H_5), 7.36 (dd, *J* = 7.6, 1.1, 1H, H_3), 7.46 (d, *J* = 7.5, 1H, H_6), 7.48 (app td, *J* = 7.3, 1.2, 1H, H_4), 7.77 (d, *J* = 8.6, 2H, $H_{2'}$, $H_{6'}$), 7.92 (d, *J* = 8.5, 2H, $H_{3'}$, $H_{5'}$). ^{13}C-NMR (acetone-*d*6, 125 MHz): δ 29.3 (NCH_3), 44.8 (SCH_3), 101.8 (d, *J* = 215.7, CH_2F), 116.4 (d, *J* = 1.2, C_3), 124.7 (C_5), 129.3 ($C_{3'}$, $C_{5'}$), 130.8 (C_4), 131.1 (d, *J* = 1.5, C_1), 131.2 ($C_{2'}$, $C_{6'}$), 131.9 (C_6), 139.4 ($C_{1'}$), 143.2 ($C_{4'}$), 154.5 (d, *J* = 3.0, C_2). ^{19}F-NMR (acetone-*d*6): δ −151.0. HPLC (Gradient-I, column C18, t_R, min): 15.31. MS (ESI, *m/z*, %): 294.1 ([M + H]$^+$, 100).

3'-Chloro-2-(fluoromethoxy)-4'-(*S*-methanesulfonimidoyl)-1,1'-biphenyl, 46. Following the general procedure for the monofluoroalkylation reaction, compound **46** was obtained from **54** (50 mg, 0.177 mmol) as an oil (45 mg, 80%). Chromatography: hexane to hexane/EtOAc 7:3.

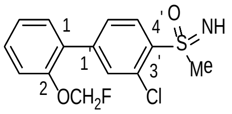

Rf: 0.41 (hexane/EtOAc 6:4). IR (ATR): ν 3273 (NH). ^{1}H-NMR (acetone-*d*6, 500 MHz): δ 3.28 (s, 3H, CH_3), 3.78 (br s, 1H, NH), 5.87 (d, *J* = 54.2, 2H, CH_2F), 7.26 (td, *J* = 7.5, 1.0, 1H, H_5), 7.36 (d, *J* = 8.4, 1H, H_3), 7.47 (dd, *J* = 7.7, 1.7, 1H, H_6), 7.50 (ddd, *J* = 8.0, 7.7, 1.8, 1H, H_4), 7.67 (dd, *J* = 8.2, 1.7, 1H, $H_{6'}$), 7.75 (d, *J* = 1.7, 1H, $H_{2'}$), 8.21 (d, *J* = 8.2, 1H, $H_{5'}$). ^{13}C-NMR (acetone-*d*6, 125 MHz): δ 44.1 (CH_3), 101.8 (d, *J* = 215.9, CH_2F), 116.4 (d, *J* = 1.4, C_3), 124.7 (C_5), 129.2 ($C_{6'}$), 129.6 (d, *J* = 1.3, C_1), 131.2 (C_4), 131.3 ($C_{5'}$), 131.8 (C_6), 132.3 ($C_{3'}$), 133.3 ($C_{2'}$), 141.5 ($C_{4'}$), 144.5 ($C_{1'}$), 154.4 (d, *J* = 2.9, C_2). ^{19}F-NMR (acetone-*d*6): δ −151.2. HPLC (Gradient-I, column C18, t_R, min): 15.05. MS (ESI, *m/z*, %): 314.0 ([M(^{35}Cl) + H]$^+$, 100), 316.0 ([M(^{37}Cl) + H]$^+$, 37).

2-Chloro-2'-(fluoromethoxy)-4-(*S*-methanesulfonimidoyl)-1,1'-biphenyl, 47. Following the general procedure for the monofluoroalkylation reaction, compound **47** was obtained from **55** (17 mg, 0.060 mmol) as an oil (18 mg, 96%). Chromatography: preparative TLC in DCM/methanol 98:2.

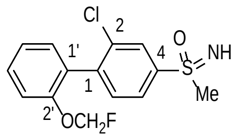

Rf: 0.41 (DCM/methanol 95:5). IR (ATR): ν 3269 (NH), 1217 (SO). ^{1}H-NMR (acetone-*d*$_6$, 700 MHz): δ 3.16 (s, 3H, CH_3), 3.63 (br s, 1H, NH), 5.79 (d, *J* = 54.3, 2H, CH_2F), 7.26 (td, *J* = 7.4, 0.8, 1H, $H_{5'}$), 7.30 (dd, *J* = 7.5, 1.8, 1H, $H_{6'}$), 7.35 (d, *J* = 8.3, 1H, $H_{3'}$), 7.52 (ddd, *J* = 8.5, 7.0, 2.2, 1H, $H_{4'}$), 7.58 (d, *J* = 8.0, 1H, H_6), 7.98 (dd, *J* = 8.0, 1.8, 1H, H_5), 8.07 (d, *J* = 1.8, 1H, H_3). ^{13}C-NMR (acetone-*d*$_6$, 175 MHz): δ 46.3 (CH_3), 101.8 (d, *J* = 215.7, CH_2F), 115.8 (d, *J* = 1.4 Hz, $C_{3'}$), 124.2 ($C_{5'}$), 126.8 (C_5), 129.2 (C_3), 130.0 ($C_{1'}$), 131.3 ($C_{4'}$), 131.7 ($C_{6'}$), 133.3 (C_6), 134.8 (C_2), 142.2 (C_1), 146.5 (C_4), 154.7 (d, *J* = 3.2 Hz, $C_{2'}$). ^{19}F-NMR (acetone-*d*$_6$): δ –150.5. HPLC (Gradient-I, column C18, t_R, min): 15.14. MS (ESI, *m/z*, %): 314.0 ([M(^{35}Cl) + H]$^+$, 100), 316.0 ([M(^{37}Cl) + H]$^+$, 38).

2.4. Synthesis and Characterization Data of Intermediate 15

1-Bromo-2-[(difluoromethyl)sulfanyl]benzene, 15. Following the general procedure for the difluoroalkylation reaction, compound **15** was obtained from 2-bromobenzenethiol (278 mg, 1.42 mmol) as an oil (275 mg, 81%). Chromatography: hexane. Spectroscopic data were in agreement with those reported.[6]

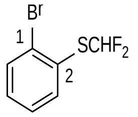

Rf: 0.50 (hexane/DCM 95:5). ^{1}H-NMR ($CDCl_3$): δ 6.92 (t, *J* = 56.9, 1H, CHF_2), 7.27 (td, *J* = 7.6, 1.8, 1H, H_5), 7.36 (td, *J* = 7.5, 1.6, 1H, H_4), 7.68 (dd, *J* = 6.4, 1.8, 1H, H_3), 7.70 (dd, *J* = 6.2, 1.6, 1H, H_6). ^{13}C-NMR ($CDCl_3$): δ 120.6 (t, *J* = 274.6, CHF_2), 128.4 (C_5), 128.5 (t, *J* = 3.1, C_2), 129.3 (t, *J* = 1.3, C_1), 131.1 (C_3), 133.9 (C_4), 136.6 (C_6). ^{19}F-NMR ($CDCl_3$): δ –92.5.

2.5. Synthesis and Characterization Data of Intermediates 33, 36, 39, 41, 43, 49, 50, 52, and 53 and Final Compounds 21 and 45

4'-(Methanesulfonyl)[1,1'-biphenyl]-2-ol, 33. Sulfide **32** (100 mg, 0.462 mmol) and $(NH_4)_6Mo_7O_{28}\cdot 4H_2O$ (70.2 mg, 0.046 mmol) were dissolved in anhydrous methanol (1.2 mL). The mixture was then cooled at 0 ºC and hydrogen peroxide (30%, 189 μL, 1.85 mmol) was added at a rate of 0.2 mL/min, mantaining the reaction temperature below 8 ºC. The reaction was stirred at 0 ºC for 30 min and then allowed to warm up to rt over 1

h. Next, the mixture was cooled to 0 ºC and sat. aqueous Na_2SO_3 (0.55 mL) was added dropwise so that the reaction temperature did not exceed 15 ºC. Water was added and the mixture was extracted with EtOAc (2x). The combined organic layers were dried over Na_2SO_4, filtered and concentrated under reduced pressure. The crude was purified by column chromatography from DCM to DCM/EtOAc 9:1 to afford sulfone **33** as a white solid (86 mg, 75%).

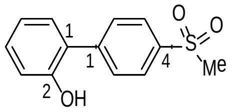

Mp: 148-149 ºC. R_f: 0.44 (DCM/EtOAc 9:1). IR (ATR): ν 3394 (OH), 1294, 1146 (SO_2). ^{1}H-NMR (acetone-d_6): δ 3.16 (s, 3H, CH_3), 6.98 (td, J = 7.5, 1.1, 1H, H_5), 7.04 (dd, J = 8.1, 0.9, 1H, H_3), 7.26 (ddd, J = 8.1, 7.5, 1.7, 1H, H_4), 7.38 (dd, J = 7.6, 1.7, 1H, H_6), 7.87 (d, J = 8.7, 2H, $H_{2'}$, $H_{6'}$), 7.97 (d, J = 8.6, 2H, $H_{3'}$, $H_{5'}$), 8.69 (s, 1H, OH). ^{13}C-NMR (acetone-d_6): δ 44.4 (CH_3), 117.2 (C_3), 121.1 (C_5), 127.4 (C_1), 127.8 ($C_{3'}$, $C_{5'}$), 130.6 (C_4), 130.9 ($C_{2'}$, $C_{6'}$), 131.5 (C_6), 140.3 ($C_{4'}$), 145.1 ($C_{1'}$), 155.2 (C_2). HPLC (Gradient-I, column C18, t_R, min): 21.31. MS (ESI, m/z, %): 246.8 ($[M - H]^-$, 100).

1-Bromo-4-(ethanesulfinyl)benzene, 36. To a solution of 1-bromo-4-(ethylsulfanyl)benzene (1.08 g, 4.97 mmol) in anhydrous DCM (70 mL), a solution of *m*CPBA (77%, 1.17 g, 5.22 mmol) in anhydrous DCM (25 mL) was added at a rate of 8 mL/min at 0 ºC, and the reaction was stirred for 30 min before warming up to rt. After 3 h, the mixture was filtered with a short pad of silica gel, and washed with a saturated aqueous solution of $NaHCO_3$. Next, the aqueous layer was extracted with DCM (x3), and the combined organic layers were washed with water, dried over Na_2SO_4, concentrated under reduced pressure. The residue was purified by column chromatography (hexane to hexane/EtOAc 7:3) to afford sulfoxide **36** as an oil (1.06 g, 92%). Spectroscopic data were in agreement with those reported. [7]

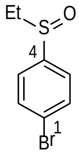

R_f: 0.36 (hexane/EtOAc 2:8). IR (ATR): ν 1313, 1145 (SO). ^{1}H-NMR ($CDCl_3$): δ 1.97 (t, J = 7.4, 3H, CH_3), 2.68-2.80 (m, 1H, 1/2CH_2), 2.85-2.96 (m, 1H, 1/2CH_2), 7.48 (d, J = 8.5, 2H, H_2, H_6), 7.66 (d, J = 8.3, 2H, H_3, H_5). ^{13}C-NMR ($CDCl_3$): δ 7.6 (CH_3), 50.8 (CH_2), 129.2 (C_1), 129.4 (C_3, C_5), 132.7 (C_2, C_6), 137.8 (C_4). MS (ESI, m/z, %): 233.0 ($[M(^{79}Br) + H]^+$, 100), 235.0 ($[M(^{81}Br) + H]^+$, 100).

1-Bromo-4-(*S*-ethanesulfonimidoyl)benzene, 39. A solution of sulfoxide **36** (635 mg, 2.72 mmol) and sodium azide (212 mg, 3.26 mmol) in anhydrous $CHCl_3$ (2.7 mL) was stirred in a pre-dried three neck round-bottom flask equipped with a reflux condenser and an addition funnel. Next, concentrated H_2SO_4 (0.70 mL, 12.5 mmol) was added dropwise at 0 ºC. The resulting mixture was then slowly warmed up to 45 ºC and maintained overnight with magnetic stirring. After that time, the reaction was cooled and ice water was added. When all the salts were dissolved, the organic layer was separated and the aqueous layer was extracted with DCM first, made slightly alkaline with 20% aqueous NaOH and re-extracted with DCM (3x). The combined organic extracts were dried over Na_2SO_4, filtered, and concentrated under reduced pressure. The resulting residue was purified by column chromatography from DCM to DCM/methanol 96:4 to afford the desired sulfoximine **39** as an oil (620 mg, 92%).

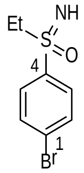

R_f: 0.30 (DCM/methanol 95:5). IR (ATR): ν 3261 (NH), 1213 (SO). ^{1}H-NMR ($CDCl_3$): δ 1.26 (t, J = 7.4, 3H, CH_3), 2.68 (br s, 1H, NH), 3.16 (q, J = 7.4, 2H, CH_2), 7.69 (d, J = 8.7, 2H, H_2, H_6), 7.83 (d, J = 8.7, 2H, H_3, H_5). ^{13}C-NMR ($CDCl_3$): δ 8.0 (CH_3), 52.0 (CH_2), 128.5 (C_1), 130.3 (C_3, C_5), 132.6 (C_2, C_6), 140.7 (C_4). HPLC (Gradient-I, column C18, t_R, min): 14.01. MS (ESI, *m/z*, %): 248.0 ([M(^{79}Br) + H]$^+$, 99), 250.0 ([M(^{81}Br) + H]$^+$, 100).

NH-sulfoximines 41, 52, 53. General procedure. (i) *N*-bromosuccinimide (1.5 equiv) was added to a solution of the corresponding (methylsulfanyl)arene (1.0 equiv), potassium *tert*-butoxide (1.2 equiv) and cyanamide (1.3 equiv) in anhydrous methanol (5 mL/mmol) and the reaction mixture was stirred at rt for 1.5 h. Next, the solvent was evaporated under reduced pressure and the resulting residue was partitioned between DCM and water. The aqueous phase was extracted with DCM (x2), and the combined organic layers were dried over Na_2SO_4, filtered and the solvent was evaporated under reduced pressure. The crude *N*-cyanosulfylimine was used in the next step without further purification.

ii) *N*-cyanosulfylimine was dissolved in a 1:1 mixture of anhydrous ACN/DCM (5 mL/mmol), $RuCl_3$ (0.01 equiv) was added and the resulting brown mixture was stirred at rt for 5 min. Then, a 0.15 M solution of $NaIO_4$ (1.50 equiv) in water was added and the

reaction was stirred at rt until completion (TLC). Next, water was added and the mixture was extracted with DCM (x3). The combined organic layers were washed with sat. aqueous $Na_2S_2O_3$ (x2) and brine, dried over Na_2SO_4, filtered and concentrated under reduced pressure. The crude *N*-cyanosulfoximine was used in the next step without further purification.

iii) To the corresponding *N*-cyanosulfoximine above synthesized, 50% aqueous H_2SO_4 (4.5 mL/mmol) was added and the mixture was heated at reflux for 2 h and then allowed to cool to rt. Next, neutralization at 0 ºC was performed with 50% aqueous NaOH and the reaction was extracted with DCM (x3). The combined organic layers were dried over Na_2SO_4, filtered and the solvent was evaporated under reduced pressure. The crude residue was purified by column chromatography to afford the desired NH-sulfoximine.

1-Bromo-4-(*S*-methanesulfonimidoyl)benzene, 41. Following the previous general procedure, [(4-bromophenyl)(methyl)-λ^4-sulfanylidene]cyanamide was obtained from 1-bromo-4-(methylsulfanyl)benzene (0.69 g, 3.39 mmol)) as a solid (0.82 mg, quantitative).

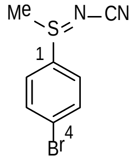

Rf: 0.33 (hexane/EtOAc 1:1). ^{1}H-NMR ($CDCl_3$): δ 3.02 (s, 3H, CH_3), 7.67 (d, *J* = 8.8, 2H, H_3, H_5), 7.76 (d, *J* = 8.8, 2H, H_2, H_6).

Next, [(4-bromophenyl)(methyl)-λ^4-sulfanylidene]cyanamide above synthesized (0.71 g, 2.91 mmol) was transformed into [(4-bromophenyl)(methyl)oxo-λ^6-sulfanylidene]cyanamide (solid, 0.63 g, 83%).

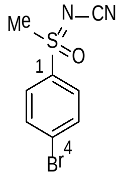

Rf: 0.30 (hexane/EtOAc 1:1). ^{1}H-NMR ($CDCl_3$): δ 3.34 (s, 3H, CH_3), 7.81-7.88 (m, 4H, $4CH_{Ar}$).

Finally, compound **41** was obtained from [(4-bromophenyl)(methyl)oxo-λ^6-sulfanylidene]cyanamide (1.20 g, 4.63 mmol) as a white solid (0.83 mg, 77%). Chromatography: hexane to hexane/EtOAc 1:1. Spectroscopic data were in agreement with those reported.[8]

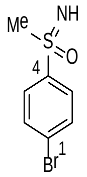

Mp: 88-89 ºC. R_f: 0.31 (hexane/EtOAc 2:8). IR (ATR): ν 3288 (NH), 1387, 1221 (SO). ^{1}H-NMR (acetone-d_6): δ 3.06-3.07 (m, 3H, CH_3), 7.79 (d, J = 8.7, 2H, H_2, H_6), 7.92 (d, J = 8.7, 2H, H_3, H_5). ^{13}C-NMR (acetone-d_6): δ 46.5 (CH_3), 127.6 (C_1), 130.6 (C_3, C_5), 133.0 (C_2, C_6), 145.0 (C_4). MS (ESI, *m/z*, %): 233.7 ([M(^{79}Br) + H]$^+$, 99), 235.7 ([M(^{81}Br) + H]$^+$, 100).

4-Bromo-2-chloro-1-(*S*-methanesulfonimidoyl)benzene, 52. Following the previous general procedure, [(4-bromo-2-chlorophenyl)(methyl)-λ^4-sulfanylidene]cyanamide was obtained from **49** (346 mg, 1.45 mmol) as a solid (402 mg, quantitative).

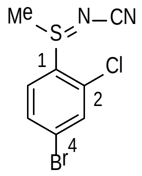

R_f: 0.35 (hexane/EtOAc 1:1). ^{1}H-NMR ($CDCl_3$): δ 3.01 (s, 3H, CH_3), 7.70 (d, J = 1.8, 1H, H_3), 7.75 (dd, J = 8.5, 1.9, 1H, H_5), 8.00 (d, J = 8.5, 1H, H_6).

Next, [(4-bromo-2-chlorophenyl)(methyl)-λ^4-sulfanylidene]cyanamide above synthesized (380 mg, 1.37 mmol) was transformed into [(4-bromo-2-chlorophenyl)(methyl)oxo-λ^6-sulfanylidene]cyanamide (solid, 338 mg, 84%).

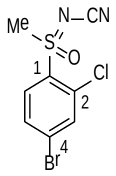

R_f: 0.34 (hexane/EtOAc 1:1). ^{1}H-NMR ($CDCl_3$): δ 3.53 (s, 3H, CH_3), 7.74 (dd, J = 8.6, 1.9, 1H, H_5), 7.85 (d, J = 1.8, 1H, H_3), 8.06 (d, J = 8.6, 1H, H_6).

Finally, compound **52** was obtained from [(4-bromo-2-chlorophenyl)(methyl)oxo-λ^6-sulfanylidene]cyanamide (70 mg, 0.238 mmol) as a white solid (33 mg, 51%). Chromatography: hexane to hexane/EtOAc 6:4.

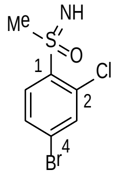

Mp: 82-83 ºC. R_f: 0.28 (hexane/EtOAc 1:1). IR (ATR): ν 3275 (NH), 1227 (SO). ^{1}H-NMR ($CDCl_3$): δ 3.30 (s, 3H, CH_3), 7.60 (dd, J = 8.5, 1.9, 1H, H_5), 7.72 (d, J = 1.8, 1H,

H_3), 8.05 (d, *J* = 8.5, 1H, H_6). ^{13}C-NMR ($CDCl_3$): δ 43.6 (CH_3), 128.2 (C_4), 130.8, 132.0 (C_3, C_5), 133.5 (C_2), 134.7 (C_6), 140.3 (C_1). HPLC (Gradient-I, column C18, t_R, min): 14.20. MS (ESI, *m/z*, %): 267.8 ([M(^{79}Br, ^{35}Cl) + H]$^+$, 76), 269.8 ([M(^{79}Br, ^{37}Cl) + H]$^+$, 100), 271.9 ([M(^{81}Br, ^{37}Cl) + H]$^+$, 28).

1-Bromo-2-chloro-4-(*S*-methanesulfonimidoyl)benzene, 53. Following the previous general procedure, [(4-bromo-3-chlorophenyl)(methyl)-λ^4-sulfanylidene]cyanamide was obtained from **50** (248 mg, 1.04 mmol) as a solid (289 mg, quantitative).

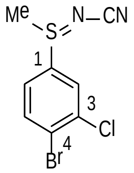

Rf: 0.32 (hexane/EtOAc 1:1). ^{1}H-NMR ($CDCl_3$): δ 3.03 (s, 3H, CH_3), 7.55 (dd, *J* = 8.4, 2.2, 1H, H_6), 7.86 (d, *J* = 2.2, 1H, H_2), 7.88 (d, *J* = 8.4, 1H, H_5).

Next, [(4-bromo-3-chlorophenyl)(methyl)-λ^4-sulfanylidene]cyanamide above synthesized (280 mg, 1.01 mmol) was transformed into [(4-bromo-3-chlorophenyl)(methyl)oxo-λ^6-sulfanylidene]cyanamide (solid, 264 mg, 89%).

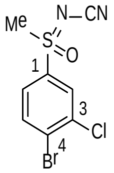

Rf: 0.32 (hexane/EtOAc 1:1). ^{1}H-NMR ($CDCl_3$): δ 3.36 (s, 3H, CH_3), 7.73 (dd, *J* = 8.5, 2.2, 1H, H_6), 7.96 (d, *J* = 8.5, 1H, H_5), 8.06 (d, *J* = 2.2, 1H, H_2).

Finally, compound **53** was obtained from [(4-bromo-3-chlorophenyl)(methyl)oxo-λ^6-sulfanylidene]cyanamide (278 mg, 0.947 mmol) as a white solid (119 mg, 46%). Chromatography: hexane to hexane/EtOAc 6:4.

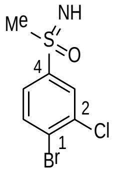

Mp: 110-111 ºC. *Rf*: 0.27 (hexane/EtOAc 1:1). IR (ATR): ν 3275 (NH), 1227 (SO). ^{1}H-NMR ($CDCl_3$): δ 3.11 (s, 3H, CH_3), 7.74 (dd, *J* = 8.4, 2.1, 1H, H_5), 7.76 (d, *J* = 8.3, 1H, H_6), 8.08 (d, *J* = 2.1, 1H, H_3). ^{13}C-NMR ($CDCl_3$): δ 46.2 (CH_3), 126.9 (C_5), 128.6 (C_1), 129.7 (C_3), 134.8 (C_6), 136.1 (C_2), 144.4 (C_4). HPLC (Gradient-I, column C18, t_R, min): 13.79. MS (ESI, *m/z*, %): 267.8 ([M(^{79}Br, ^{35}Cl) + H]$^+$, 75), 269.8 ([M(^{79}Br, ^{37}Cl) + H]$^+$, 100), 271.9 ([M(^{81}Br, ^{37}Cl) + H]$^+$, 27).

1-Bromo-4-(*N,S*-dimethanesulfonimidoyl)benzene, 43. A mixture of **41** (103 mg, 0.442 mmol), formaldehyde (37% in water, 1.92 mL) and formic acid (3.54 mL) was heated in an open flask at 100 ºC for 2 days. After evaporating the volatile substances under reduced pressure, the aqueous phase was neutralized using solid $NaHCO_3$ and extracted with DCM (x3). The combined organic layers were dried over Na_2SO_4, filtered, and concentrated under reduced pressure to afford compound **43** as an oil (110 mg, quantitative), which was used in the next step without further purification. Spectroscopic data were in agreement with those reported.[9]

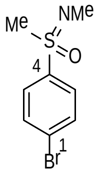

Rf: 0.41 (DCM/methanol 95:5). IR (ATR): ν 1242, 1151 (SO). ^{1}H-NMR (acetone-*d*$_6$): δ 2.54 (s, 3H, NCH_3), 3.15 (s, 3H, SCH_3), 7.84 (m, 4H, 4CH_{Ar}). ^{13}C-NMR (acetone-*d*$_6$): δ 29.2 (NCH_3), 44.4 (SCH_3), 128.1 (C_1), 131.6 (C_3, C_5), 133.5 (C_2, C_6), 139.5 (C_4). MS (ESI, *m/z*, %): 248.0 ([M(^{79}Br) + H]$^+$, 99), 250.0 ([M(^{81}Br) + H]$^+$, 100).

(Methylsulfanyl)benzenes 49, 50. General procedure. A mixture of the corresponding benzenethiol derivative (1.0 equiv), methyliodide (1.1 equiv) and K_2CO_3 (1.2 equiv) in anhydrous acetone (1.5 mL/mmol) was stirred at rt for 5 h. Next, the reaction was poured into water and extracted with EtOAc (x3). The combined organic layers were washed with brine, dried over Na_2SO_4, filtered and concentrated under reduced pressure. The crude product was purified by column chromatography, if necessary.

4-Bromo-2-chloro-1-(methylsulfanyl)benzene, 49. Following the previous general procedure, compound **49** was obtained from 4-bromo-2-chlorobenzenethiol (520 mg, 2.32 mmol) as an oil (575 mg, quantitative), which was used in the next step without further purification.

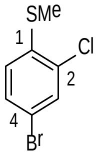

Rf: 0.46 (hexane/DCM 9:1). ^{1}H-NMR ($CDCl_3$): δ 2.46 (s, 3H, CH_3), 7.00 (d, *J* = 8.5, 1H, H_6), 7.36 (dd, *J* = 8.5, 2.1, 1H, H_5), 7.49 (d, *J* = 2.1, 1H, H_3). ^{13}C-NMR ($CDCl_3$): δ 15.4 (CH_3), 118.0 (C_4), 126.7 (C_6), 130.4, 132.0 (C_3, C_5), 132.6 (C_2), 137.3 (C_1).

1-Bromo-2-chloro-4-(methylsulfanyl)benzene, 50. Following the previous general procedure, compound **50** was obtained from **51** (980 mg, 4.38 mmol) as an oil (586 mg, 56%). Chromatography: hexane.

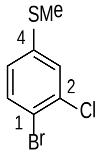

Rf: 0.42 (hexane/DCM 95:5). ¹H-NMR ($CDCl_3$): δ 2.47 (s, 3H, CH_3), 6.98 (dd, *J* = 8.4, 2.2, 1H, H_5), 7.29 (d, *J* = 2.2, 1H, H_3), 7.48 (d, *J* = 8.4, 1H, H_6). ¹³C-NMR ($CDCl_3$): δ 15.8 (CH_3), 118.4 (C_1), 126.0 (C_5), 127.6 (C_3), 133.7 (C_6), 135.0 (C_2), 140.0 (C_4).

2'-(Fluoromethoxy)[1,1'-biphenyl]-4-carboxylic acid, 21. Compound **17** (39 mg, 0.150 mmol) was dissolved in a solution of NaOH (62 mg, 1.50 mmol) in a 1:1 mixture of THF/water (0.45 mL) and the reaction was stirred at rt for 12 h. Next, pH was adjusted to 1 with saturated aqueous solution of $NaHSO_4$ and the mixture was diluted with brine. The product was extracted with EtOAc (x3) and the combined organic layers were dried over Na_2SO_4, filtered and concentrated under reduced pressure to afford the final product **21** as a white solid (37 mg, quantitative).

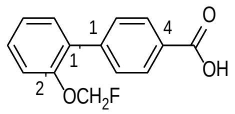

Mp: 197-198 ºC. *Rf*: 0.20 (hexane/EtOAc 6:4). IR (ATR): ν 1675 (CO). ¹H-NMR (acetone-*d*6): δ 5.83 (d, *J* = 54.5, 2H, CH_2F), 7.26 (td, *J* = 7.5, 1.1, 1H, $H_{5'}$), 7.36 (d, *J* = 8.7, 1H, $H_{3'}$), 7.45 (d, *J* = 7.2, 1H, $H_{6'}$), 7.44-7.49 (m, 1H, $H_{4'}$), 7.66 (d, *J* = 8.4, 2H, H_2, H_6), 8.10 (d, *J* = 8.4, 2H, H_3, H_5). ¹³C-NMR (acetone-*d*6): δ 101.4 (d, *J* = 216.7, CH_2F), 116.1 (d, *J* = 1.1, $C_{3'}$), 124.2 ($C_{5'}$), 129.6 ($C_{1'}$), 129.7 (C_3, C_5), 130.0 (C_2, C_6), 130.1 ($C_{4'}$), 131.2 (C_4), 131.3 ($C_{6'}$), 143.0 (C_1), 154.0 (d, *J* = 3.2, $C_{2'}$), 167.0 (COOH). ¹⁹F-NMR (acetone-*d*6): δ –150.8. HPLC (Gradient-IV, column C18, t_R, min): 28.20. MS (ESI, *m/z*, %): 245.1 ([M - H]⁻, 100).

3-(Fluoromethoxy)-2-[4-(*S*-methanesulfonimidoyl)phenyl]pyridine, 45. A round-bottom flask was charged with **41** (55 mg, 0.230 mmol), bis(pinacolato)diboron (63 mg, 0.250 mmol), potassium acetate (39 mg, 0.400 mmol) and dicyclohexyl(2',6'-dimethoxybiphenyl-2-yl)phosphane (SPhos) (7 mg, 0.020 mmol) and the system was evacuated and back-filled with argon (x3) before adding anhydrous 1,4-dioxane (0.8 mL). Next, $Pd_2(dba)_3 \cdot CHCl_3$ (2.5 mg, 0.002 mmol) was added and the reaction was heated at 110 ºC overnight. After consumption of starting material (TLC and ¹H-NMR), a solution

of **48** (39 mg, 0.190 mmol) in anhydrous 1,4-dioxane (0.2 mL), 5 M aqueous K_3PO_4 (233 mg, 1.100 mmol) and another charge of catalyst were added at rt and the reaction was heated at 110 ºC overnight. Next, the reaction was cooled to rt, filtered through Celite and the filtrate was concentrated under reduced pressure. The residue was purified by preparative TLC in DCM/methanol 95:5 to afford the desired final product as a white solid (16 mg, 24%).

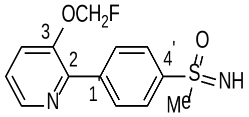

Mp: 131-132 ºC. *Rf*: 0.19 (DCM/methanol 95:5). IR (ATR): ν 3262 (NH), 1223, 1198 (SO). ^{1}H-NMR (acetone-d_6, 500 MHz): δ 3.10 (CH_3), 5.92 (d, *J* = 53.8, 2H, CH_2F), 7.48 (dd, *J* = 8.4, 4.6, 1H, H_5), 7.78 (dt, *J* = 8.4, 1.1, 1H, H_4), 8.06 (d, *J* = 8.6, 2H, $H_{2'}$, $H_{6'}$), 8.13 (d, *J* = 8.6, 2H, $H_{3'}$, $H_{5'}$), 8.49 (dd, *J* = 4.6, 1.2, 1H, H_6). ^{13}C-NMR (acetone-d_6, 125 MHz): δ 46.6 (CH_3), 101.7 (d, *J* = 217.1, CH_2F), 124.2 (d, *J* = 1.1, C_4), 125.2 (C_5), 128.0 ($C_{2'}$, $C_{6'}$), 130.8 ($C_{3'}$, $C_{5'}$), 142.3 ($C_{1'}$), 145.2 ($C_{4'}$), 145.3 (C_6), 147.6 (d, *J* = 0.7, C_2), 151.9 (d, *J* = 2.8, C_3). ^{19}F-NMR (acetone-d_6): δ −152.4. HPLC (Gradient-I, column C18, t_R, min): 12.95. MS (ESI, *m/z*, %): 281.1 ($[M + H]^+$, 100).

3. ADMET Assays

HSA binding assay. The assessment of compounds binding to HSA was performed by incubating a fixed concentration of the compound with different concentrations of immobilized HSA, using the TRANSILXL HSA Binding Kit (TMP-0210-2096, Sovicell). An 8-well unit of the TRANSIL assay plate was used for each compound: six wells contain increasing concentrations of HSA immobilized on silica beads suspended in PBS at pH 7.4, and two wells contain buffer only and serve as references to account for nonspecific binding. The TRANSIL assay plate was thawed for 3 h at rt and centrifuged at 750*g* for 5 s. Then, 15 µL of an 80 µM stock solution of the compound in PBS (for a final concentration of 5 µM) were added to each well of the 8-well unit, and the plate was incubated on a plate shaker at 1000 rpm for 12 min at rt. After this time, the plate was centrifuged at 750*g* for 10 min, and 50 µL of the supernatants were transferred for analytical quantification by HPLC-MS using selected ion monitoring (SIM). The binding percentage was calculated from the remaining free compound concentration in the supernatant of each well, using the spreadsheet and algorithms supplied with the kit.

Stability in mouse serum. Stability for selected compounds and reference compound enalapril was assayed by adding 625 µL of a 250 µM solution of test compound in PBS pH 7.4 to 1.875 mL of mouse serum (Europa Bioproducts, EQSM-0100) pre-warmed at 37 ºC. Next, solutions were incubated at 37 ºC for 4 h, taking aliquots of 250 µL at t = 0, 1, 2, 3, and 4 h. Each aliquot was quenched in 375 µL of cold ACN, vortexed, incubated for 10 min in ice and centrifuged at 39000*g* for 10 min. Supernatants were then analyzed by HPLC-MS using SIM mode, and quantification was estimated by using the peak area integration normalized with an internal standard.

Stability assay in mouse liver homogenate. Stability of test compounds and reference compound propranolol was determined by incubation in liver homogenates following previously reported procedure.[10] Livers from male C57BL6/J mice weighing 20-24 g (Charles Rivers Laboratories, L'Arbresle Cedex, France) were weighed, cut into small pieces and transferred to a glass dounce homogenizer, keeping the tissues at 0 ºC during the whole procedure. Next, 4 mL of ice-cold incubation buffer (50 mM Tris-HCl, pH 7.4) were added per gram of tissue, and the mixtures were first dounced with a Teflon pestle, and then homogenized 3 times using a Teflon digital homogenizer (15 s each time). The solutions were allowed to cool on ice between each homogenation step. The homogenates were centrifuged at 2000*g* for 10 min at 4 ºC, and the supernatants were stored at -80 ºC until biochemical assay. Prior to the experiment, the total protein concentration in the

tissue homogenates was determined using the Folin-based DC Protein Assay kit (500-0112, Bio-Rad) with bovine serum albumin as a reference standard.

To perform metabolic stability assay, liver homogenates were diluted to 2.5 mg protein/mL using ice-cold incubation buffer (50 mM Tris-HCl, pH 7.4). The metabolic reaction was initiated by addition of a 50 μM solution of test compound in 50 mM Tris-HCl pH 7.4 (for a final concentration of 10 μM), and the mixtures were vortexed and incubated at 37 ºC. Aliquots (50 μL) were withdrawn at times t = 0, 1, 4, 8, and 24 h (t = 0, 5, 15, 3, and 60 min in the case of propranolol), and mixed with 3 volumes of ice-cold ACN to terminate the enzymatic activity. Samples were vortexed, centrifuged at 10000*g* for 10 min at 4 ºC, and the resulting supernatants were analyzed by HPLC-MS using SIM mode and quantification was estimated by using the peak area integration normalized with an internal standard.

Parallel artificial membrane permeability assay (PAMPA). The assessment of the membrane permeability of selected compounds and reference compounds propranolol and metoprolol was performed in a commercially available 96-well Corning Gentest pre-coated PAMPA plate system (Cultek S.L.U., Spain). Prior to use, the pre-coated PAMPA plate system was warmed to rt for 30 min and 300 μL of 200 μM solution of tested compound in 2% DMSO in PBS were added to the wells in the receiver (donor) plate. Then, 200 μL of PBS were added to the wells in the filter (acceptor) plate. The filter plate was placed on the receiver plate by slowly lowering the pre-coated PAMPA plate until it sits on the receiver plate. The assembly was incubated at rt for 5 h, and then buffer samples were collected carefully from each plate. The final concentrations of compound in both donor and acceptor wells were analyzed by HPLC-MS using SIM mode, and quantification was estimated by using the peak area integration normalized with an internal standard. Permeability value of the compounds was calculated using the following formula: $P\ (cm/s) = \{-\ln[1-C_A(t)/C_{eq}]\}/[A*(1/V_D+1/V_A)*t]$, where A = filter area (0.3 cm^2), V_D = donor well volume (0.3 mL), V_A = acceptor well volume (0.2 mL), t = incubation time (s), $C_A(t)$ = compound concentration (μM) in acceptor well at time t, $C_D(t)$ = compound concentration (μM) in donor well at time t, and $C_{eq} = [C_D(t)*V_D+C_A(t)*V_A]/(V_D+V_A)$.

Solubility. Stock solutions (10^{-2} M) of the assayed compounds were diluted to concentrations ranging from 300 to 0.1 µM in a transparent 384-well plate (Greiner 781801) with 1% DMSO: 99% PBS buffer. The solutions were incubated for 2 h at 37 ºC and read in a NEPHELOstar Plus instrument (BMG LABTECH). The results were

adjusted to a segmented regression to obtain the maximum concentration in which compound is soluble.

Cytochrome inhibition. The objective of this study was to screen the potential of test compound to reversibly inhibit human P450 cytochrome (CYP) enzymes, and was performed using recombinant isoforms CYP1A2, CYP2C9, CYP2C19, CYP2D6, and CYP3A4, and probe substrates with fluorescent detection.

Assays were conducted in a 200 µL volume in 96-well microtiter plates (COSTAR 3915). Addition of cofactor-buffer mixture (KH_2PO_4 buffer, 1.3 mM NADP, 3.3 mM $MgCl_2$, 3.3 mM glucose-6-phosphate, and 0.4 U/mL glucose-6-phosphate dehydrogenase), supersomes control, standard inhibitors (furafyline, tranylzypromine, ketoconazole, sulfaphenazole, and quinidine from Sigma Aldrich) and test compound to the plate was carried out by a liquid handling station (Zephyr Caliper). The plate was then pre-incubated at 37 ºC for 5 min, and the reaction was initiated by the addition of the pre-warmed enzyme/substrate (E/S) mix. The E/S mix contained KH_2PO_4 buffer, c-DNA-expressing P450 in insect cell microsomes, substrate (3-cyano-7-ethoxycoumarin for CYP1A2 and CYP2C19, 7-methoxy-4(trifluoromethyl)coumarin for CYP2C9, 3-[2-(*N*,*N*-diethyl-*N*-methylammonium)ethyl]-7-methoxy-4-methylcoumarin for CYP2D6, and 7-benzyloxytrifluoromethylcoumarin for CYP3A4), to give the final assay concentrations in a reaction volume of 200 µL. Reactions were terminated after specific time for each cytochrome by addition of STOP solution (0.5 M 80:20 ACN/TrisHCl). Fluorescence per well was measured using a fluorescence plate reader (Tecan Infinite M1000 pro) and percentage of inhibition was calculated.

hERG ion channel inhibition. The assay was carried out using a CHO cell line transfected with the hERG potassium channel. Cells were seeded (2500 cells/well) 72 h before the assay on a 384-well black plate (Greiner 781091). Cells were maintained at 37 ºC in a 5% CO_2 atmosphere for 24 h and at 30 ºC in a 5% CO_2 atmosphere for 48 h. hERG activity was measured by using the Fluxor^{TM} Potassium Ion Chanel Assay Kit (Thermo Fisher F10016). Medium was replaced for 20 µL of loading buffer and cells were incubated for 60 min at 25 ºC, protected from direct light. After incubation, loading buffer was replaced for assay buffer and compounds were incubated for 30 min at 25 ºC. After this time, 5 µL of stimulus buffer was added to each well and fluorescence was read (λ_{ex} = 490 nm, λ_{em} = 525 nm) every second after the establishment of a baseline line using an imaging plate reader system (FDSS7000EX, Hamamatsu®). The fluorescent signal was

normalized to the maximum inhibition shown by controls and percentage of inhibition was calculated.

4. NMR Spectra of Compounds 3 and 26

^{1}H NMR spectrum for **3** ($CDCl_3$, 300 MHz)

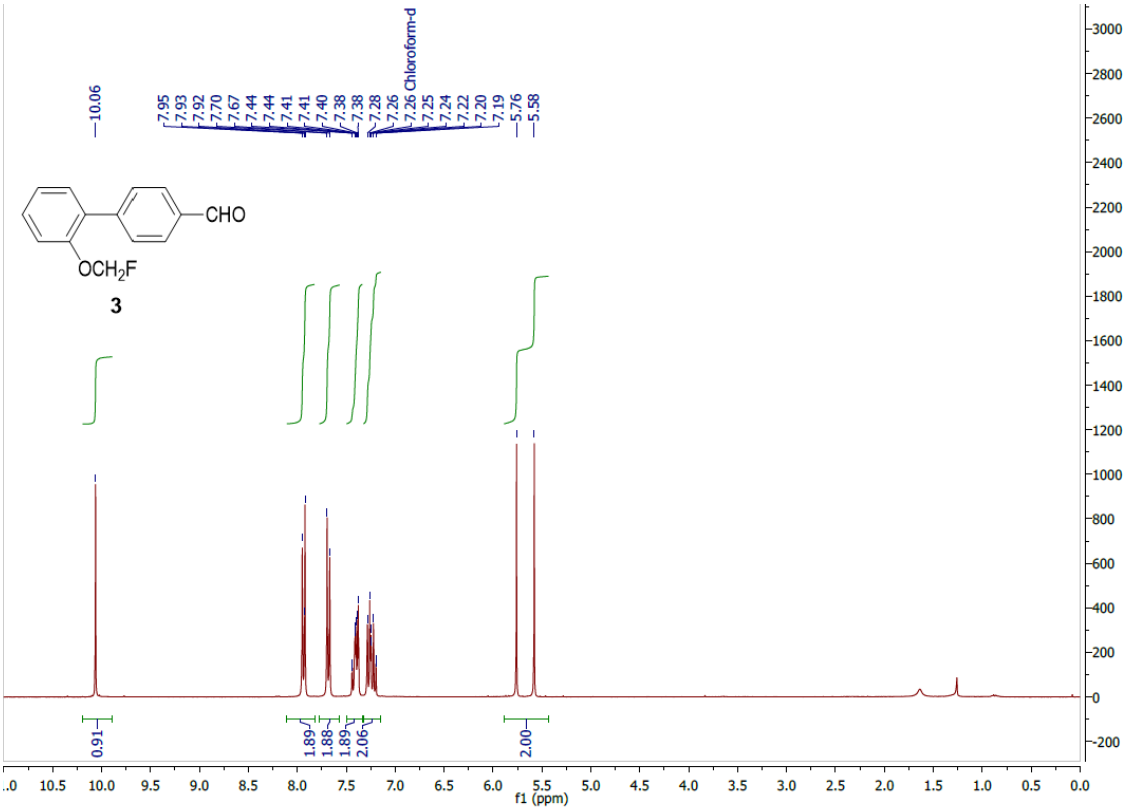

^{13}C NMR spectrum for **3** ($CDCl_3$, 75 MHz)

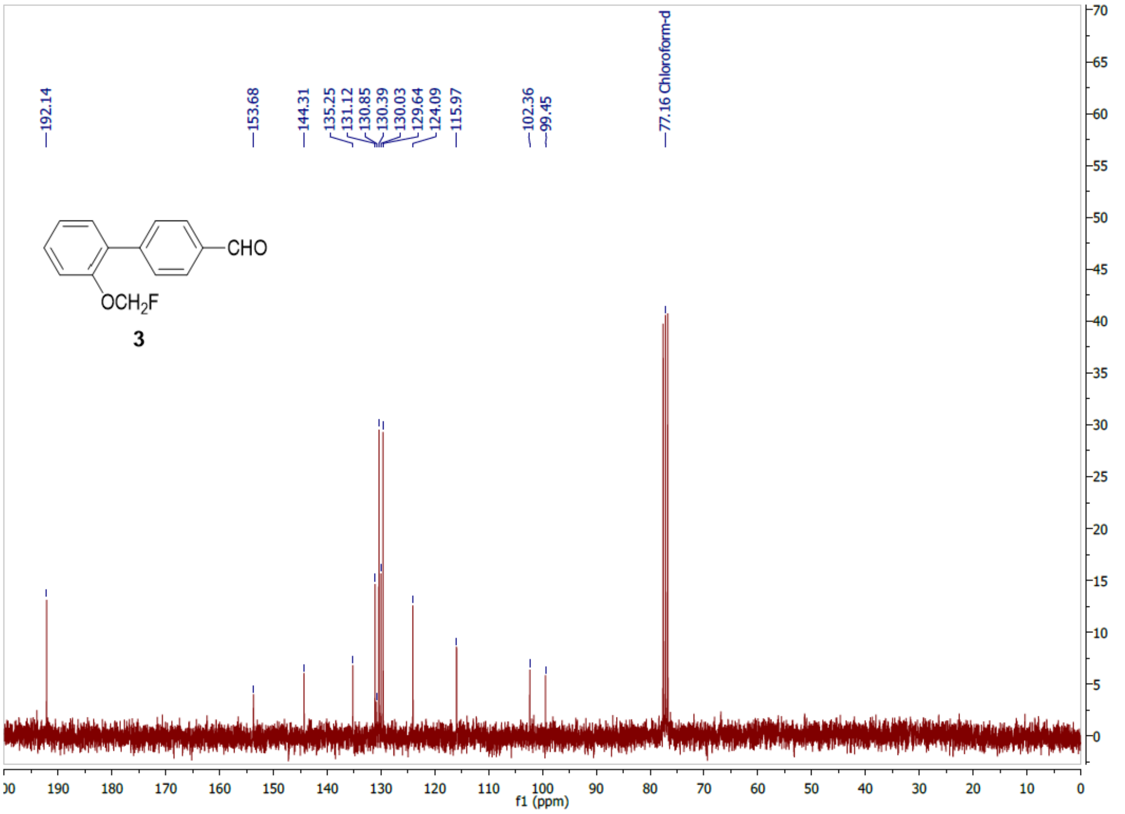

^{1}H NMR spectrum for **26** (acetone-d_6, 300 MHz)

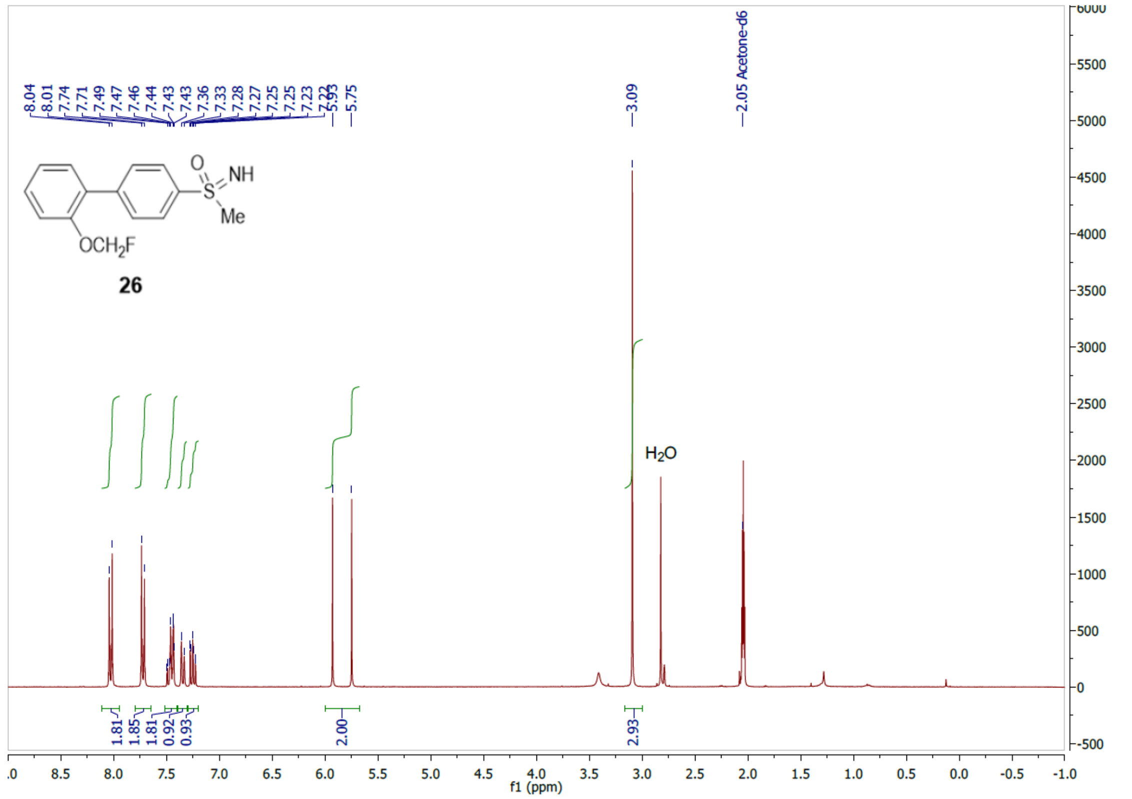

^{13}C NMR spectrum for **26** (acetone-d_6, 125 MHz)

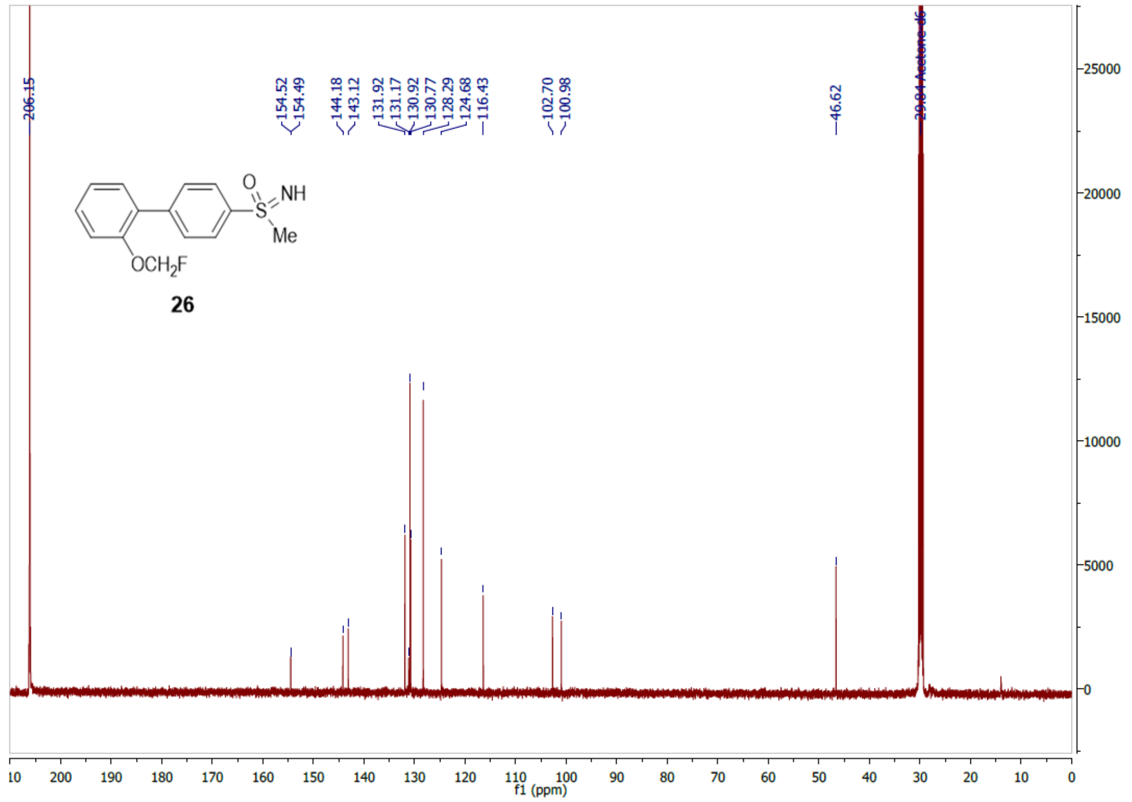

5. HPLC Traces of Compounds 3, and *rac*, (*S*)-, (*R*)-26

Compound **3**

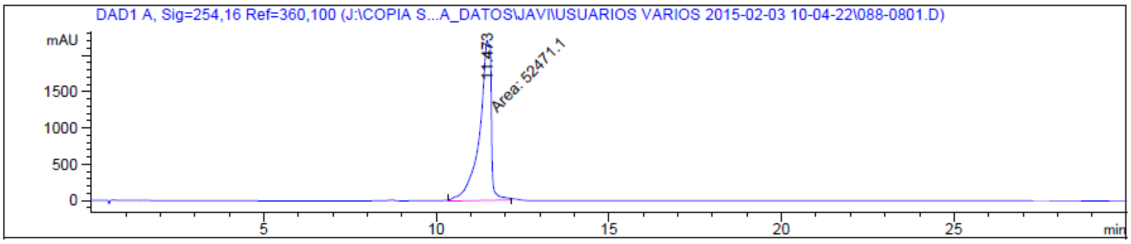

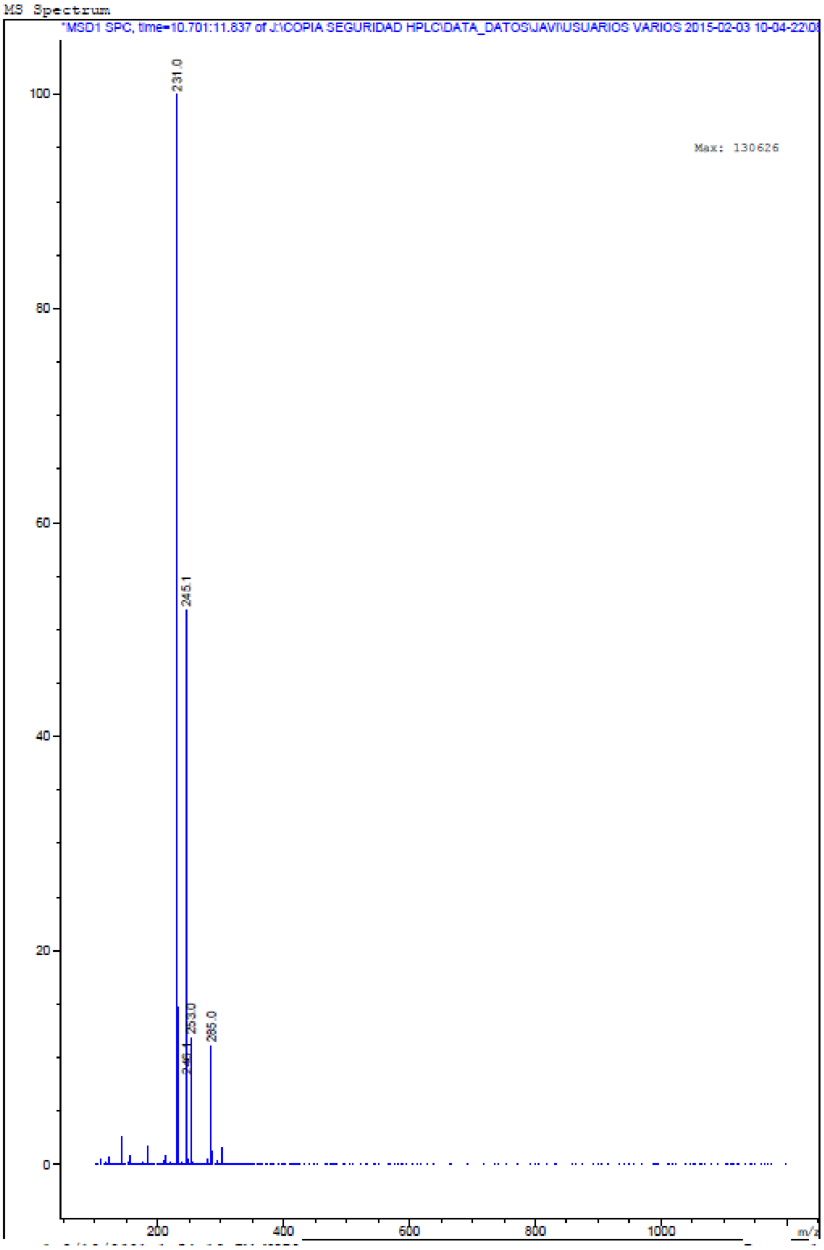

Compound **26** (UCM-1306)

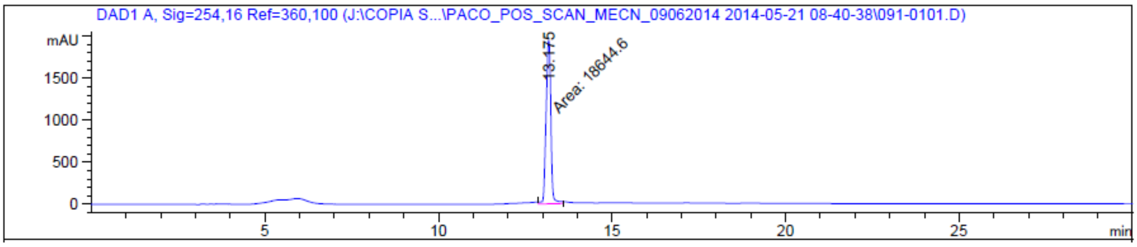

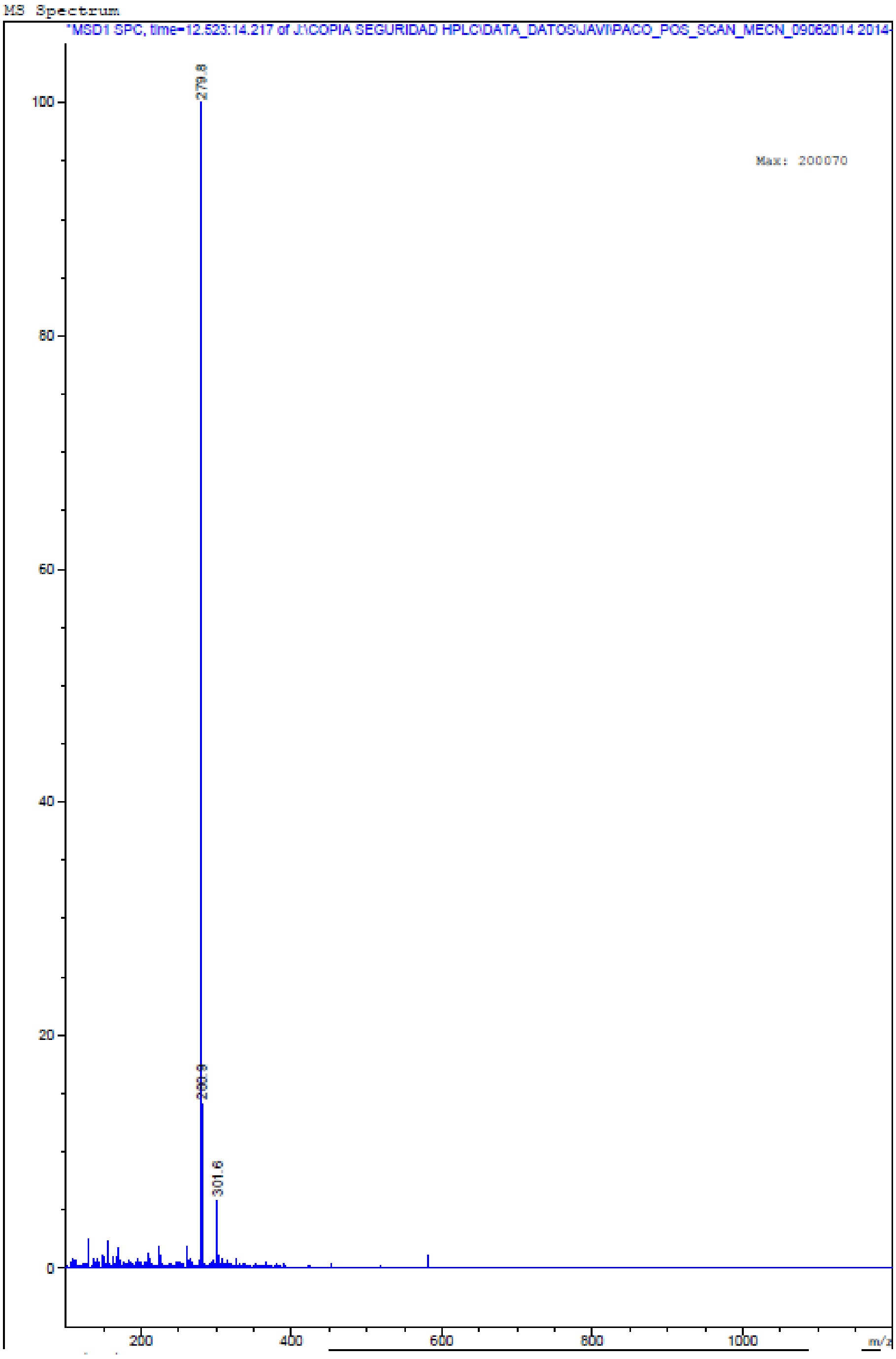

Compound (*R*)-**26**

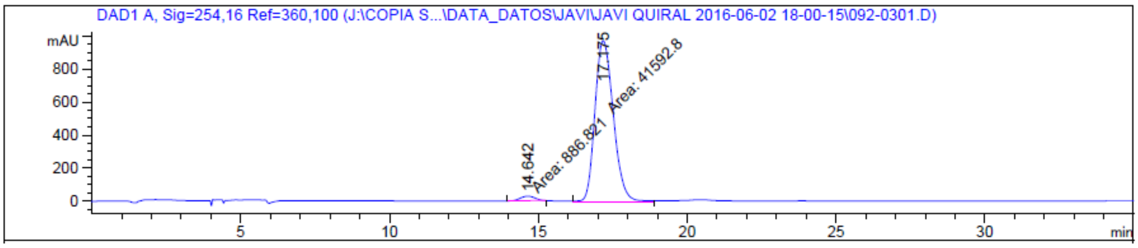

Signal 1: DAD1 A, Sig=254,16 Ref=360,100

Peak #	RetTime [min]	Type	Width [min]	Area [mAU*s]	Height [mAU]	Area %
1	14.642	MM	0.5503	886.82098	26.85841	2.0876
2	17.175	MM	0.7130	4.15928e4	972.26337	97.9124
Totals :				4.24796e4	999.12177	

Compound (*S*)-**26**

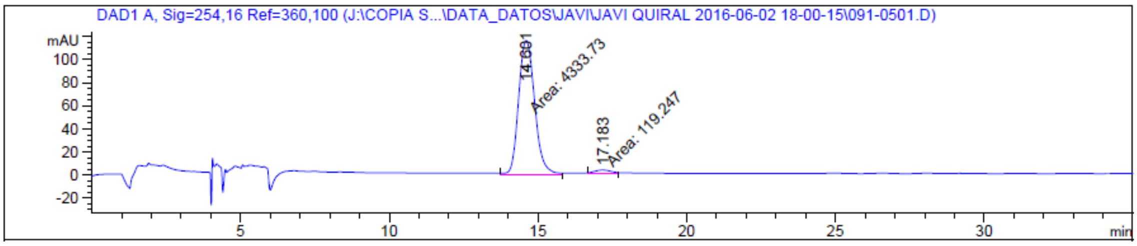

Signal 1: DAD1 A, Sig=254,16 Ref=360,100

Peak #	RetTime [min]	Type	Width [min]	Area [mAU*s]	Height [mAU]	Area %
1	14.601	MM	0.6236	4333.73486	115.81680	97.3221
2	17.183	MM	0.6592	119.24712	3.01480	2.6779
Totals :				4452.98199	118.83159	

6. References

1. Luo, J.; Preciado, S.; Larrosa, I. Overriding *ortho*-*para* Selectivity via a Traceless Directing Group Relay Strategy: The *meta*-Selective Arylation of Phenols. *J. Am. Chem. Soc.* **2014**, *136*, 4109–4112.

2. Wei, Y.; Yoshikai, N. Oxidative Cyclization of 2-Arylphenols to Dibenzofurans under Pd(II)/Peroxybenzoate Catalysis. *Org. Lett.* **2011**, *13*, 5504–5507.

3. Lee, J. J.; Kraus, G. A. Divergent Diels-Alder Methodology from Methyl Coumalate toward Functionalized Aromatics. *Tetrahedron Lett.* **2013**, *54*, 2366–2368.

4. Zhu, J.; Wang, J.; Dong, G. Catalytic Activation of Unstrained C(Aryl)–C(Aryl) Bonds in 2,2′-Biphenols. *Nature Chem.* **2019**, *11*, 45–51.

5. Senatore, R.; Malik, M.; Spreitzer, M.; Holzer, W.; Pace, V. Direct and Chemoselective Electrophilic Monofluoromethylation of Heteroatoms (*O*-, *S*-, *N*-, *P*-, *Se*-) with Fluoroiodomethane. *Org. Lett.* **2020**, *22*, 1345–1349.

6. Surya Prakash, G. K.; Weber, C.; Chacko, S.; Olah, G. A. New Electrophilic Difluoromethylating Reagent. *Org. Lett.* **2007**, *9*, 1863-1866.

7. Rama Raju, B.; Sarkar, S.; Chandramoulali Reddy, U.; Saikia, A. K. Cerium (IV) Triflate-Catalyzed Selective Oxidation of Sulfides to Sulfoxides with Aqueous Hydrogen Peroxide. *J. Mol. Catal. A-Chem.* **2009**, *308*, 169-173.

8. Ram Cho, G. Y.; Okamura, H.; Bolm, C. Synthesis and Palladium-Catalyzed Coupling Reactions of Enantiopure *p*-Bromophenyl Methyl Sulfoximine. *J. Org. Chem.* **2005**, 70, 2346–2349.

9. Dannenberg, C. A.; Bizet, V.; Bolm, C. Direct Access to *N*-Alkylsulfoximines from Sulfides by a Sequential Imidation/Oxidation Procedure. *Synthesis* **2015**, *47*, 1951–1959.

10. Liao, S.; Liang, Y.; Zhang, Z.; Li, J.; Wang, J.; Wang, X.; Dou, G.; Zhang, Z.; Liu, K. In Vitro Metabolic Stability of Exendin-4: Pharmacokinetics and Identification of Cleavage Products. *PLoS One* **2015**, *10*, 1-18.
